# Plasticity of the β-Trefoil Protein Fold in the Recognition and Control of Invertebrate Predators and Parasites by a Fungal Defence System

**DOI:** 10.1371/journal.ppat.1002706

**Published:** 2012-05-17

**Authors:** Mario Schubert, Silvia Bleuler-Martinez, Alex Butschi, Martin A. Wälti, Pascal Egloff, Katrin Stutz, Shi Yan, Iain B. H. Wilson, Michael O. Hengartner, Markus Aebi, Frédéric H.-T. Allain, Markus Künzler

**Affiliations:** 1 Institute of Molecular Biology and Biophysics, ETH Zürich, Zürich, Switzerland; 2 Institute of Microbiology, ETH Zürich, Zürich, Switzerland; 3 Institute of Molecular Life Sciences, University of Zürich, Switzerland; 4 Department of Chemistry, University of Natural Resources and Life Sciences (BOKU), Vienna, Austria; University of Massachusetts Medical School, United States of America

## Abstract

Discrimination between self and non-self is a prerequisite for any defence mechanism; in innate defence, this discrimination is often mediated by lectins recognizing non-self carbohydrate structures and so relies on an arsenal of host lectins with different specificities towards target organism carbohydrate structures. Recently, cytoplasmic lectins isolated from fungal fruiting bodies have been shown to play a role in the defence of multicellular fungi against predators and parasites. Here, we present a novel fruiting body lectin, CCL2, from the ink cap mushroom *Coprinopsis cinerea*. We demonstrate the toxicity of the lectin towards *Caenorhabditis elegans* and *Drosophila melanogaster* and present its NMR solution structure in complex with the trisaccharide, GlcNAcβ1,4[Fucα1,3]GlcNAc, to which it binds with high specificity and affinity *in vitro*. The structure reveals that the monomeric CCL2 adopts a β-trefoil fold and recognizes the trisaccharide by a single, topologically novel carbohydrate-binding site. Site-directed mutagenesis of CCL2 and identification of *C. elegans* mutants resistant to this lectin show that its nematotoxicity is mediated by binding to α1,3-fucosylated N-glycan core structures of nematode glycoproteins; feeding with fluorescently labeled CCL2 demonstrates that these target glycoproteins localize to the *C. elegans* intestine. Since the identified glycoepitope is characteristic for invertebrates but absent from fungi, our data show that the defence function of fruiting body lectins is based on the specific recognition of non-self carbohydrate structures. The trisaccharide specifically recognized by CCL2 is a key carbohydrate determinant of pollen and insect venom allergens implying this particular glycoepitope is targeted by both fungal defence and mammalian immune systems. In summary, our results demonstrate how the plasticity of a common protein fold can contribute to the recognition and control of antagonists by an innate defence mechanism, whereby the monovalency of the lectin for its ligand implies a novel mechanism of lectin-mediated toxicity.

## Author Summary

All multicellular organisms have developed mechanisms to defend themselves against predators, parasites and pathogens. As a common mechanism, animals, plants and fungi use a large arsenal of carbohydrate-binding proteins (lectins) to protect themselves from predation and parasitism. The success of this type of innate defence mechanism critically depends on the diversity of specific recognition of foreign carbohydrate structures by the host lectins. In this study, we use NMR structure determination to show that part of this diversity is created by the plasticity of common protein folds. The identified fungal lectin that is toxic to nematodes and insects, adopts a common lectin fold but is remarkable in terms of its specificity and affinity for the recognized foreign carbohydrate structure, the number and location of the carbohydrate binding sites on the protein and the degree of oligomerization. Since the identified *in vivo* target of the fungal lectin is characteristic for invertebrates, our results may be exploited to develop novel approaches for the control of animal and human parasites.

## Introduction

Adequate and efficient defence mechanisms to protect an organism's integrity and survival have been essential for the evolution of multicellularity since loss of individual cells may be detrimental for a multicellular organism. Any defence mechanism thereby critically relies on the ability to discriminate between self and non-self. Since all living cells display specific carbohydrate structures on their surface [1], glycans have been used for the recognition of non-self since the beginning of multicellular life [2]. Accordingly, many of the proteins that are able bind to specific carbohydrate structures, commonly referred to as lectins, have been implicated in defence, mainly in the innate immune systems of animals which is considered an ancestral defence mechanism and a first and immediate line of defence against potentially harmful microorganisms [3]. These lectins are either membrane-bound or secreted and localize to the interface between the host and the environment where they bind to microorganism-associated carbohydrates and function either as receptors triggering the expression of host immune effectors, by opsonizing the microorganisms for host immune effectors or immune cells (reviewed in [4]) or as direct immune effectors by killing the microorganism upon binding [5]–[8]. In analogy to latter function of combining non-self recognition and killing, plants use insecticidal lectins to defend themselves against herbivorous insects [9]. Recently, a group of fungal lectins, commonly referred to as fruiting body lectins, has been shown to play a role in the defence of multicellular fungi against predators and parasites based on their toxicity to various model organisms [10]–[15]. According to the above role of lectins in defence, most defence lectins should be specific for carbohydrate structures that do not exist in the host (are non-self) and are characteristic for the target organism. To date, only very few target carbohydrate structures or glycoconjugates of such lectins involved in innate defence mechanisms have been identified and their recognition by the lectin investigated at molecular level [5], [8], [10], [16], [17].

In organisms lacking an antibody-based adaptive immunity, such a lectin-based defence strategy critically relies on a large diversity in carbohydrate specificities. This diversity can be achieved either by diversification on the level of lectin folds and/or by the plasticity of a common lectin fold. The known fruiting body lectins belong to six structural families [14] of which the β-propeller-fold lectins, actinoporin-like lectins, galectins and β-trefoil (ricin B or R-type) lectins [18] are the most prominent ones. Some of these lectins are multidomain proteins harbouring in addition a cysteine protease/dimerization domain (R-type *Marasmius oreades* agglutinin [MOA] and *Polyporus squamosus* lectin [PSL]) [19], [20] or a pore-forming module (R-type *Laetiporus sulphureus* lectin [LSL]) [21]. In the first case, it was demonstrated that both domains are required for toxicity [10] suggesting that the lectin domain guides the catalytic domain to specific target structures. However, most lectins implicated in the defence of plants and fungi are composed just of lectin domains and contain multiple binding sites for either the same or different carbohydrate structures. For some of these lectins it has been demonstrated that this multivalency is essential for their toxicity [22]. These results suggest that lectin-mediated toxicity involves crosslinking of glycoconjugates but the exact mechanism remains unclear.

We describe the identification and characterization of a novel, monovalent lectin, CCL2, from fruiting bodies of the ink cap mushroom *Coprinopsis cinerea* and present the NMR structure of CCL2 in ligand-free form and in complex with its *in vivo* ligand. The lectin was found to bind specifically and with an atypical high affinity to Fucα1,3-modified core N-glycans *in vitro*, using a single, topologically novel binding site on its β-trefoil fold. N-glycans carrying such a modification are characteristic for invertebrates but absent from fungi. We applied biotoxicity assays to demonstrate toxicity towards two model invertebrates. In accordance with the *in vitro* binding data, the nematotoxicity of CCL2 was dependent on core α1,3-fucosylation of *C. elegans* N-glycans on intestinal proteins of the nematode. These results show how multicellular organisms exploit the plasticity of a common protein fold to create a novel lectin specificity and an alternative mechanism of lectin-mediated toxicity for defence.

## Results

### Identification, cloning and expression of CCL2 from *Coprinopsis cinerea*

**Figure 1:**
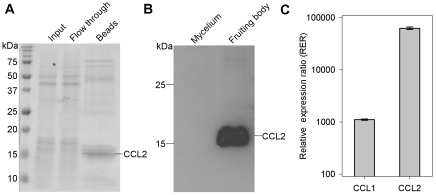
Isolation and differential expression of *C. cinerea* CCL1 and CCL2. (A) Specific binding of CCL2 to horseradish peroxidase (HRP). Coomassie-stained SDS-PAGE showing Input, Flow through and Bound (Beads) fractions of a soluble protein extract from *C. cinerea* fruiting bodies upon affinity-chromatography using immobilized HRP. The Bound fraction was released by boiling the HRP-sepharose beads in Lämmli sample buffer. The loaded protein amount of the Bound fraction (Beads) corresponds to two equivalents of Input and Flow through fractions. Sizes of the marker proteins are indicated. (B) Immunoblot comparing expression levels of CCL2 between vegetative mycelium and fruiting bodies of *C. cinerea*. Equal amounts of total protein were loaded in each lane. A polyclonal antiserum raised in rabbits against purified CCL2 was used for detection. (C) Comparisons of relative expression ratio (or fold up-regulation) of the genes encoding CCL1 and CCL2 by qRT-PCR in fruiting bodies relative to vegetative mycelium. Error bars represent standard deviation of the mean.

We detected a soluble 15 kDa protein from fruiting bodies of the model mushroom *C. cinerea* by virtue of its binding to horseradish peroxidase (HRP) in immunoblots. The protein was present in extracts from fruiting bodies but not from vegetative mycelium, indicating a fruiting body-specific expression. We isolated the protein using HRP-affinity chromatography (Figure 1A) and identified it as hypothetical protein CC1G_11781 of *C. cinerea* strain Okayama7 by MALDI-MS/MS. Since the protein, termed CCL2 (*Coprinopsis cinerea* lectin 2), was extracted from fruiting bodies of the *C. cinerea* strain AmutBmut (Swamy et al 1984), the respective genomic locus of strain AmutBmut was cloned and sequenced. This sequence served as a basis for the cloning of the respective cDNA from total RNA isolated from AmutBmut fruiting bodies. A second cDNA, coding for an isoprotein (52% identity; Table S1), termed CCL1 (*Coprinopsis cinerea* lectin 1) (CC1G_11778), was cloned and sequenced accordingly. The two proteins are predicted to contain neither a signal sequence for classical secretion nor N-glycosylation sites. The cDNAs coding for CCL1 and CCL2 were cloned in pET expression vectors and the proteins were expressed in the cytoplasm of *E. coli* BL21(DE3). The recombinant proteins were highly expressed and soluble (Figure S1) and versions containing eight N-terminal His-residues were purified using metal-affinity chromatography. Size exclusion chromatography of the purified CCL2 showed that the protein exists as a monomer in solution (Figure S2).

Immunoblots using a CCL2-specific antiserum confirmed that CCL2 is abundant in fruiting bodies and absent from vegetative mycelium (Figure 1B). The differential expression of both CCL2 and CCL1 was quantified at the transcript level by qRT-PCR (Figure 1C). The results indicate that the mRNA levels of CCL1 and CCL2 are more than 1000-fold and 60,000-fold, respectively, higher in fruiting bodies than in the vegetative mycelium.

### Carbohydrate-binding activity and specificity of CCL2

**Figure 2:**
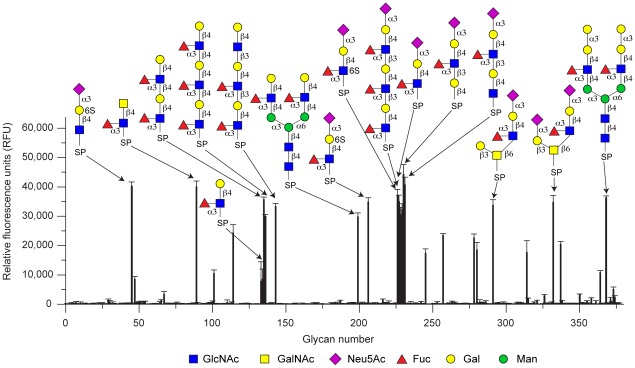
Carbohydrate-binding specificity of CCL2. Fluorescently labeled CCL2 was analyzed for binding to the mammalian glycan array (V3.1) of the Consortium for Functional Glycomics (CFG). Results shown are averages of triplicate measurements of fluorescence intensity at a lectin concentration of 200 µg/ml. Error bars indicate the standard deviations of the mean. Glycan structures are depicted for those epitopes with highest relative fluorescence. The raw data and the entire list of glycans with the respective spacers can be found on the CFG homepage [http://functionalglycomics.org/] or in Tables S2 and S3. Binding of 6'sulfo-sialyllactose (glycan #45) is likely to be an artifact since it is also bound by fucose-binding lectin AAL [http://functionalglycomics.org/].

**Figure 3:**
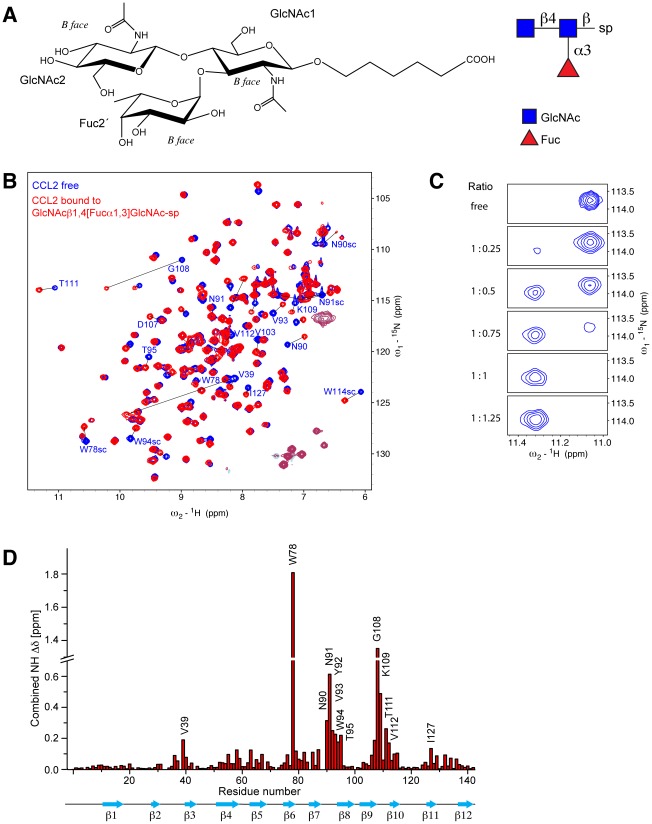
Refining the specificity of the CCL2 lectin. (A) The chemical and schematic structure of the fucosylated chitobiose (GlcNAcβ1,4[Fucα1,3]GlcNAc-spacer) that was used as ligand for binding studies and structure determination. Indicated is also the B face that is defined as the face on which the carbons are numbered in an anticlockwise order [69]. (B) Chemical shift deviations upon complex formation at a protein concentration of 0.4 mM at pH 5.7. Overlay of ^15^N-HSQC spectra of free CCL2 (blue) and CCL2 bound to one equivalent of fucosylated chitobiose (red). (C) Titration of the amide signal of T111 in CCL2 with fucosylated chitobiose using ^15^N-HSQC spectra. The protein∶ligand ratio is displayed on the left. (D) Plot of the chemical shift differences between free and bound CCL2 ( δ = [ δ_HN_^2^+(δ_N_/R_scale_)^2^ ]^1/2^, R_scale_ = 5).

Based on the binding to the plant glycoprotein HRP and a similar expression pattern as previously characterized lectins from this organism [23], [24], we hypothesized that CCL2 is a lectin. Fluorescently labeled CCL2 was used to probe a glycan array offered by the Consortium of Functional Glycomics (CFG) (Figure 2 and Table S2), confirming that CCL2 is a lectin that binds specifically to carbohydrate structures containing the Fucα1,3GlcNAc motif e.g. the Lewis^X^ antigen (Galβ1,4[Fucα1,3]GlcNAc; Glycan structure #133/134 on the array). The disaccharide Fucα1,3GlcNAc alone, however, showed a very low fluorescence, suggesting that at least a trisaccharide was required for efficient binding. Glycan array analysis with purified CCL1 (Figure S3 and Table S3) yielded almost the same results as with CCL2. The binding specificity of CCL2 was further studied with several carbohydrates *in vitro* by NMR spectroscopy and isothermal titration calorimetry (ITC) as summarized in Table 1. The trisaccharide Lewis^X^ bound with a moderate K_D_ of 456 µM and the NMR spectra displayed intermediate to slow exchange behavior during the titration, whereas the binding of sialylated Lewis^X^, was slightly better by a factor of ∼3. However, fucosylated chitobiose (GlcNAcβ1,4[Fucα1,3]GlcNAc-spacer; Figure 3A), absent on the glycan array, had by far the highest affinity among the tested oligosaccharides with a K_D_ of 1.4 µM (Table 1 and Figure S4). Monitoring the binding by NMR spectroscopy revealed large chemical shift changes under the slow exchange regime (Figures 3B and C). Binding occurs with a stoichiometry of 1∶1 and no further changes were observed by adding an excess of ligand (1∶50). The largest chemical shift deviations occurred at residues W78, N90-T95, G108 and K109 (Figure 3D).

**Table 1:** Binding of CCL2 wild-type to different carbohydrates and CCL2 variants to GlcNAcβ1,4[Fucα1,3]GlcNAcβ1-sp (sp: spacer O-[CH_2_]_5_COOH) measured with isothermal titration calorimetry and NMR spectroscopy at 299K.

Carbohydrate	K_D_ (µM)	Fold affinity decrease	ΔH (kJ/mol)	−TΔS (kJ/mol)	NMR titration
Fucα1,3GlcNAc-OMe	>500				no binding
Galβ1,4[Fucα1,3]GlcNAcβ1-OMe (Le^X^-trisaccharide)	456				nd^a^
Galβ1,4[Fucα1,3]GlcNAcβ1,4Gal (Le^X^-tetrasaccharide)	nd^a^				large chemical shift deviations, slow to intermediate exchange
Neu5Acα2,3Galβ1,4[Fucα1,3]GlcNAcβ1-OMe (Sialyl Le^X^)	162				nd^a^
GlcNAcβ1,4[Fucα1,3]GlcNAcβ1-sp^a^ (Fucosylated chitobiose)	1.4		−49.8	16.3	large chemical shift deviations, slow exchange
Neu5Acα2,3Galβ1,4Glc (Sialyl lactose)	nd^a^				no binding

Protein	KD (µM)	Fold affinity decrease	ΔH (kJ/mol)	−TΔS (kJ/mol)	NMR titration
Wild type	1.4		−49.8	16.3	
Y57A	10.9	7.8	−44.4	15.9	
W78A	62.9	44.9	−35.6	11.7	
L87A	26.3	17.8	−43.5	17.2	
N90A	6.7	4.6	−43.1	13.4	
N91A	0.18^b^	0.13	−57.7	18.8	
Y92A	>500				
V93A	27.2	19.4	−39.8	13.4	
W94A	310	221	−38.1	18.0	
K109A	1.5	1.1	−47.7	14.2	

a nd: not determined.

b The increased affinity of N91A might be an artifact caused by interaction of the artificial carbohydrate spacer O-(CH_2_)_5_-COOH with residue 91. Whereas the spacer might sterically clash with N91, Ala in this position could form favorable van-der-Waals interactions. In the case of natural N-glycans, where the reducing GlcNAc is linked to Asn of a glycoprotein projecting away from CCL2 (upper right corner of Figure 5D), Asn is likely to be favored at this position of CCL2 due to the potential formation of H-bonds.

### The NMR structure of CCL2 reveals a β-trefoil fold

**Figure 4:**
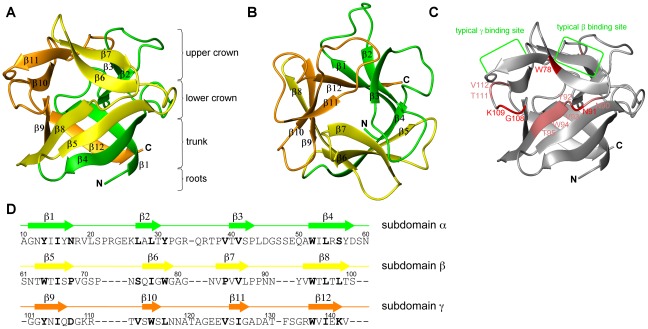
Solution structure of the CCL2 lectin in the absence of a ligand determined by NMR spectroscopy. The side (A) and top (B) view of the most representative structure out of 20 structures is shown. The three pseudo symmetric sections of the β-trefoil fold corresponding to residues S9–N60, S61–S100 and G101–V142 are colored green, yellow and orange, respectively. Characteristic regions are labeled according to Renko et al. for better orientation [26]. (C) Chemical shift deviations mapped on the structure of CCL2 in the same orientation as in A. Chemical shifts of residues in red experience a combined NH chemical shift deviation >0.4 ppm, for residues in pink >0.15 ppm. (D) Secondary structure and subdomain borders displayed on the protein sequence. The same color code as in A and B is used. Bold residues are forming the hydrophobic core of the protein.

Since CCL2 did not show sequence similarity to any known structure we determined the 3D structure of CCL2 by NMR spectroscopy (Figure 4). CCL2 adopts a β-trefoil fold consisting of three β-β-β-β repeats with a pseudo C_3_ symmetry. β_1_ and β_4_ of each repeat form together a β-barrel whereas β_2_ and β_3_ adopt a β-hairpin that usually harbors the carbohydrate-binding site [25]. The β-trefoil structure can be compared to a tree [26] in which the trunk is represented by the β-barrel (β_1_ and β_4_, β_5_ and β_8_, β_9_ and β_12_), the roots are formed by the N- and C-terminus together with the two loops β_4_–β_5_ and β_8_–β_9_, the upper crown is formed by the three β-hairpins (β_2_ and β_3_, β_6_ and β_7_, β_10_ and β_11_) and the lower crown by the loops connecting the β-barrel with the β-hairpin loops. As can be seen from Figures 4B and D, the loops β6–β7 and β7–β8 in subdomain β are shorter than in the other subdomains. In addition, subdomain β shows a deviation from the most characteristic feature of β-trefoil proteins, the QxW motif in each subdomain [25]. Subdomain β contains a YxW instead. A search for structurally similar proteins revealed a large number of bacterial, fungal and plant toxins displaying high structural similarity but low sequence identity (Table S4).

The 3D structure was used to visualize the largest chemical shift deviations from the titration experiment with GlcNAcβ1,4[Fucα1,3]GlcNAc (from Figure 3D) in Figure 4C. The largest deviations occur at the interface between subdomain β and γ, mainly on strand β8 and its unusually short preceding loop β7–β8 (β subdomain) and in the β9–β10 loop (γ subdomain). This arrangement does not correspond to the typical binding interface of β-trefoil lectins and therefore we decided to investigate this new binding mode.

### Structure of CCL2 in complex with fucosylated chitobiose

**Figure 5:**
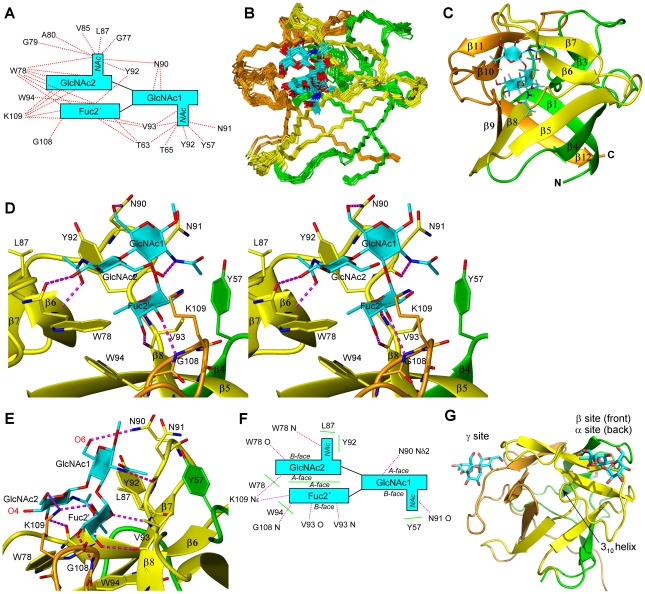
NMR solution structure of the CCL2 lectin in complex with fucosylated chitobiose (GlcNAcβ1,4[Fucα1,3]GlcNAc). (A) Intermolecular NOEs observed in a 3D ^13^C F1-edited F3-filtered HSQC-NOESY spectrum in a schematic presentation. (B) Structural ensemble of 20 structures of the protein backbone and the carbohydrate in cyan. The subunits α, β and γ are colored green, yellow and orange, respectively. The orientation is identical to Figure 4. (C) Ribbon presentation of the most representative structure. (D) Stereo view of the carbohydrate recognition site. Potential intermolecular hydrogen bonds are shown with dashed magenta lines. (E) Details of the interaction site illustrating how the trisaccharide is recognized by hydrogen bonds. (F) Summary of the interactions between the trisaccharide and CCL2. Potential H-bonds are indicated as dotted lines in magenta and hydrophobic interactions by green lines. (G) Crystal structure of the β-trefoil domain of the fungal lectin MOA in complex with the trisaccharide Galα1,3[Fucα1,2]Gal [19] showing all three occupied canonical binding sites (pdb∶3EF2). For better comparison, the same orientation and colors as in panel B and C were used.

We solved the 3D structure of the complex between CCL2 and fucosylated chitobiose (GlcNAcβ1,4[Fucα1,3]GlcNAcβ–sp) by NMR spectroscopy. 82 intermolecular distance restraints that are well distributed over the binding interface (Figure 5A) were derived from a 3D ^13^C F1-edited F3-filtered HSQC-NOESY [27] spectrum (Figure S5). A precise structural ensemble of the complex was obtained (Figure 5B and Table 2). The carbohydrate is bound at the interface of the subdomains β and γ in the lower crown (Figure 5C), in particular between the β-strands β_6_ and β_8_ and the linker β_7_–β_8_ of the β subdomain and the loop between β_9_–β_10_ of the γ subdomain. Compared to the canonical binding sites (Figure 5G and Figure S6) this is a very unusual binding location for ricin B type lectins.

**Table 2:** NMR structure determination statistics of CCL2 in the free form and in complex with the fucosylated chitobiose (GlcNAcβ1,4[Fucα1,3]GlcNAc-spacer, the spacer [CH_2_]_5_COOH was truncated in the structure calculations to a methyl group.).

		CCL2–carbohydrate complex	
	CCL2 free	CCL2	carbohydrate
**NMR distance and dihedral constraints**			
Distance restraints			
Total NOE	2514	2054	42
Intra-residue	482	446	23
Inter-residue	2032	1608	19
Sequential (|*i*−*j*| = 1)	593	489	9
Nonsequential (|*i*−*j*|>1 )	1439	1119	10
Hydrogen bonds	46	49	–
Protein–carbohydrate intermolecular		82	
Total dihedral angle restraints	186	178	
Protein			
φ	91*	85*	
ψ	95	91	
Carbohydrate			
HN-CO peptide bonds of acetamido			2
**Structure statistics**			
Violations (mean and s.d.)			
Number of distance constraint violations >0.2 Å	0.42±0.64	0.45±0.61	
Number of dihedral angle violations >5°	0.05±0.22	4.75±1.48	
Max. dihedral angle violation (°)	2.6±3.4	17.4±8.5	
Max. distance constraint violation (Å)	0.24±0.07	0.20±0.04	
Deviations from idealized geometry			
Bond lengths (Å)	0.010	0.010	
Bond angles (°)	2.29	2.38	
Average pairwise r.m.s. deviation** (Å)			
Protein (residues G22-V153)			
Heavy	0.98±0.14	1.18±0.15	
Backbone	0.45±0.10	0.71±0.13	
carbohydrate			
All glycan heavy			0.47±0.21
Complex			
Protein and carbohydrate heavy		1.17±0.15	

* Phi values for prolines were omitted.

** Pairwise r.m.s. deviation was calculated among 20 refined structures.

The well-defined trisaccharide is oriented such that GlcNAc2 (see Figure 3A for nomenclature of the individual sugars in the trisaccharide) stacks on top of Fuc2′ thereby locking the conformational freedom of the glycan resulting in a narrow clustering of the glycosidic angles (Figure S7). The hydrophobic B-face of Fuc2′ is oriented towards the protein (bottom) and the hydrophobic B-face of GlcNAc2 towards the solution (top). In this orientation GlcNAc1 is tilted horizontally such that its B-face is located on the back contacting the protein. Contacts to all three sugar units are mediated by a large number of potential H-bonds and hydrophobic interactions (Figures 5D–F and Table 3).

**Table 3:** Potential intermolecular protein–carbohydrate hydrogen bonds based on the orientations and positions of the carbohydrate in the complex structure.

Hydrogen bonds	Occurence in ensemble <3.2 Å	Supporting chemical shift
W78 N – GlcNAc2 O7	15/20	largest NH chemical shift change (Figures 3B and D)
W78 O – GlcNAc2 O3	1/20	small C′ chemical shift change δ (C′)_free_ = 175.9 ppm; δ (C′)_bound_ = 176.1 ppm
N90 ND2 – GlcNAc1 O6	6/20	large NH chemical shift change (Figure 3B)
N91 O – GlcNAc1 N2	19/20	moderate C′ chemical shift change δ (C′)_free_ = 173.3 ppm; δ (C′)_bound_ = 173.8 ppm
V93 N – Fuc2′ O5	5/20	moderate NH chemical shift change (Figures 3B and D)
V93 O – Fuc2′ O4	20/20	largest C′ chemical shift change δ (C′)_free_ = 174.4 ppm; δ (C′)_bound_ = 172.2 ppm
G108 N – Fuc2′ O3	12/20	second largest NH chemical shift change (Figures 3B and D)
G108 N – Fuc2′ O4	17/20	second largest NH chemical shift change (Figures 3B and D)
K109 NE – Fuc2′ O2	4/20	
K109 NE – Fuc2′ O3	3/20	
K109 NE – GlcNAc2 O4	4/20	
K109 NE – GlcNAc2 O6	10/20	

Note that no intermolecular hydrogen bond constraints were used during the calculations.

The specific recognition of each sugar unit can be described as follows: Fuc2′ approaches the edge of β-strand β8 and the tip of loop β9–β10 with its b-face and bridges subdomain β and γ in this way (Figures 5C–F). In this orientation O4 and O5 face down and are specifically recognized by H-bonds to the main chain (V93 HN and O) of the unusually short loop between strands β7 and β8 (Figure 5E). The equatorial hydroxyl groups of O3 and O2 form H-bonds to G108 HN (second largest chemical shift deviation, Figure 3D) and Lys109 NH_3_^+^. In addition the hydrophobic methyl group and the axial H2, both facing downwards, form hydrophobic contacts with Trp94/Trp95 and Val93, respectively. The methyl group is located above the ring of W94 enabling favorable Me-π interactions that are supported by an upfield shift of the H6 resonance (−0.18 ppm; Table S5). In total all characteristic groups of Fuc2′ are specifically recognized by 4 H-bonds, hydrophobic and π interactions. Both the location of Fuc2′ at the subdomain interface and the recognition by three H-bonds to the main-chain are unprecedented in all ricin B type lectin complex structures. GlcNAc1 is specifically recognized at its equatorial acetamido group by a H-bond of its HN to Asn91 O, and at O6 by an H-bond to the side chain of Asn90. The acetamido group forms hydrophobic interactions to Val93 and Me-π interactions with Tyr57 which is supported by an upfield shift of the methyl ^1^H resonance (−0.24 ppm). Its hydrophobic b-face packs to the Tyr92 side chain. Only a GlcNAc would be recognized at this position since the equatorial orientation of the acetamido and the CH_2_OH group are necessary for their recognition by H-bonds and the equatorial positioning of O3 and O4 is required for the stacking between Fuc2′ and GlcNAc2. GlcNAc2 is mainly recognized via its acetamido group by an H-bond to Trp78 HN (supported by the largest HN chemical shift deviation, Figure 3D), hydrophobic interactions of the methyl with Leu87 and a stacking of the entire acetamido group to the ring of Tyr92 (Figure 5D). Me-π interactions to Y92 are supported by an upfield shift (−0.24 ppm). GlcNAc2 that stacks on top of Fuc2′ is slightly laterally shifted exposing the hydrophobic H4 facing downwards. H4 is located on top of the Trp94 ring and favorable H-π interactions are supported by an upfield shift of its resonance (−0.39 ppm; Table S5). Two additional potential H-bonds are observed in some structures of the ensemble: between the carbonyl of W78 and O3 of GlcNAc2 and between K109 NH_3_^+^ and GlcNAc2 O6. In summary, GlcNAc1 and Fuc2′ are specifically recognized by interactions to almost all of their functional groups whereas the recognition of GlcNAc2 is more relaxed. It is mainly recognized at its equatorial acetamido group attached to C2. This residue must be able to stack to Fuc2′ in order to properly position the acetamido group; both GlcNAc and GalNAc fulfill this requirement and will be recognized in this position. Accordingly, CCL2 binds to fucosylated LacdiNAc (GalNAcβ1,4[Fucα1,3]GlcNAc; Glycan structure #89) on the array.

**Figure 6:**
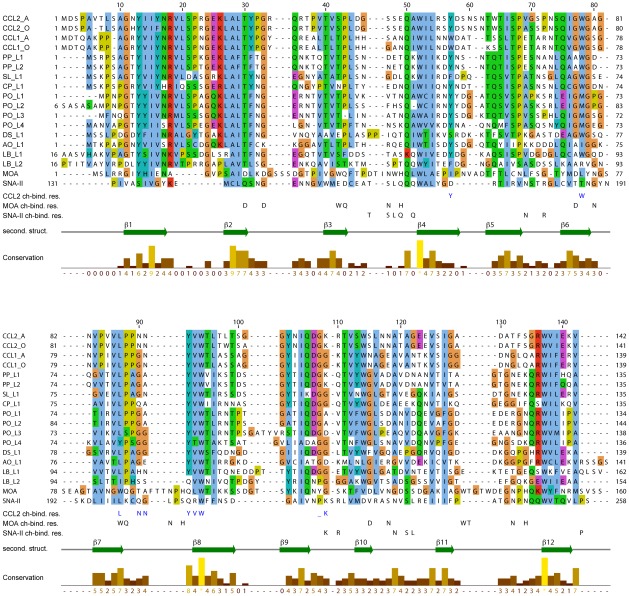
Sequence conservation among CCL2-like proteins and comparison to two typical representatives of fungi and plants. Sequence alignment of several fungal and plant R-type lectins. CCL2_A: CCL2 of *C. cinerea* strain AmutBmut; CCL2_O: CCL2 of *C. cinerea* strain Okayama7; CCL1_A: CCL1 of *C. cinerea* strain AmutBmut; CCL1_O: CCL1 of *C. cinerea* strain Okayama7; PP_L1: *Postia placenta* lectin 1 (Pospl1_130016); PP_L2: *Postia placenta* lectin 2 (Pospl1_121916); SL_L1: *Serpula lacrymans* lectin 1 (SerlaS7_144703); CP_L1: *Coniophora puteana* lectin 1 (Conpu1_119225); PO_L1: *Pleurotus ostreatus* lectin 1 (PleosPC9_89828); PO_L2: *Pleurotus ostreatus* lectin 2 (PleosPC15_1043947); PO_L3: *Pleurotus ostreatus* lectin 3 (PleosPC9_64199); PO_L4: *Pleurotus ostreatus* lectin 4 (PleosPC15_1065820); DS_L1: *Dicomitus squalis* lectin 1 (Dicsq1); AO_L1: *Arthrobotrys oligospora* lectin 1 (s00075g2); LB_L1: *Laccaria bicolor* lectin 1 (Lbic_330799); LB_L2: *Laccaria bicolor* lectin 2 (Lbic_327918); MOA: *Marasmius oreades* agglutinin; SNA-II: *Sambucus nigra* agglutinin/ribosome inactivating protein type II. The distantly related canonical R-type lectins MOA (fungal, 14% sequence identity) and SNA-II (plant, 13% sequence identity) were included in the alignment based on comparison of their 3D structures [70], [71]. The Clustal X color scheme was used. Residues involved in the carbohydrate recognition are indicated at the bottom for CCL2, MOA and SNA-II. The secondary structure of CCL2 and the conservation is indicated as well. The alignment was generated with Jalview [72].

The large number of H-bonds to the main chain is remarkable. The unusually short β7–β8 loop contributes three and the β9–β10 loop one such H-bonds. Since the protein main chain does not change upon binding, part of the recognition pattern on the protein is preformed. However, the lengths and conformations of these loops are a special feature of CCL2 homologues as illustrated on a structure-based alignment (Figure 6) and are not conserved in the β-trefoil fold. Note also that the short β7–β8 loop lacks the typical 3_10_ helix segment as seen for example in the structurally most closely related R-type lectin MOA (Figure 5G) which would clash with the carbohydrate.

### Comparison of binding affinity and thermodynamics with other lectins

**Figure 7:**
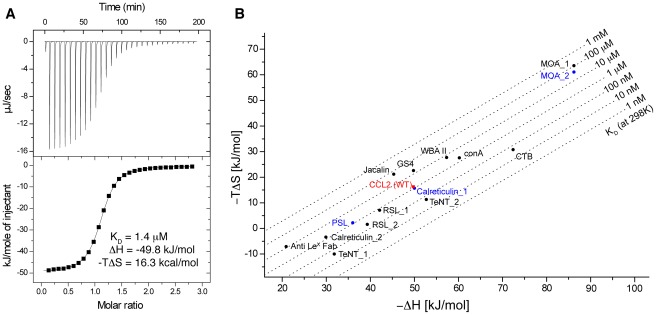
Thermodynamic binding parameters. (A) ITC experiment of wild type CCL2 binding to GlcNAcβ1,4[Fucα1,3]GlcNAc-spacer. The raw calorimetric output is shown on top, the fitted binding isotherm at the bottom. The protein concentration in the cell was 70 µM, carbohydrate concentration in the syringe was 2.4 mM. (B) Thermodynamic binding parameters of CCL2 (in red) in comparison to other lectins with a focus on high affinity binding. Anti Le^X^ Fab: Fab fragment of the monoclonal antibody 291-2G3-4; ConA: concavalin A from jack bean seeds (*Canavalia ensiformis*); CTB: cholera-toxin B subdomain; GS4: *Griffonia simplicifolia* lectin 4; MOA: *Marasmius oreades* agglutinin; RSL: *Ralstonia solanacearum* fucose-binding lectin; TeNT: tetanus neurotoxin; WBA II: winged bean (*Psophocarpus tetragonolobus*) acidic agglutinin. For simplicity, lectins that use Ca^2+^ for carbohydrate recognition are not displayed. Details for each correlation are found in Table S6. Data points in blue are discussed in the text.

The interaction of CCL2 with GlcNAcβ1,4[Fucα1,3]GlcNAc is governed by a large ΔH gain of −50 kJ mol^−1^ at the expense of 16 kJ mol^−1^ for −TΔS (Figure 7A). The thermodynamicbinding parameters are comparable to those of other high affinity lectins in Figure 7B (Table S6). In contrast to typical lectin interactions with medium affinity CCL2 uses an unusually large number of H-bonds (5–7 to backbone, 5 to side chain) and hydrophobic contacts (Trp78, Tyr92 and Trp94) for recognition of its target. A comparable number and kind of contacts is only found for few high affinity lectin interactions with a comparable K_D_∼1 µM. Interestingly, the calreticulin interaction with Glcα1,3Manα1,2Manα1,2Man with a K_D_ of 0.77 µM is governed by almost identical thermodynamic values [28], whereas the structurally closely related R-type lectin PSL [20] that binds to 6'sialyl lactose with a K_D_ of 1.3 µM [29] displays a moderately favored enthalpy but almost no entropic penalty. Both lectins use a similar number of direct H-bonds for their target recognition as CCL2 does: 10 (2 to backbone, 8 to side chain) and 9 (4 to the backbone, 5 to side chains), respectively, and a comparable amount of hydrophobic interactions.

### Biotoxicity of CCL2 against invertebrates and its dependence on specific carbohydrate binding

We tested the toxicity of CCL2 against four model organisms: the nematode *Caenorhabditis elegans*, the insects *Aedes aegypti* and *Drosophila melanogaster*, and the amoeba *Acanthamoeba castellanii*. The biotoxicity assays were performed either by feeding the test organisms with *E. coli* expressing the recombinant lectin as described previously [30], or by adding the purified lectin to the food source of the organisms.

**Figure 8:**
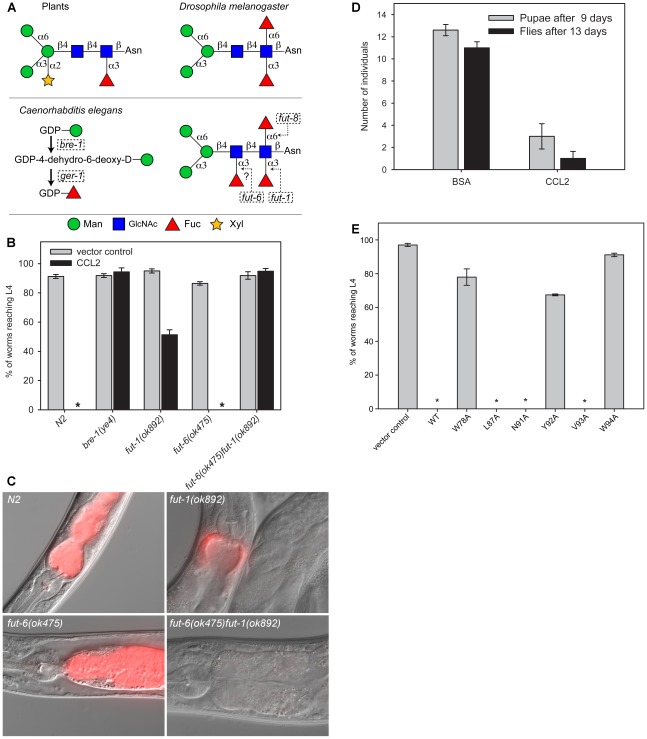
Carbohydrate-binding dependent biotoxicity of CCL2. (A) Schematic representation of N-glycan structures in plants, insects and nematodes. Upper panel, left: Typical paucimannosidic plant N-glycan, highly abundant in HRP. Upper panel right: Fucosylated paucimannosidic N-glycan present in *D. melanogaster*. Lower panel: Fucose biosynthesis and N-glycan structure in *C. elegans*. Genes coding for enzymes involved in the fucose biosynthesis (lower panel, left) and fucose transfer to the core of N-glycans in *C. elegans* (lower panel, right) are indicated in dashed boxes. (B) Toxicity of recombinant *E. coli* expressing CCL2 (black bars) towards *C. elegans* wildtype (N2) and various fucosylation mutants. Error bars indicate standard errors of the mean. Asterisks (*) show cases where all data were 0. Significant differences were observed between the vector control and CCL2 for N2 (n = 10, p = 0.013), *fut-1(ok892)* (n = 10, p = 0.013) and *fut-6(ok475)* (n = 10, p = 0.013) worms, but not for *bre-1(ye4)* (n = 10, p = 0.329) or *fut-6(ok475)fut-1(ok892)* (n = 10, p = 0.329). (C) Fluorescence microscopy of *C. elegans* feeding on *E. coli* expressing a dTomato-CCL2 fusion protein, showing the grinder and anterior part of the intestine. (D) Toxicity of purified CCL2 towards *D. melanogaster* quantified as number of developed pupae (gray bars) or flies (black bars). BSA was included as control. Error bars indicate standard errors of the mean. Development of pupae and flies treated with CCL2 were significantly different from the control (pupae: n = 10, p = 0.013; flies: n = 10, p = 0.013). (E) Toxicity of *E. coli* expressing different CCL2 variants with mutations in residues involved in carbohydrate binding towards *C. elegans* wildtype (N2). Vector control and CCL2 wildtype (WT) were included as controls. Asterisks (*) show cases where all data were 0. Error bars indicate standard error of the mean. W78A, Y92A and W94A were significantly different from WT control (n = 10, p = 0.013), whereas L87A, N91A, V93A were not (n = 10, p = 1.0).

These experiments showed a toxicity of CCL2 for *C. elegans* and *D. melanogaster* (Figure 8) but not for *A. aegypti* or *A. castellanii* (Figure S8). In the case of *C. elegans*, feeding on CCL2-expressing *E. coli* stopped the development of all wildtype (N2) L1 larvae in the assay (Figure 8B). This toxicity was dose-dependent and the presence of 30% of CCL2-expressing *E. coli* among the fed bacteria was sufficient to reduce the development of more than 95% of the L1 larvae (Figure S9). In the case of *D. melanogaster*, CCL2 caused a significant delay in development of both pupae and flies by 4- and 10-fold, respectively, relative to the control (Figure 8D). The toxicity of CCL1 towards *C. elegans* (Figure S10) was found to be similar to that of CCL2.

The observed toxicity was likely to be mediated by binding of CCL2 to the N-glycan cores of glycoproteins in the susceptible organisms since α1,3-fucosylation of N-glycan cores was demonstrated both for *C. elegans* and *D. melanogaster* and caused cross-reactivity of anti-HRP antisera with these organisms [31]. Therefore, *C. elegans* mutants impaired in either fucose biosynthesis (*bre-1*) or a variety of fucosyltransferases were tested for their susceptibility to CCL2-mediated toxicity (see scheme in Figures 8A and B; Figure S11). In agreement with our predictions, the *bre-1(ye4)* mutant that is unable to synthesize GDP-fucose was completely resistant to CCL2 intoxication. In addition, the *fut-1(ok892)* mutant lacking Fucα1,3 at the proximal GlcNAc of the chitobiose core [32] was partially resistant, as most of the worms survived and developed, but just half of the larvae reached L4 stage after 48 hours. On the other hand, a deletion in the *fut-6* gene, which results in loss of tetrafucosylated N-glycans in *C. elegans*, as does a deletion in the *fut-1* gene [32], was as sensitive as N2 (wildtype) to CCL2. In order to further explore these results, a *fut-6(ok475)fut-1(ok892)* double mutant was constructed and found to be completely resistant to CCL2 (Figure 8B). As nematodes are able to α1,3-fucosylate both GlcNAc residues of the core region of some N-glycans [33] and both *fut-1* and *fut-6* are required for the full fucosylation of this core region (see scheme in Figure 8A; Yan, Paschinger and Wilson, personal communication), our results suggest that the α1,3-fucosylated chitobiose core of N-glycans is the ligand of CCL2 in *C. elegans*. The partial resistance of the *fut-1* mutant can be explained by binding of CCL1/2 to N-glycan cores carrying a single fucose on the distal GlcNAc (Manβ1,4[Fucα1,3]GlcNAc). We hypothesize that this is a less favorable ligand due to the lack of an acetamido group on the mannose.

To study the phenotype of CCL2-mediated intoxication and to follow the fate of the toxic lectin in the worms, different *C. elegans* strains were fed with *E. coli* cells producing an N-terminal fusion of CCL2 to the red fluorescent dTomato protein [34]. As can be observed in Figure 8C, a strong fluorescence was observed in the upper intestine of the completely susceptible worms N2 and *fut-6(ok475)* as a result of CCL2-binding to the intestinal epithelium. This fluorescence was accompanied by an evident damage of intestinal cells which resulted in a massive expansion of the intestinal lumen. In agreement with the effects on larval development (Figure 8B), the *fut-*6(ok475)*fut-1(ok892)* double mutant that is resistant to CCL2-mediated intoxication, showed neither red fluorescence nor cell damage or expansion of the intestinal lumen. These results suggest that, in the absence of binding to the intestinal epithelium, the ingested lectin is completely cleared from the lumen after 1 hour. Accordingly, an intermediate phenotype, with some staining and cell damage, mostly in the upper part of the intestinal epithelium, was observed in the partially resistant *fut-1(ok892)* mutant.

### Effect of point mutations on carbohydrate-binding affinity and toxicity

We evaluated the contribution of individual amino acid side chains on the carbohydrate-binding affinity by introducing several point mutations at the binding interface followed by ITC measurements. All variants expressed well (except N91A) and folded properly as judged from ^15^N-HSQC spectra (Figure S12). Significant decreases in affinity were observed for all mutants except N91A (Table 1 and Figure S13). The Y92A mutation decreased the affinity beyond the detection limit. The second largest affinity decreases are observed for W94A and W78A, indicating that the aromatic side chains provide the largest contribution to carbohydrate-binding affinity. A significant decrease in affinity was also observed for Y57A, L87A, N90A and V93A point mutants (4- to 17-fold).

CCL2 variants were also tested *in vivo* for toxicity towards *C. elegans*. Remarkably, those mutants that retained carbohydrate binding with high affinity (K_D_<30 µM) *in vitro* were as toxic as wild type CCL2. Mutants with lower *in vitro* affinity, however, showed a decreased toxicity towards *C. elegans* (Figure 8E). In summary, these results confirm the carbohydrate-coordinating residues of CCL2 that were identified by NMR spectroscopy and suggest that high carbohydrate-binding affinity of the lectin is required for toxicity.

## Discussion

Our results strongly suggest that the newly identified lectins play a role in fungal defence. The lack of motility and the resulting inability of multicellular fungi and plants to escape from predators and parasites has led to the development of very similar defence strategies. In the absence of adaptive immune mechanisms and circulating immune cells, both types of organisms solely rely on innate defence. Whereas plant defence has already been intensively studied [9], [35]–[37], fungal defence has only recently been explored. It appears that, similar to plants, in addition to small molecules [38], proteins play a key role in the defence of multicellular fungi, in particular against predators and parasites [39]. Among the different types of potential fungal defence proteins identified [13], [26], [40], [41] the number and diversity of lectins is remarkably high, in accordance with the suitability of glycoepitopes for non-self recognition in innate defence mechanisms. Most fungal defence lectins are highly abundant in reproductive and long-term survival structures such as fruiting bodies and sclerotia, respectively, which require special protection [13]. This expression pattern, also found for CCL1 and CCL2 (Figures 1B–C), is analogous to plants where the expression of many lectins is confined to seeds.

The strong and specific toxicity of CCL1 and CCL2 towards *D. melanogaster* and C. *elegans* is in accordance with the prevalence of the phyla Arthropoda and Nematoda as predators of mushrooms both in nature [42], [43] and in mushroom farms [44], [45]. In addition, this specificity of CCL1/CCL2-mediated toxicity correlates with the identification of α1,3 fucosylated N-glycan cores as target structures of these lectins *in vivo* (Figure 8), since this epitope is present exclusively in plant and invertebrate N-glycans [31]. The NMR structure revealed that CCL2 recognizes the fucose-containing trisaccharide, GlcNAcβ1,4[Fucα1,3]GlcNAc, as part of this epitope with high specificity. Within this trisaccharide, almost all functional groups of Fucα1,3GlcNAc and the acetamido group of the distal GlcNAc2 are recognized. The recognition of the distal saccharide is more relaxed, a GalNAc with an acetamido at the same position will be equally well recognized. Accordingly, among the glycans of the mammalian glycan array, GalNAcβ1,4[Fucα1,3]GlcNAc (fucosylated LacdiNAc = LDN-F) was one of the best binders. Since there is space for extensions at O6 of GlcNAc1 and O4 of GlcNAc2 (Figure 5E) we can derive the following recognition sequence: X-1,4GalNAc/GlcNAcβ1,4[Fucα1,3][Y-1,6]GlcNAc in which X and Y are tolerated extensions. In addition, binding of substituted Lewis^X^ structures on the glycan array (Figure 2) suggests that substitutions at O3 and O6 of the galactose (corresponding to the distal GlcNAc in α1,3 fucosylated chitobiose) and at O6 of GlcNAc (corresponding to the proximal GlcNAc in α1,3 fucosylated chitobiose) are allowed. Accordingly, we would expect specific binding of CCL2 to paucimannose-type N-glycans carrying both α1,6 and α1,3-linked fucose on the proximal and possibly α1,3-linked fucose on the distal GlcNAc (Figure 8A). The GlcNAcβ1,4[Fucα1,3]GlcNAc motif is also a central part of the anti-HRP epitope that it is recognized by antisera raised against HRP in agreement with the isolation of CCL2 as HRP-binding lectin (Figure 1A). Since this epitope is also a key carbohydrate determinant of pollen and insect venom allergens [46], it appears that the same glycoepitope has been selected as target by the antibody-mediated mammalian adaptive immune system and a lectin-mediated fungal defence system.

The high affinity of CCL2 to the recognized trisaccharide determined by ITC is remarkable. Typically, individual carbohydrate binding sites of lectins have a rather low affinity to their ligands and this low affinity is usually compensated by multivalency achieved either by multiple binding sites on the same polypeptide chain or by oligomerization of polypeptide chains with one or few binding sites which leads to a high avidity towards multivalent ligands [47]. However, high affinity carbohydrate binding sites of lectins have been described and they differ from low affinity binding sites by their degree of specificity [48]: whereas low affinity binding sites often have a broad specificity towards terminal mono- or disaccharides present on many different glycans, high affinity sites recognize distinct oligosaccharides that are characteristic for specific glycans and glycoconjugates. The high affinity and specificity of the carbohydrate binding site in CCL2 towards the recognized trisaccharide is achieved by H-bonds and key hydrophobic contacts to almost all functional groups of Fucα1,3GlcNAc as well as the acetamido group of the distal GlcNAc2. The ladder interaction is central for the high affinity, the absence of the distal acetamido group as in Galβ1,4[Fucα1,3]GlcNAc (Lewis^X^) leads to a drop in affinity by ∼300 fold (in Table 1). To our knowledge, CCL2 is the only lectin that binds GlcNAcβ1,4[Fucα1,3]GlcNAc with such a high specificity and affinity, making CCL2 superior to anti-HRP for detection of this glycoepitope. Since this and the other recognized glycoepitope, GalNAcβ1,4[Fucα1,3]GlcNAc (LDN-F), are present in parasitic helminths [49]–[51], CCL2 may be used for the diagnostics of parasitic infections in animals and humans. The toxicity of CCL2-binding to at least one of these epitopes *in vivo*, may be exploited to develop novel approaches for the prevention or therapy of these infections. Another application could be the use of CCL2 on lectin microarrays for differential glycan profiling [52] or cellular glycomics [53].

The NMR solution structure of CCL2 in complex with its ligand demonstrates the versatility and plasticity of the β-trefoil fold with regard to carbohydrate binding. First, the carbohydrate specificity of CCL2 is very different from other β-trefoil lectins which recognize terminal galactose epitopes like Galα1,3Gal [19], Galβ1,3GalNAc [54] or Galβ1,3GlcNAc [21], rather than an epitope with a terminal fucose. Second, unlike most β-trefoil lectins which utilize three almost identical binding sites per monomer, CCL2 recognizes the identified carbohydrate ligand via a single binding site. This binding site of CCL2 is located at a very unusual site of the β-trefoil fold, the interface between subdomains β and γ. This stands in contrast to the fungal β-trefoil lectin SSA that also uses a single but canonical binding site [54]. None of the typical carbohydrate binding residues present in other β-trefoil lectins are found in CCL2 emphasizing the uniqueness of this non canonical binding site (Figure 6). Based on few β-trefoil complexes in which the binding site is slightly shifted from the canonical towards the CCL2 location [55]–[58] we speculate that this non-canonical binding site might have arisen from a previous recognition of other parts of the invertebrate N-glycan by the canonical binding site β (Figure 5G) and then have changed to recognize another epitope of the same glycan by the non-canonical binding site. The key residues of the CCL2 binding site are highly conserved in CCL2 homologs of other fungi (Figure 6 and Table S1), but highly variable in other β-trefoil lectins. The unusual carbohydrate specificity is mainly based on H-bonds from the protein main chain which requires the proper arrangement of three main chain sections: most importantly the characteristically short β7–β8 loop, strand β6 and the β9–β10 loop. In particular, the short β7–β8 loop is conserved in all CCL2 homologues with a consensus sequence LPxxYVW, a signature we propose for the identification of lectins with a similar target specificity. In summary, based on sequence alignment we predict that the homologous CCL2 like genes of basidiomycetes have the same unusual binding location and the same target specificity as CCL2 (except LB_L2 that lacks the crucial Y93). As we do not have any evidence for a difference in regulation, specificity or function between the different paralogs, e.g. CCL1 and CCL2, we speculate that this redundancy is a strategy to avoid loss of specific defense effectors by individual gene mutations.

The strong toxicity of CCL2 towards *C. elegans* and *D. melanogaster* is surprising in the light of the monomeric state of the lectin in solution and the consequential lack of multivalency for the identified ligand since clustering of glycoconjugates on cell surfaces is generally regarded as a prerequisite for lectin-mediated toxicity [59]. CCL2 mutant proteins unable to bind the HRP epitope are not able to bind anymore to the *C. elegans* intestinal epithelium which rules out the presence of an additional binding site on CCL2 with different specificity for this tissue (A. Butschi, unpublished results). Thus, we hypothesize that the high affinity of the single carbohydrate-binding site of CCL2 compensates for the lack of multivalency and that CCL2 acts by a novel toxicity mechanism that does not seem to involve clustering. Accordingly, CCL2 variants with a lower affinity *in vitro* showed a reduced toxicity in *C. elegans*. Remarkably, the consequences of intoxication of *C. elegans* by CCL1/2 and the multivalent fruiting body lectins MOA and CGL2 are very similar, all of them leading to disintegration of the intestinal epithelium and a substantial enlargement of the intestinal lumen (Figure 8C) [10], [16]. In addition, experiments aiming at the localization of the target glycoconjugates using fluorescently labeled CCL2 and CGL2 gave very similar results (Figure 8C) [16]. Interestingly, disintegration of the intestinal epithelium and enlargement of the intestinal lumen were also observed with the nematode-specific Cry toxins from *Bacillus thuringiensis* where carbohydrate-dependent binding to the intestinal epithelium appears to trigger expulsion of microvilli from the apical side of the intestinal epithelial cells [60]. In any case, interference with carbohydrate binding by the lectin, either by mutating genes involved in the biosynthesis of the identified target glycans in *C. elegans* or altering the identified carbohydrate binding sites in the lectin, abolished toxicity and binding of the fluorescently labeled lectin to the intestinal epithelium (Figure 8C) [10], [16]. It should be noted, however, that not all variants of CCL2 were tested for toxicity towards *C. elegans* and none was tested for toxicity towards *D. melanogaster*. Thus, although we can show that the recognition of specific glycans is a crucial part of lectin-mediated defence mechanisms, the exact mechanisms of toxicity remain to be elucidated. Possible mechanisms are direct membrane damage or the interference with cellular signaling pathways, recycling of cell surface receptors, cell-cell or cell-matrix interactions. In order to distinguish between these possibilities and to find potential targets of novel antihelminthics, we are currently in the process of identifying the glycoprotein(s) targeted by CCL2 and CGL2 in *C. elegans*.

## Materials and Methods

### Carbohydrates

Lewis^X^ trisaccharide methyl glycoside, 3′-Sialyl-Lewis^X^ tetrasaccharide methyl glycoside and Fucα1,3GlcNAc-OMe were purchased from Carbosynth, UK. The chemically synthesized fucosylated chitobiose GlcNAcβ1,4[Fucα1,3]GlcNAcβ-O(CH_2_)_5_COONa [61] was a kind gift of Mayeul Collot, ENS, France. Lewis^X^ tetrasaccharide and 3′-Sialyl-lactose were a kind gift of Eric Samain, CERMAV, France. The identity and purity of the carbohydrates was checked using 2D NMR spectroscopy.

### Strains and cultivation conditions

Detailed information of the strains used in this study can be found in Table S7. *Escherichia coli* strain DH5α was used for cloning and amplification of plasmids, strains BL21(DE3) and BL21(DE3)/pLysS were used for bacterial expression of proteins and biotoxicity assays and strain OP50 was used to feed *C. elegans* during regular breeding. Cultivation conditions of the various organisms are described in Text S1.

### Isolation and purification of CCL2 from *C. cinerea*

CCL2 was isolated and purified from *C. cinerea* as described in Text S1.

### Identification of CCL2 by peptide mass fingerprinting

Purified CCL2 was separated by SDS-PAGE, excised from the gel and identified by MALDI-MS/MS. Details of the procedure are described in Text S1.

### Quantification of *ccl1* and *ccl2* expression by qRT-PCR

Details of the quantification are described in Text S1.

### Cloning of CCL1- and CCL2-encoding genes

The PCR-based cloning strategies for the various CCL1- and CCL2-encoding genes are described in Text S1.

### Determination of CCL1 and CCL2 expression levels in *C. cinerea*

Protein expression of CCL2 was evaluated by immunoblotting. Soluble protein extracts of vegetative mycelium and fruiting bodies from *C. cinerea* were obtained as described above and separated on a 12% SDS-PAGE and probed with specific antiserum raised in rabbits against purified recombinant CCL2 (Pineda Antikörper-Sevice, Berlin, Germany) and detected with HRP-conjugated secondary antibodies. Transcription levels of both genes were assessed by quantitative real-time PCR (qRT-PCR) as described in Text S1.

### Glycan array analysis of CCL1 and CCL2

Purified CCL1 and CCL2 were fluorescently labeled with Alexa Fluor 488 (Invitrogen) according to the manufacturer's protocol and used (at a final concentration of 200 µg/ml) to probe versions 4.2 and 3.1, respectively, of the mammalian glycan array offered by Core H of the Consortium for Functional Glycomics (CFG).

### Preparation of proteins and their carbohydrate complexes

Unlabelled and uniformly ^15^N or ^13^C/^15^N labeled proteins were overexpressed in *E. coli* as His8-fusions and purified with affinity chromatography (see Text S1). Samples were dialyzed against NMR buffer (50 mM KH_2_PO_4_, pH 5.7, 150 mM NaCl). Complexes of CCL2 with GlcNAcβ1,4[Fucα1,3]GlcNAcβ-O(CH_2_)_5_COONa were prepared by titrating the concentrated carbohydrate solution of typically 10 mM into a ∼1 mM solution of CCL2 in NMR buffer until a 1∶1 stoichiometry was reached. Subsequently, the pH was lowered to 4.7 using 10% deuterated acetic acid to avoid precipitation.

### NMR spectroscopy

NMR spectra were acquired on Avance III 500, 600, 700, 750 and Avance 900 Bruker spectrometers at 310 K. NMR data were processed using Topspin 2.1 (Bruker) and analyzed with Sparky (Goddard, T.D. & Kneller, D.G. SPARKY 3. University of California, San Francisco). The ^1^H,^13^C,^15^N chemical shifts of the protein, free and in complex, were assigned by standard methods [62]. Assignment of carbohydrate resonances of the complex was achieved using NOE correlations and exchange peaks with signals of the free carbohydrate since neither TOCSY based spectra nor a natural abundance ^13^C-HSQC showed bound carbohydrate signals. The following spectra were used for this purpose 2D ^1^H-^1^H NOESY, 2D ^13^C/^15^N F1-filtered NOESY and 2D ^13^C F1-filtered F2-filtered NOESY [63]. The assignments of intermolecular NOEs were derived from 3D ^13^C F1-edited, F3-filtered NOESY-HSQC [27] spectra of the protein-carbohydrate complex. More details are found in the Text S1.

### Structure calculation and refinement

The AtnosCandid software package [64], [65] was used to generate initial CCL2 structures (free and bound) using three 3D NOESY spectra (^13^C^ali^-edited, ^13^C^aro^-edited and ^15^N-edited) and one 2D NOESY spectrum. The automatically generated upper limit restraints file was used as a starting point for the first level of manually refining the protein structures by a simulated annealing protocol using the Cyana package [64]. Preliminary structures of the CCL2-carbohydrate complex were generated using the Cyana package with the above mentioned restraints and manually assigned intermolecular and intra-carbohydrate NOE distance constraints. To create the topology of the carbohydrate for the Cyana library file an initial model was generated by SWEET [66]. The carbohydrate spacer was truncated to a methyl group. 300 structures were generated by CYANA starting from random carbohydrate and protein starting structures. Ensemble of 30 structures of CCL2 free and in complex were refined with AMBER 9.0 [67].in implicit solvent using NOE-derived distances, torsion angles and hydrogen bond restraints as summarized in Table 2. For more details see Text S1. The Ramachandran statistics of CCL2 free and in complex, respectively, show 79.9% and 80.2% in the most favored regions, 18.0% and 18.7% in the additionally allowed regions, 1.5% and 1.0% in the generously allowed regions and 0.6% and 0.2% in the disallowed regions.

### Biotoxicity assays with recombinant CCL2

Biotoxicity assays for *A. aegypti* and *A. castellanii* were performed with recombinant *E. coli* as previously described [30]. For *C. elegans*, a liquid toxicity assay was performed as follows: a synchronous population of L1 larvae as well as a bacterial culture of recombinant *E. coli* expressing CCL2 or containing a vector control were obtained as described [22]. *E. coli* cells were pelleted and re-suspended in sterile PBS to an OD_600_ = 2. The assay was set up in 96-well plates (TPP) by mixing 80 µl of the bacterial suspension and 20 µl of L1 larvae containing approximately 30 individuals. Each treatment (different bacterial and/or worm strain combinations) was done in 5 replicates. The worms were allowed to feed on the suspended bacteria at 20°C in the dark. The total number of animals and the percentage of individuals reaching L4 stage were quantified after 48 h. The biotoxicity assay with *D. melanogaster* was performed adding purified protein to the rearing medium as previously described [68] using 20 eggs.

For the statistical analysis of the toxicity assays, pairwise comparisons were done using the non-parametric Kolmogorov-Smirnov test in the case of *C. elegans*, *A. castellanii* and *D. melanogaster* and the parametric T-student test for *A. castellanii*. The response variables (development, survival and clearing area) were compared between the tested lectin and the control or between mutant and wildtype.

### Preparation of the *C. elegans fut-1 fut-6* double mutant (F1F6) and PCR screening

Details are described in Text S1.

### Localization of CCL2-binding in *C. elegans*

More information is found in Text S1.

### Isothermal titration calorimetry (ITC)

ITC experiments were performed on a VP-ITC instrument (MicroCal). The calorimeter was calibrated according to the manufacturer's instructions. Protein and carbohydrate samples were dialyzed against NMR buffer at room temperature using a 3.5 kDa membrane (Spectra/Por) and Micro DispoDialyzer (100 Da cutoff; Harvard Apparatus), respectively. The disaccharide Fucα1,3GlcNAc-OMe was not dialyzed but dissolved in NMR buffer. The sample cell (1.4 mL) was loaded with 70 µM protein; carbohydrate concentration in the syringe was 2–4 mM. A titration experiment typically consisted of 30–50 injections, each of 3 µL volume and 6 s duration, with a 6.7 min interval between additions. Stirring rate was 307 rpm. Raw data were integrated, corrected for nonspecific heats, normalized for the molar concentration, and analyzed according to a 1∶1 binding model.

### Accession codes and numbers

The atomic coordinates of the structures of CCL2 free and in complex with the fucosylated chitobiose (GlcNAcβ1,4[Fucα1,3]GlcNAcβ-OMe) have been deposited in the Protein Data Bank with accession codes 2LIE and 2LIQ, respectively. The chemical shifts of the free protein and in complex were deposited in the BioMagResBank (BMRB) under the accession numbers 17890 and 17902, respectively. The cDNA sequences of CCL1 and CCL2 from *C. cinerea* strain AmutBmut were deposited in GenBank under accession number ADO87036 and ACD88750, respectively.

## Supporting Information

Figure S1 **Coomassie-stained SDS-PAGE showing the expression and solubility of CCL1 and CCL2 recombinantly expressed in** ***E.coli*** **.** WCE: whole cell extracts; SE: soluble fraction of WCE.
(TIF)

Figure S2 **Size exclusion chromatography showing the monomeric state of CCL2 compared to standards proteins.** (A) Elution profile of standard proteins (dotted lines, numbers indicate size in kDa) and CCL2 (red) which elutes at 12.1 ml from a Superdex 75 10/300 (GL) column. (B) Calibration curve done with the following standards: Ovalbumin 44 kDa, Carbonic anhydrase (29 kDa), Myoglobin (17 kDa), RNAse A (13.7 kDa) and Vitamin B1_2_ (1.35 kDa). The void volume was determined by elution of bovine γ-globulin (8.04 ml). The calculated molecular weight for CCL2 (in red) is 17.6. Gel filtration was performed at a flow rate of 0.5 ml/min in 10 mM sodium phosphate, 150 mM NaCl buffer pH 6.2. Samples of 2–5 mg/ml of protein in 0.1 ml were injected, and the eluate was monitored at 280 nm.
(TIF)

Figure S3 **Glycan array analysis showing the carbohydrate-binding specificity of CCL1.** Results shown are averages of triplicate measurements of fluorescence intensity at a lectin concentration of 200 µg/ml probed on the Mammalian Glycan Array (V 4.2). Error bars indicate the standard deviations of the mean. Glycan structures are depicted for those epitopes with highest relative fluorescence. The raw data and the entire list of glycans with the respective spacers can be found on the CFG homepage [http://functionalglycomics.org/] or in Table S3. Binding of 6'sulfo-sialyllactose (glycan #45) is likely to be an artifact since it is also bound by fucose-binding lectin AAL [http://functionalglycomics.org/].
(TIF)

Figure S4 **Isothermal titration calorimetry binding experiments between wild type CCL2 and different carbohydrate ligands.** Raw calorimetric outputs are shown on the top and binding isotherms describing the complex formation are shown at the bottom. The protein concentration in the cell was 70 µM and the carbohydrate concentration was 3.0 mM.
(TIF)

Figure S5 **3D F1-edited F3-filtered HSQC-NOESY spectrum.** A) Carbohydrate resonances are well dispersed in the direct dimension ω_3_ (^13^C filtered/suppressed). Shown is a slice at the ω_2_ (^13^C edited/selected) resonance of V93 methyl group QG2 displaying intermolecular NOEs. B) Slice of the two indirect dimensions ω_1_ and ω_2_ at the ω_3_ resonance of Fucose H2_bound_ showing intermolecular NOEs to Fucose H2. The ^1^H–^13^C correlations of the ^13^C labelled protein were directly compared to the ^13^C HSQC spectrum of the protein to assign the intermolecular NOEs.
(TIF)

Figure S6 **The three symmetry-related canonical binding sites of β-trefoil proteins illustrated by the lectin MOA.** (A–C) Three different side views related to each other by rotation of 120° around the z axis of MOA in complex with Galα1,3(Fucα1,2)Gal (PDB: 3EF2). The binding sites are indicated by Greek letters. (D) Top view of the same complex. The same colors and similar orientations are used as for CCL2 in Figs. 4 and 5. (E) Superposition of the CCL2 complex structure (blue) with ligand (cyan) on the MOA complex structure 3EF2 (grey) with ligands (yellow). The β subunit is located in front.
(TIF)

Figure S7 **Angle plots of the glycosidic linkages of fucosylated chitobiose found in the 20 calculated complex structures.** The plots, generated by CARP [73], display the observed angles (red with labels in blue) on top of an energy landscape calculated by modelling (top) or on top of angles of the same disaccharide linkage found in all structures deposited in the PDB database (bottom).
(TIF)

Figure S8 **Toxicity of CCL2 towards** ***A. aegypti*** **and** ***A. castellanii*** **.** Toxicity of CCL2-expressing *E. coli* towards larvae of the mosquito *A. aegypti* (A) and the amoebozoon *A. castellanii* (B) was assessed as described in Materials and Methods. Error bars indicate standard errors of the mean. No significant differences were observed between CCL2 and VC (p>0.05).
(TIF)

Figure S9 **Dose-dependence of CCL2-mediated nematotoxicity.** Wildtype *C. elegans* (N2) were fed with mixtures of CCL2-expressing *E. coli* expressing CCL2 and empty vector-containing *E. coli*. Error bars indicate standard errors of the mean.
(TIF)

Figure S10 **Carbohydrate-binding dependence of CCL1-mediated nematotoxicity.** Toxicity of CCL1-expressing *E. coli* towards *C. elegans* wild type (N2) and various fucosylation mutants. Error bars indicate standard errors of the mean. Asterisks (*) show cases where all data were 0. Assays were done in solid media as described [30].
(TIF)

Figure S11 **Toxicity of recombinant** ***E. coli*** **expressing CCL2 towards** ***C. elegans*** **wildtype (N2) and various mutants in predicted or characterized fucosyltransferases (fut) or GlcNAc-transferases (gly).** Assays were done in solid media as described [30]. Error bars indicate standard errors of the mean. Asterisks (*) show cases where all data were 0. †: In the *fut-1(ok892)* mutants a partial resistance is observed. Although the larvae survive and develop, they require at least 24 h more to reach L4 and look thinner and paler than the complete resistant double mutant *fut-6(ok475)fut-1(ok892)*.
(TIF)

Figure S12 **All CCL2 proteins containing a point mutant are folded.** ^15^N-HSQC spectra of ^15^N labelled proteins.
(TIF)

Figure S13 **Isothermal titration calorimetry binding experiments between CCL2 mutants and fucosylated chitobiose (GlcNAcβ1,4[Fucα1,3]GlcNAcβ1-spacer).** Raw calorimetric outputs are shown on the top and binding isotherms describing the complex formation are shown at the bottom. The protein concentration in the cell was 70 µM and the carbohydrate concentration was 3.0 mM.
(TIF)

Table S1 **Sequence identities among the CCL2 homologues and the lectin MOA and SNA-II.**
(PDF)

Table S2 **Raw data of glycan array analysis performed with CCL2.** RFU = Relative Fluorescence Units; SD = Standard deviation.
(PDF)

Table S3 **Raw data of glycan array analysis performed with CCL1.** RFU = Relative Fluorescence Units; SD = Standard deviation.
(PDF)

Table S4 **Most prominent proteins structurally closest to CCL2 (free) obtained by a DALI search** [**74**] **.**
(PDF)

Table S5 **Carbohydrate ^1^H and ^13^C chemical shifts [ppm] at 293 K referenced to DSS according to** [**75**] **.** Bound chemical shifts were assigned via exchange peaks between the free and the bound form and by NOEs. Bound signals were not visible in a natural abundance ^13^C HSQC.
(PDF)

Table S6 **Thermodynamic data of selected lectin–carbohydrate interactions that are used in** **Fig. 7B** **.**
(PDF)

Table S7 **Strains used in this study.**
(PDF)

Table S8 **Oligonucleotides used in this study.** Restriction sites in the oligonucleotides are underlined, and codon changes for site directed mutagenesis are in bold.
(PDF)

Text S1 **Supplementary methods.**
(PDF)
