# Correction: Plasticity of the β-Trefoil Protein Fold in the Recognition and Control of Invertebrate Predators and Parasites by a Fungal Defence System

**DOI:** 10.1371/annotation/088ea07b-d578-4586-9707-160143d4f1be

**Published:** 2012-08-15

**Authors:** Mario Schubert, Silvia Bleuler-Martinez, Alex Butschi, Martin A. Wälti, Pascal Egloff, Katrin Stutz, Shi Yan, Mayeul Collot, Jean-Maurice Mallet, Iain B. H. Wilson, Michael O. Hengartner, Markus Aebi, Frédéric H.-T. Allain, Markus Künzler

Reference 61 should instead refer to Supporting File S1 entitled "Synthesis and characterisation of GlcNAcß1,4[Fuca1,3]GlcNAcß1-O-(CH2)5-COOH", which does not appear in the Supporting Files Legend. Please view Supporting File S1 here: 
