# Correction: Plasticity of the β-Trefoil Protein Fold in the Recognition and Control of Invertebrate Predators and Parasites by a Fungal Defence System

**DOI:** 10.1371/annotation/ed5d46f6-3442-4a89-a505-78befd507436

**Published:** 2012-08-15

**Authors:** Mario Schubert, Silvia Bleuler-Martinez, Alex Butschi, Martin A. Wälti, Pascal Egloff, Katrin Stutz, Shi Yan, Mayeul Collot, Jean-Maurice Mallet, Iain B. H. Wilson, Michael O. Hengartner, Markus Aebi, Frédéric H.-T. Allain, Markus Künzler

Mayeul Collot and Jean-Maurice Mallet were not included in the author byline. They should be listed as the eighth and ninth authors, respectively. Both Mayeul Collot and Jean-Maurice Mallet are affiliated with Ecole Normale Supérieure CNRS, Departement de Chimie, Paris, France. The contributions of Mayeul Collot are as follows: contributed reagents/materials/analysis tools and performed the experiments. The contributions of Jean-Maurice Mallet are as follows: conceived and designed the experiments.

