# Supplementary figures and images for: Plasticity of the β-Trefoil Protein Fold in the Recognition and Control of Invertebrate Predators and Parasites by a Fungal Defence System
Source: PLoS Pathog. 2012 May 17;8(5):e1002706. doi: 10.1371/journal.ppat.1002706 (PMC3355094; doi:10.1371/journal.ppat.1002706)

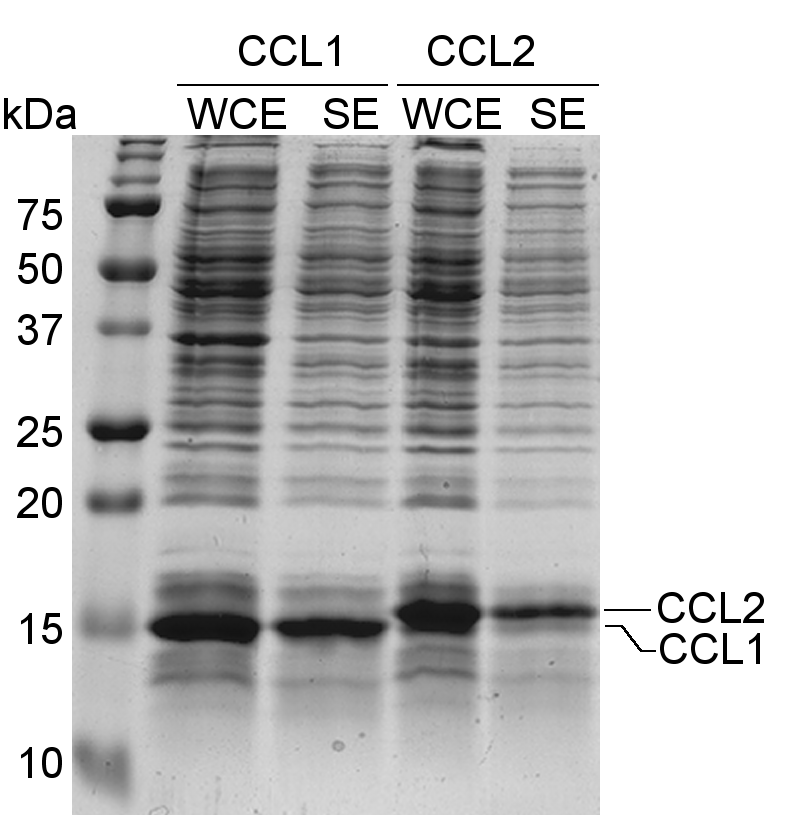

Supplement: Figure S1 — Coomassie-stained SDS-PAGE showing the expression and solubility of CCL1 and CCL2 recombinantly expressed in E.coli . WCE: whole cell extracts; SE: soluble fraction of WCE. (TIF) [file ppat.1002706.s001.tif]

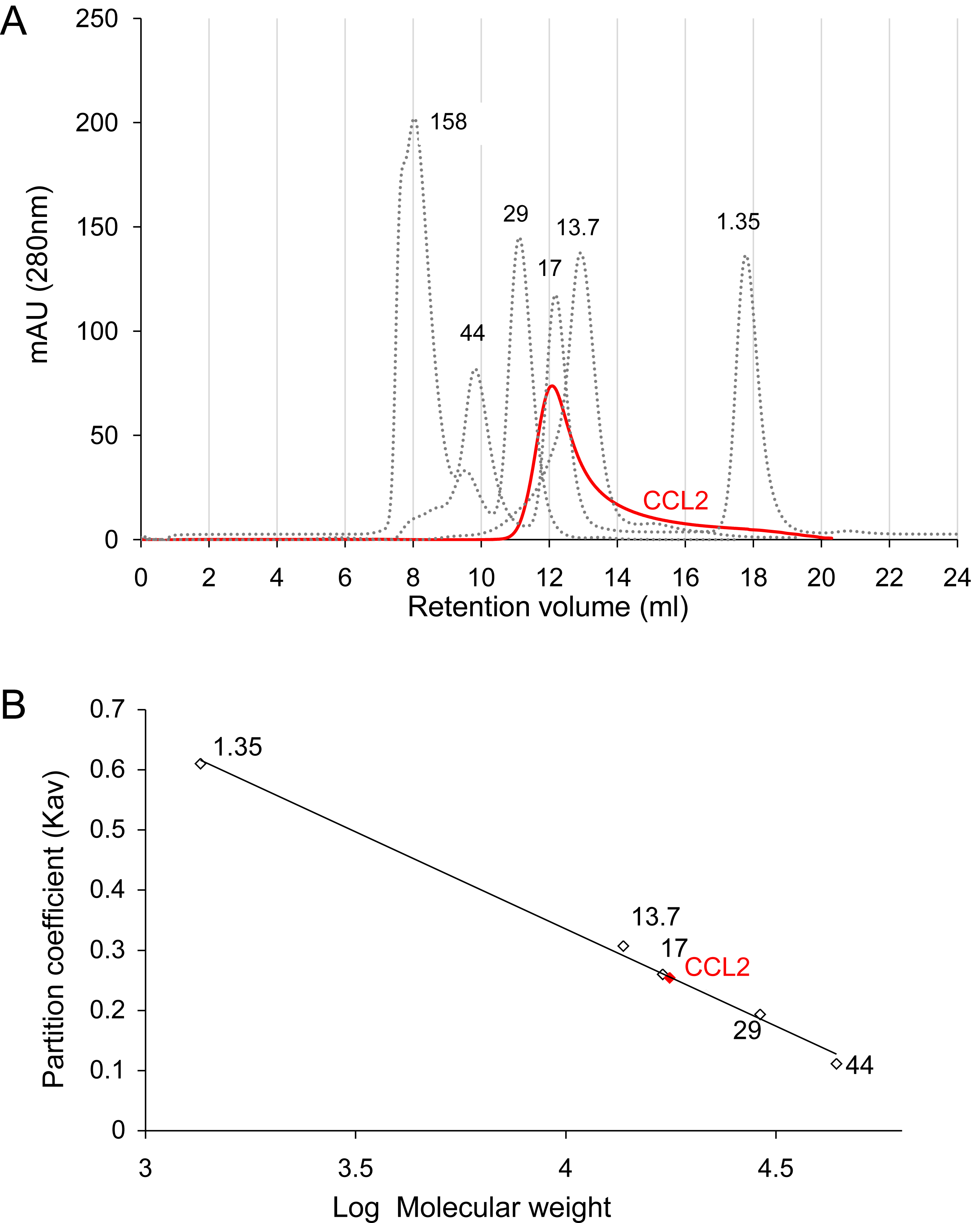

Supplement: Figure S2 — Size exclusion chromatography showing the monomeric state of CCL2 compared to standards proteins. (A) Elution profile of standard proteins (dotted lines, numbers indicate size in kDa) and CCL2 (red) which elutes at 12.1 ml from a Superdex 75 10/300 (GL) column. (B) Calibration curve done with the following standards: Ovalbumin 44 kDa, Carbonic anhydrase (29 kDa), Myoglobin (17 kDa), RNAse A (13.7 kDa) and Vitamin B12 (1.35 kDa). The void volume was determined by elution of bovine γ-globulin (8.04 ml). The calculated molecular weight for CCL2 (in red) is 17.6. Gel filtration was performed at a flow rate of 0.5 ml/min in 10 mM sodium phosphate, 150 mM NaCl buffer pH 6.2. Samples of 2–5 mg/ml of protein in 0.1 ml were injected, and the eluate was monitored at 280 nm. (TIF) [file ppat.1002706.s002.tif]

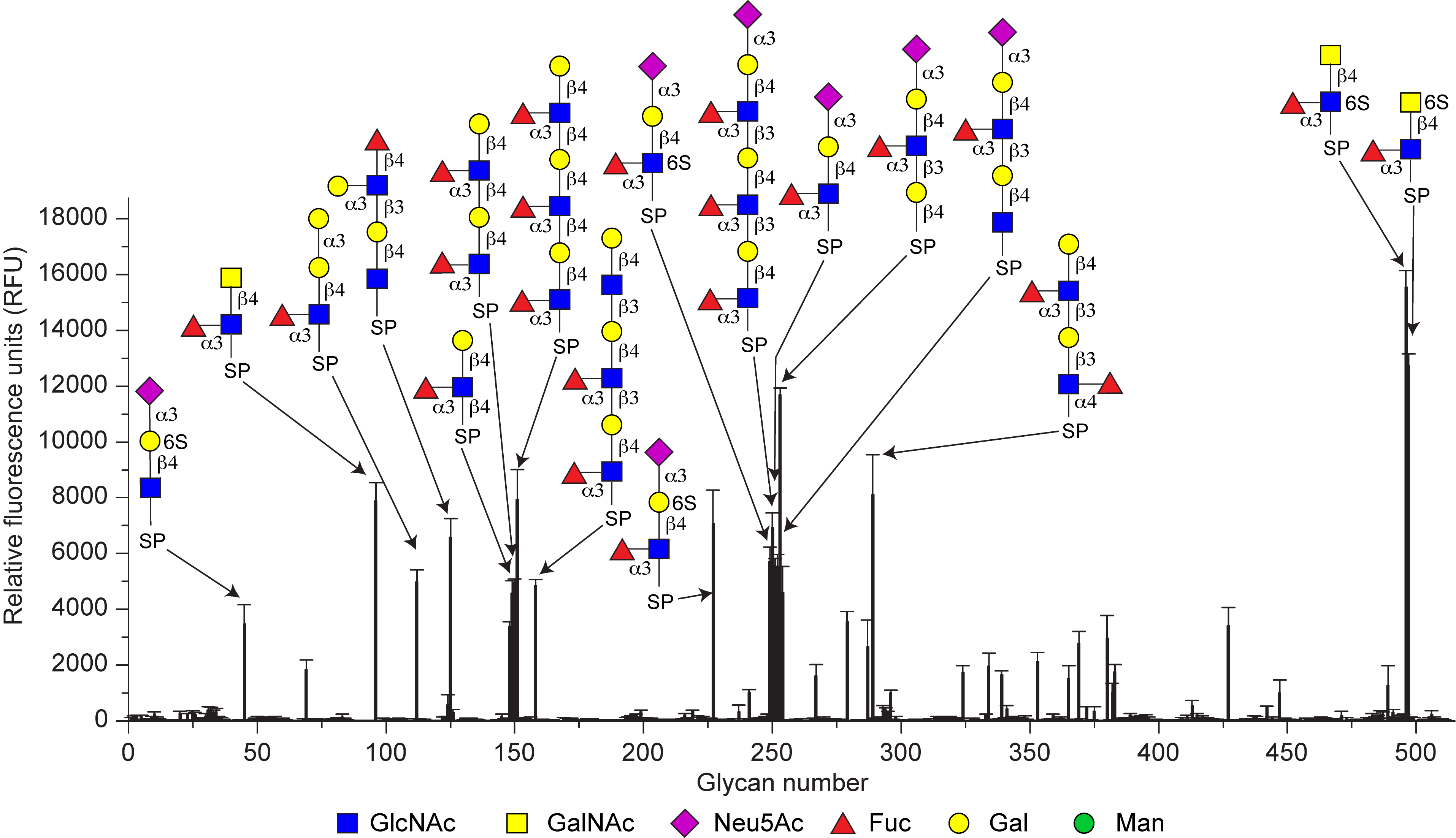

Supplement: Figure S3 — Glycan array analysis showing the carbohydrate-binding specificity of CCL1. Results shown are averages of triplicate measurements of fluorescence intensity at a lectin concentration of 200 µg/ml probed on the Mammalian Glycan Array (V 4.2). Error bars indicate the standard deviations of the mean. Glycan structures are depicted for those epitopes with highest relative fluorescence. The raw data and the entire list of glycans with the respective spacers can be found on the CFG homepage [http://functionalglycomics.org/] or in Table S3. Binding of 6'sulfo-sialyllactose (glycan #45) is likely to be an artifact since it is also bound by fucose-binding lectin AAL [http://functionalglycomics.org/]. (TIF) [file ppat.1002706.s003.tif]

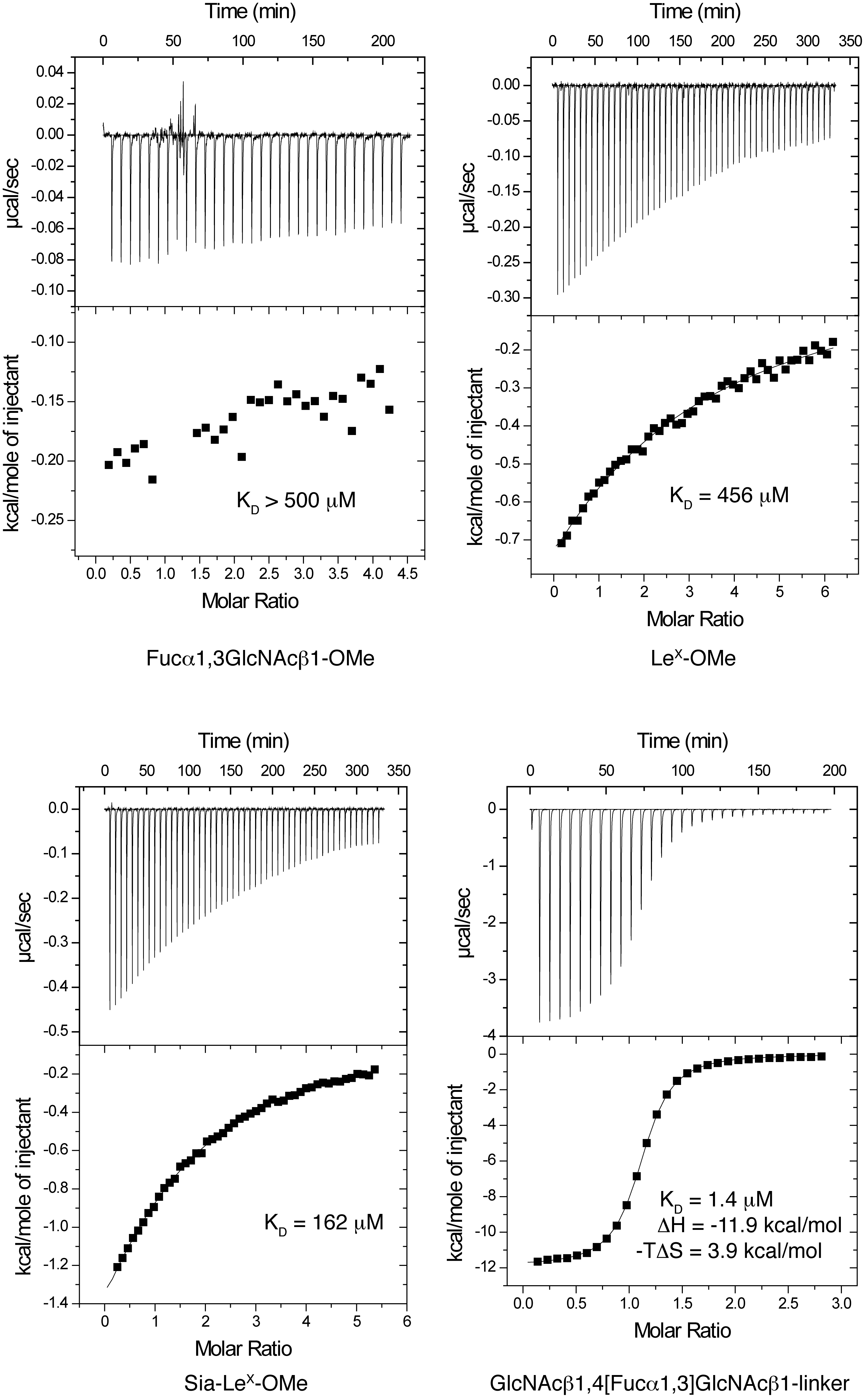

Supplement: Figure S4 — Isothermal titration calorimetry binding experiments between wild type CCL2 and different carbohydrate ligands. Raw calorimetric outputs are shown on the top and binding isotherms describing the complex formation are shown at the bottom. The protein concentration in the cell was 70 µM and the carbohydrate concentration was 3.0 mM. (TIF) [file ppat.1002706.s004.tif]

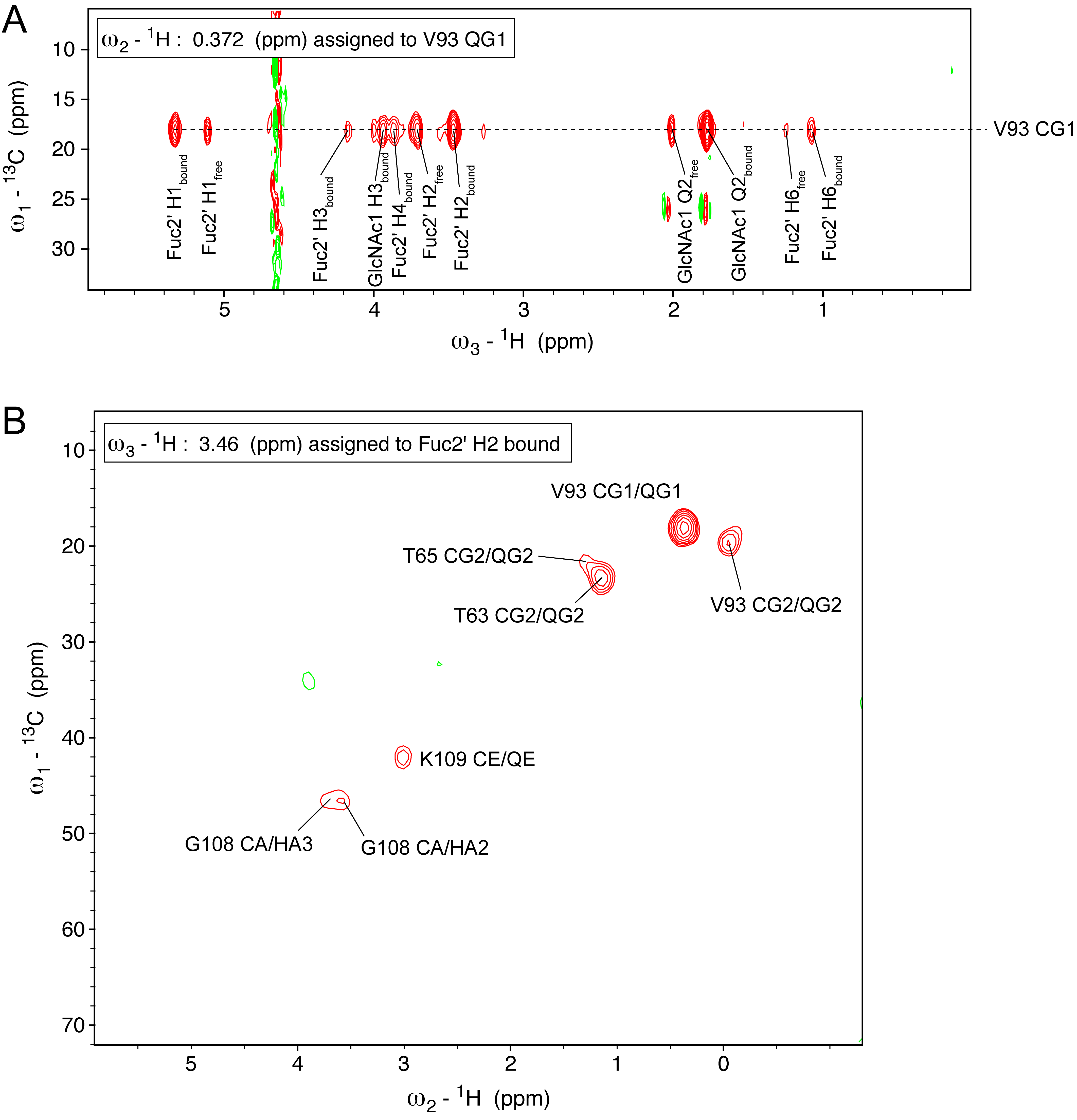

Supplement: Figure S5 — 3D F1-edited F3-filtered HSQC-NOESY spectrum. A) Carbohydrate resonances are well dispersed in the direct dimension ω3 (13C filtered/suppressed). Shown is a slice at the ω2 (13C edited/selected) resonance of V93 methyl group QG2 displaying intermolecular NOEs. B) Slice of the two indirect dimensions ω1 and ω2 at the ω3 resonance of Fucose H2bound showing intermolecular NOEs to Fucose H2. The 1H–13C correlations of the 13C labelled protein were directly compared to the 13C HSQC spectrum of the protein to assign the intermolecular NOEs. (TIF) [file ppat.1002706.s005.tif]

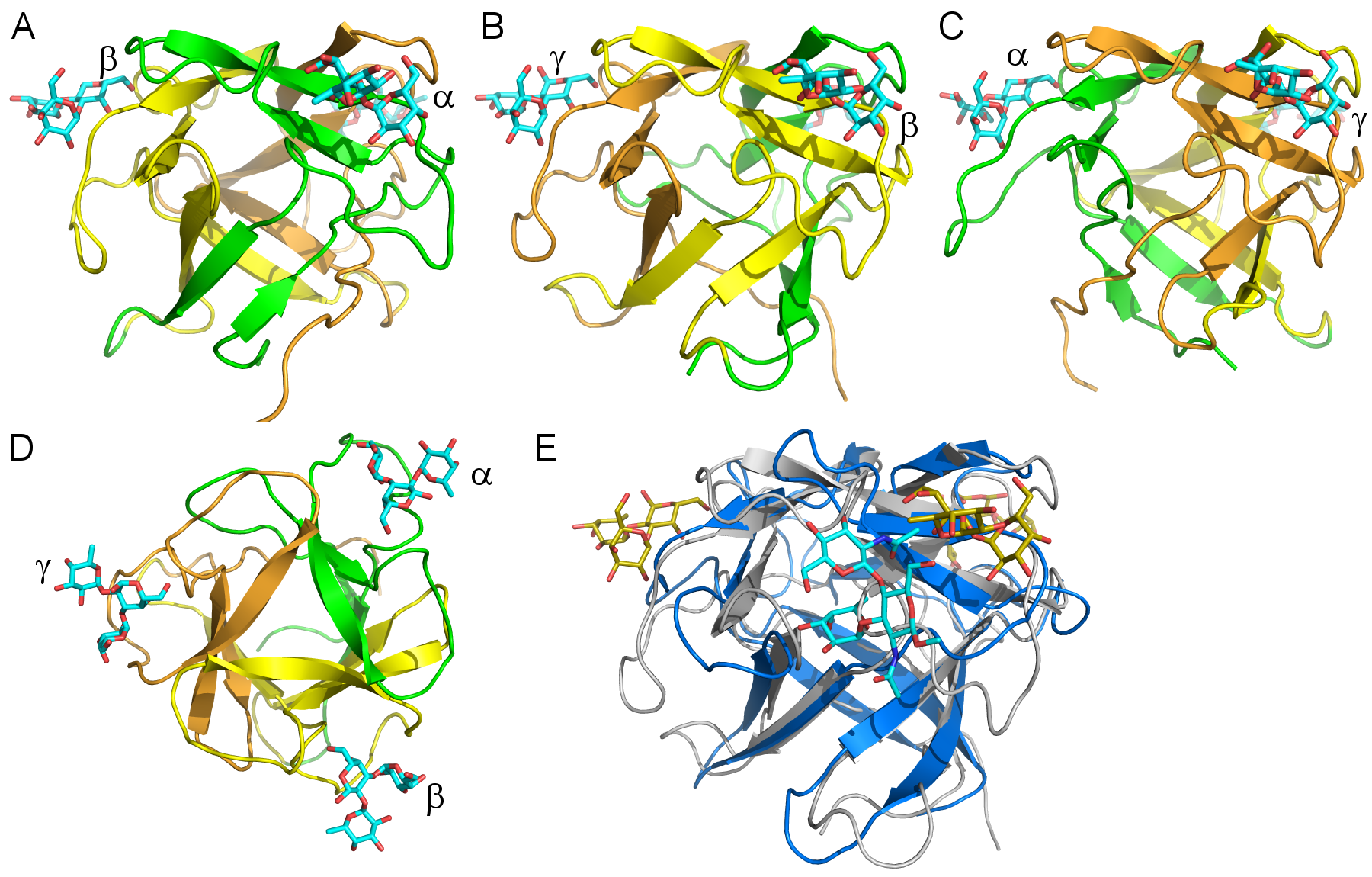

Supplement: Figure S6 — The three symmetry-related canonical binding sites of β-trefoil proteins illustrated by the lectin MOA. (A–C) Three different side views related to each other by rotation of 120° around the z axis of MOA in complex with Galα1,3(Fucα1,2)Gal (PDB: 3EF2). The binding sites are indicated by Greek letters. (D) Top view of the same complex. The same colors and similar orientations are used as for CCL2 in Figs. 4 and 5. (E) Superposition of the CCL2 complex structure (blue) with ligand (cyan) on the MOA complex structure 3EF2 (grey) with ligands (yellow). The β subunit is located in front. (TIF) [file ppat.1002706.s006.tif]

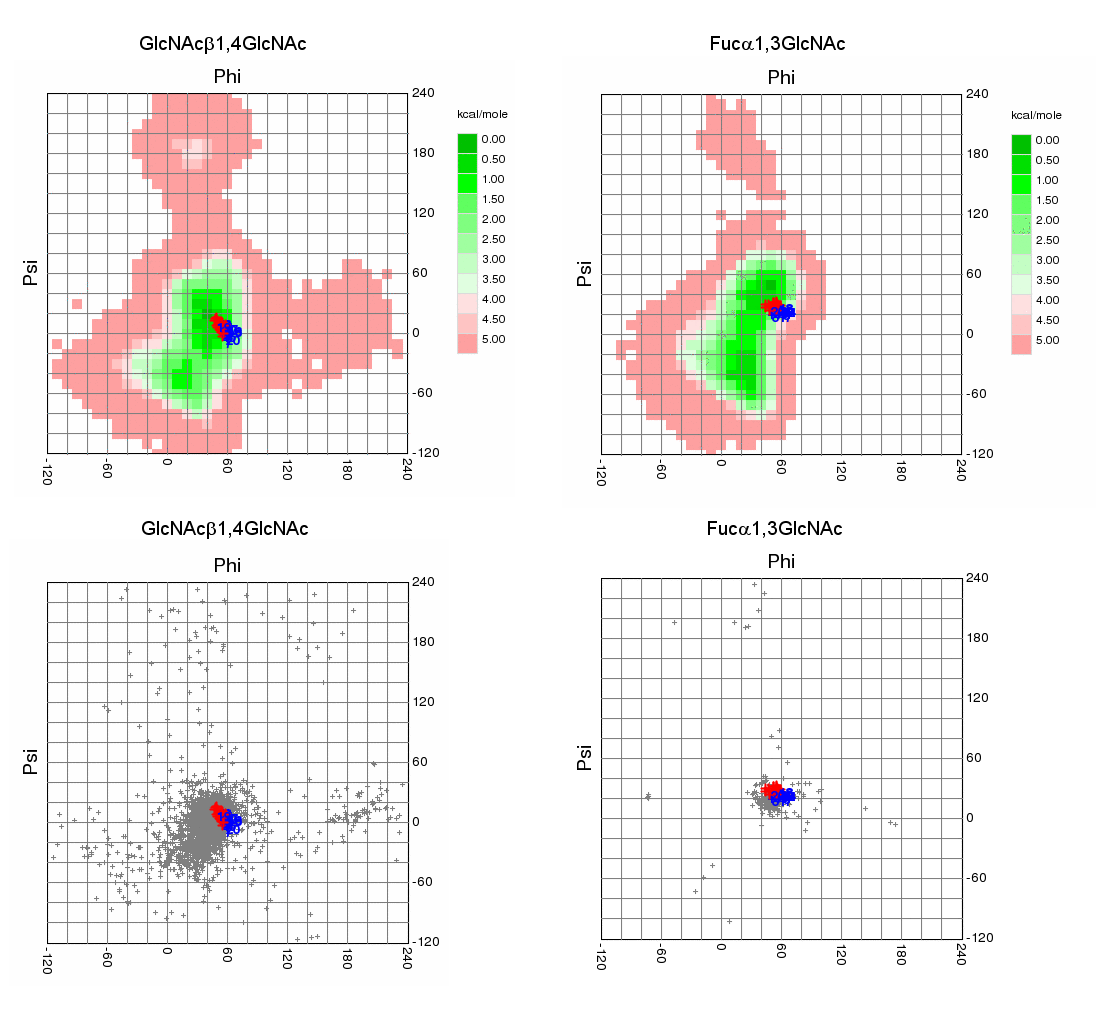

Supplement: Figure S7 — Angle plots of the glycosidic linkages of fucosylated chitobiose found in the 20 calculated complex structures. The plots, generated by CARP [73], display the observed angles (red with labels in blue) on top of an energy landscape calculated by modelling (top) or on top of angles of the same disaccharide linkage found in all structures deposited in the PDB database (bottom). (TIF) [file ppat.1002706.s007.tif]

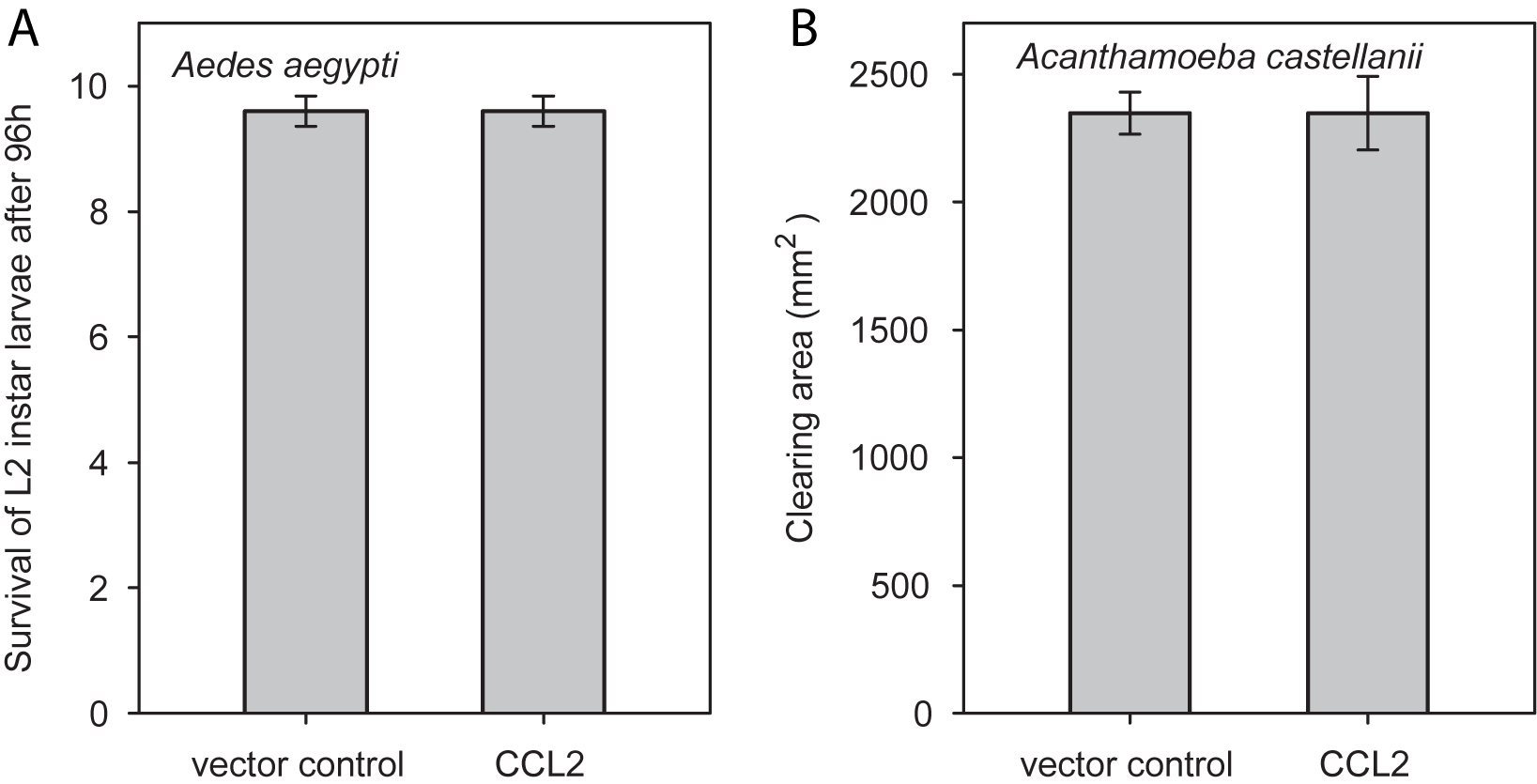

Supplement: Figure S8 — Toxicity of CCL2 towards A. aegypti and A. castellanii . Toxicity of CCL2-expressing E. coli towards larvae of the mosquito A. aegypti (A) and the amoebozoon A. castellanii (B) was assessed as described in Materials and Methods. Error bars indicate standard errors of the mean. No significant differences were observed between CCL2 and VC (p>0.05). (TIF) [file ppat.1002706.s008.tif]

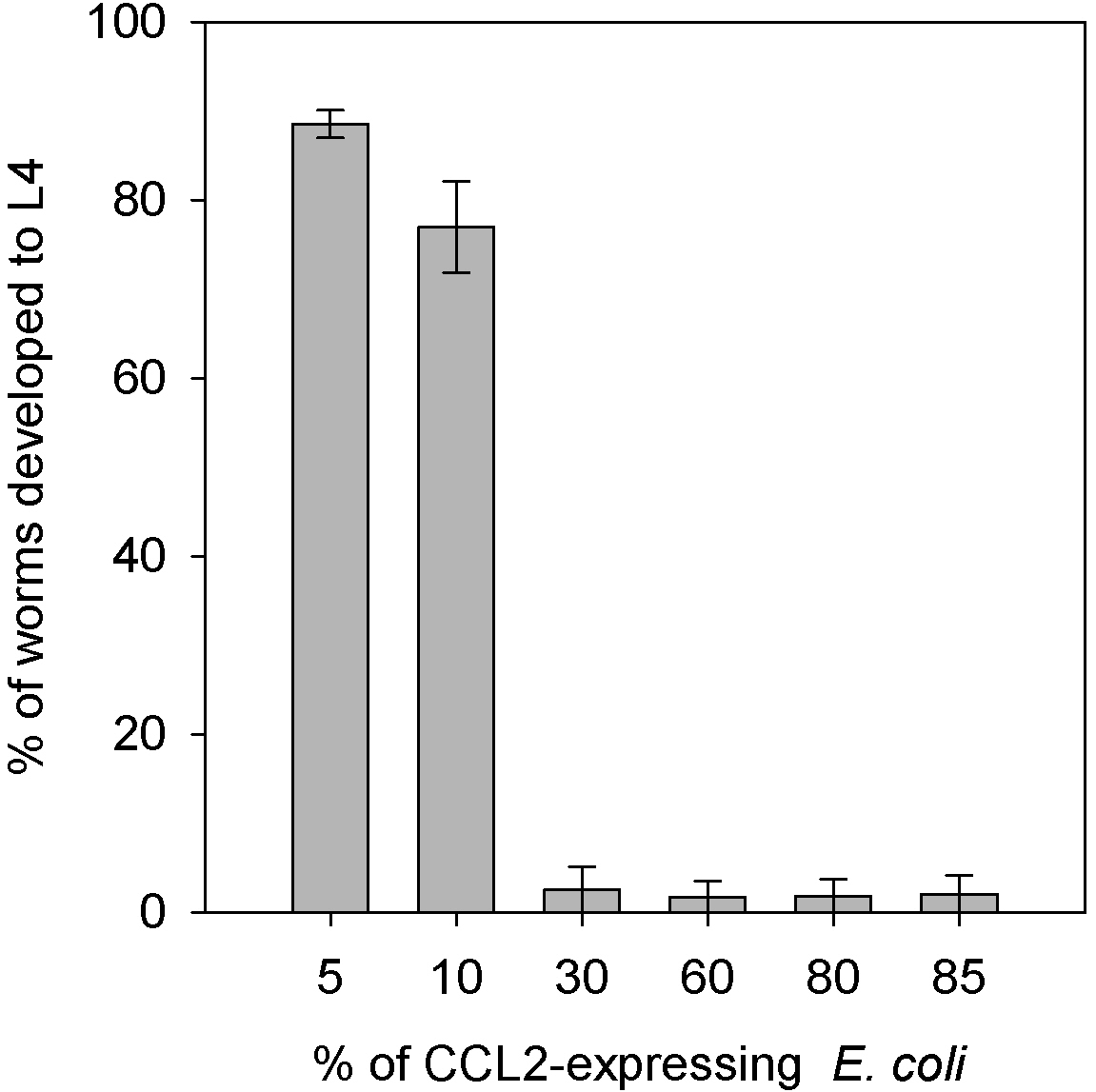

Supplement: Figure S9 — Dose-dependence of CCL2-mediated nematotoxicity. Wildtype C. elegans (N2) were fed with mixtures of CCL2-expressing E. coli expressing CCL2 and empty vector-containing E. coli. Error bars indicate standard errors of the mean. (TIF) [file ppat.1002706.s009.tif]

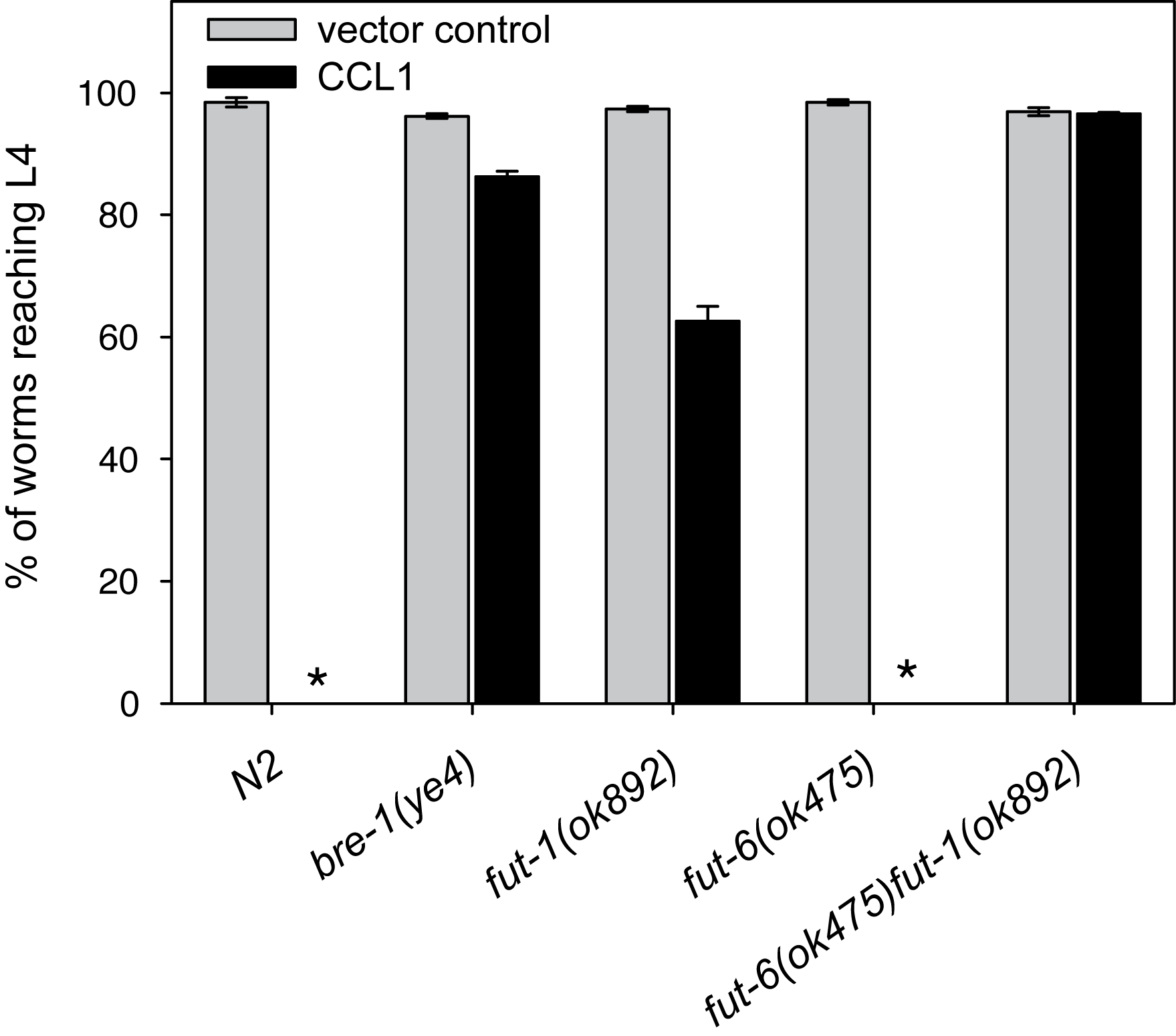

Supplement: Figure S10 — Carbohydrate-binding dependence of CCL1-mediated nematotoxicity. Toxicity of CCL1-expressing E. coli towards C. elegans wild type (N2) and various fucosylation mutants. Error bars indicate standard errors of the mean. Asterisks (*) show cases where all data were 0. Assays were done in solid media as described [30]. (TIF) [file ppat.1002706.s010.tif]

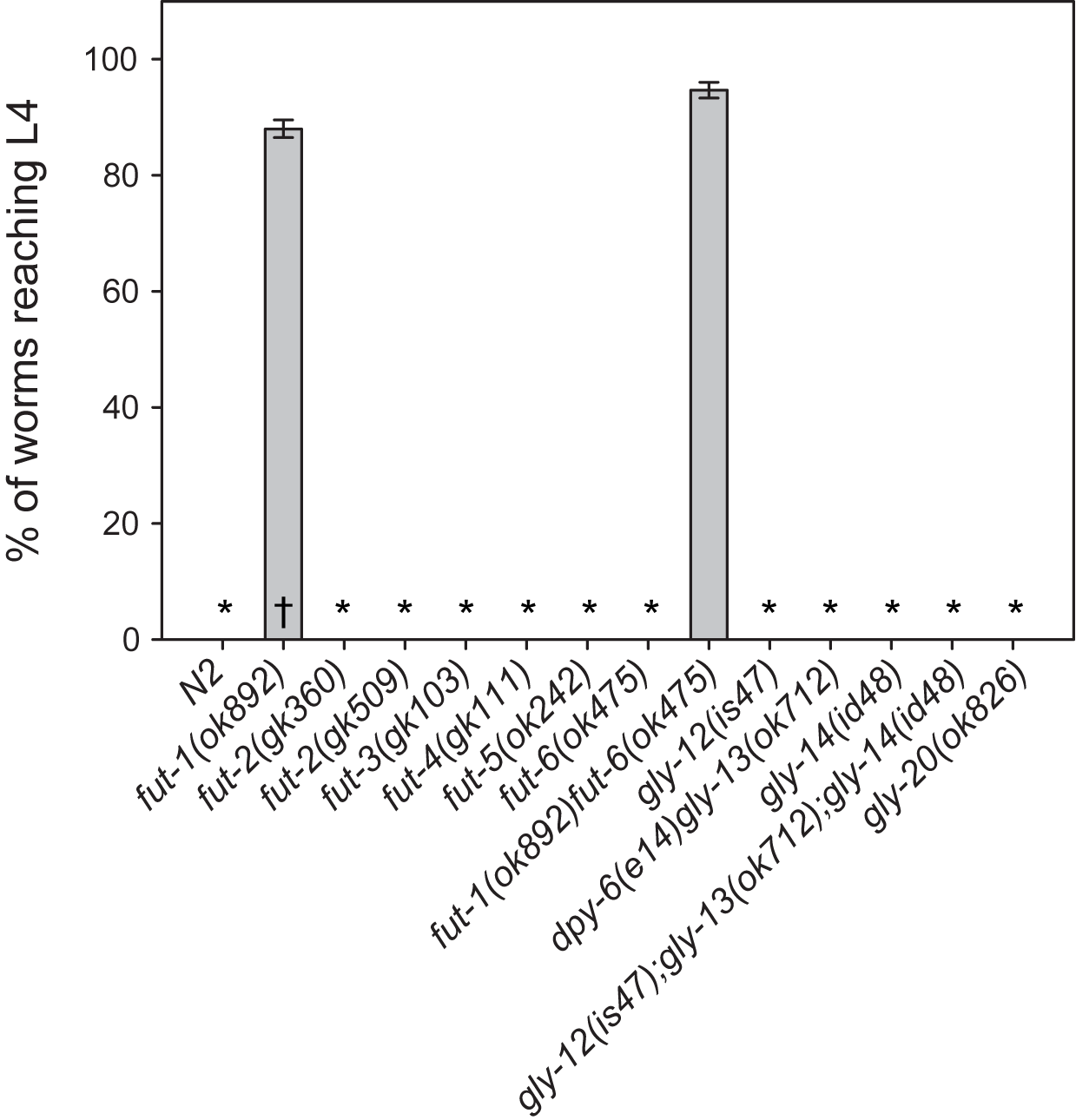

Supplement: Figure S11 — Toxicity of recombinant E. coli expressing CCL2 towards C. elegans wildtype (N2) and various mutants in predicted or characterized fucosyltransferases (fut) or GlcNAc-transferases (gly). Assays were done in solid media as described [30]. Error bars indicate standard errors of the mean. Asterisks (*) show cases where all data were 0. †: In the fut-1(ok892) mutants a partial resistance is observed. Although the larvae survive and develop, they require at least 24 h more to reach L4 and look thinner and paler than the complete resistant double mutant fut-6(ok475)fut-1(ok892). (TIF) [file ppat.1002706.s011.tif]

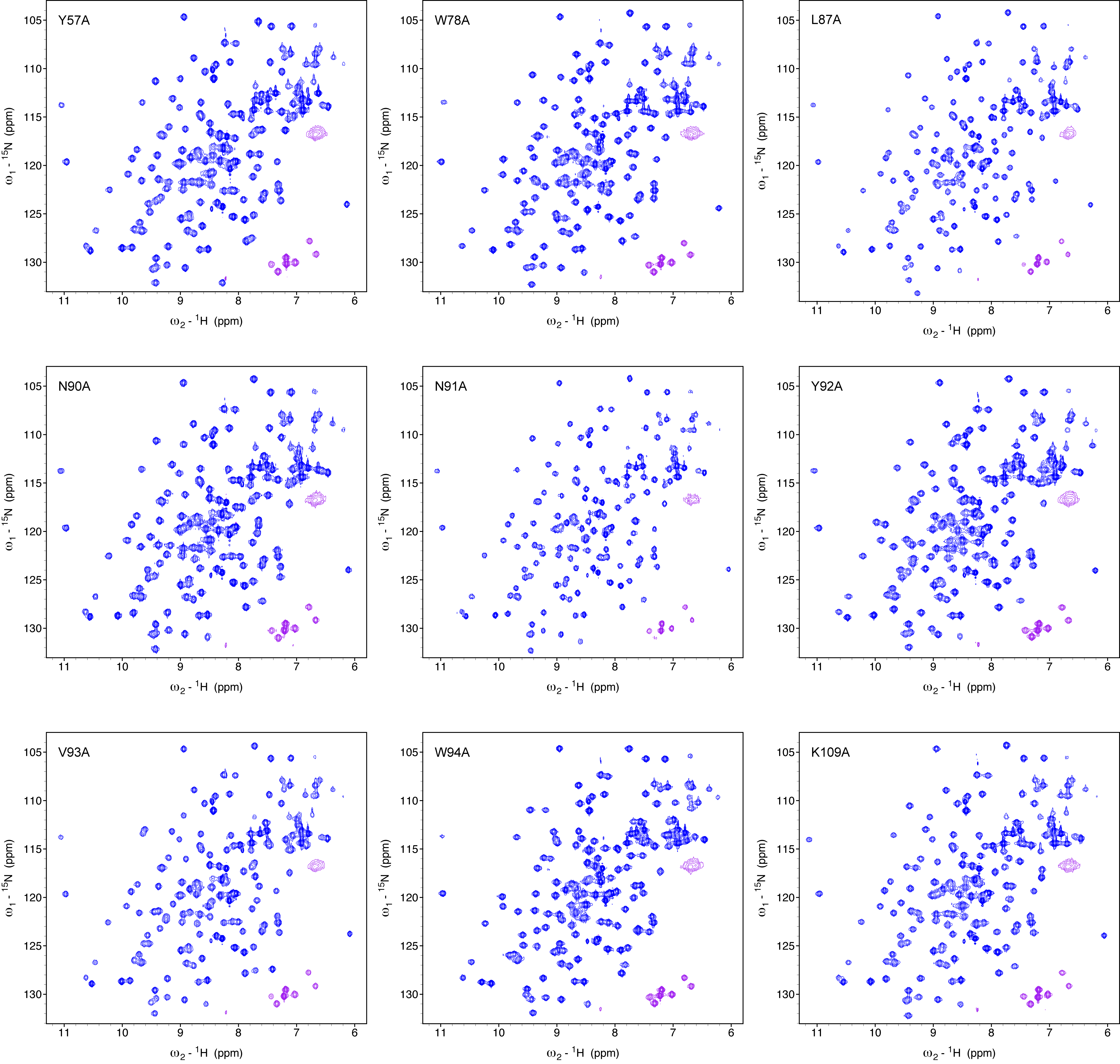

Supplement: Figure S12 — All CCL2 proteins containing a point mutant are folded. 15N-HSQC spectra of 15N labelled proteins. (TIF) [file ppat.1002706.s012.tif]

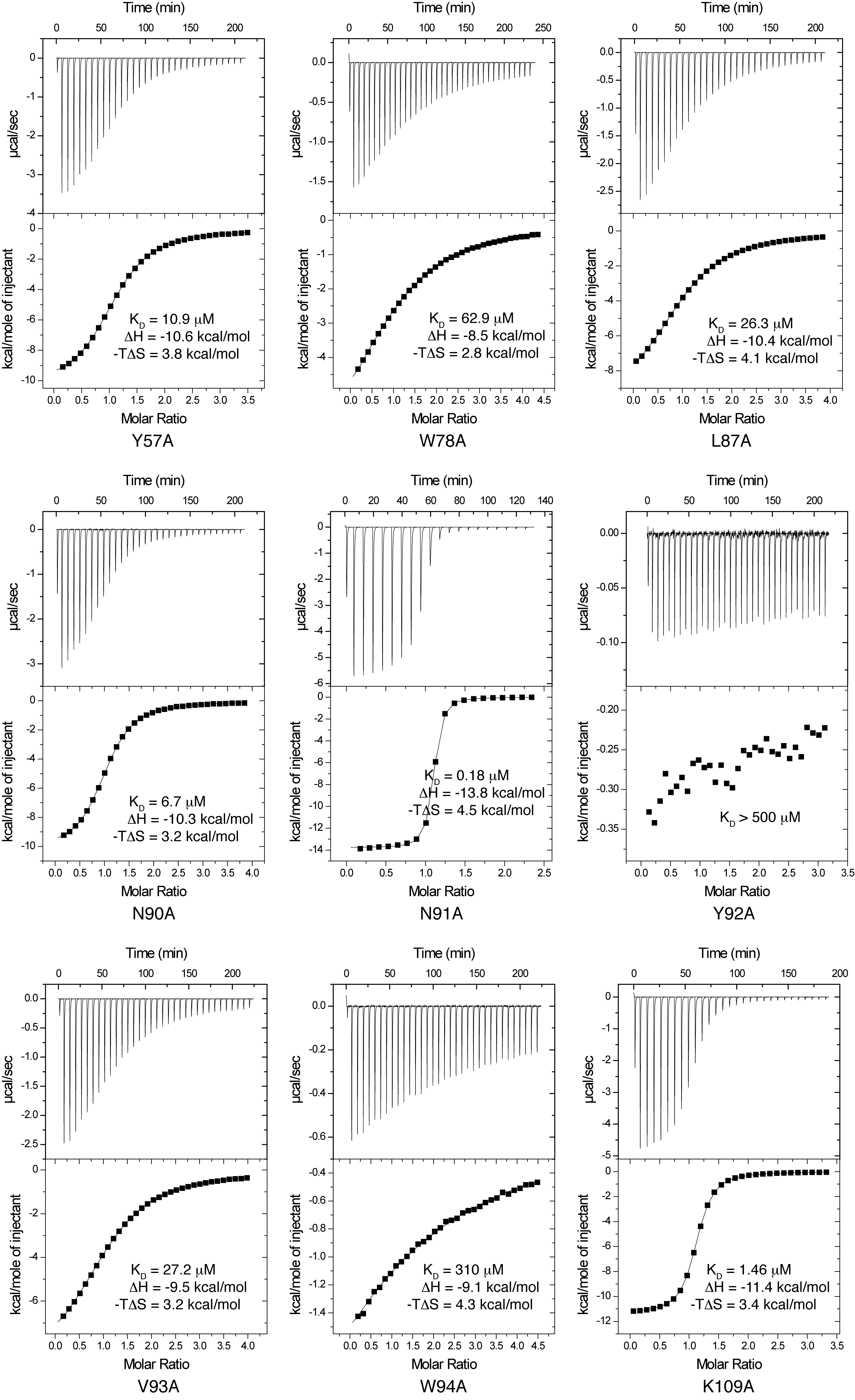

Supplement: Figure S13 — Isothermal titration calorimetry binding experiments between CCL2 mutants and fucosylated chitobiose (GlcNAcβ1,4[Fucα1,3]GlcNAcβ1-spacer). Raw calorimetric outputs are shown on the top and binding isotherms describing the complex formation are shown at the bottom. The protein concentration in the cell was 70 µM and the carbohydrate concentration was 3.0 mM. (TIF) [file ppat.1002706.s013.tif]
