# Supplementary material for: Plasticity of the β-Trefoil Protein Fold in the Recognition and Control of Invertebrate Predators and Parasites by a Fungal Defence System
Source: PLoS Pathog. 2012 May 17;8(5):e1002706. doi: 10.1371/journal.ppat.1002706 (PMC3355094; doi:10.1371/journal.ppat.1002706)
Supplement: Table S1 — Sequence identities among the CCL2 homologues and the lectin MOA and SNA-II. (PDF) [file ppat.1002706.s014.pdf]

Table S1. Sequence identities among the CCL2 orthologues found in fungi and the lectin MOA and SNA-II. CCL2_A: CCL2 of *C. cinerea* strain AmutBmut; CCL2_O: CCL2 of *C. cinerea* strain Okayama7; CCL1_A: CCL1 of *C. cinerea* strain AmutBmut; CCL1_O: CCL1 of *C. cinerea* strain Okayama7; PP_L1: *Postia placenta* lectin 1 (Pospl1_130016); PP_L2: *Postia placenta* lectin 2 (Pospl1_121916); SL_L1: *Serpula lacrymans* lectin 1 (SerlaS7_144703); CP_L1: *Coniophora puteana* lectin 1 (Conpu1_119225); PO_L1: *Pleurotus ostreatus* lectin 1 (PleosPC9_89828); PO_L2: *Pleurotus ostreatus* lectin 2 (PleosPC15_1043947); PO_L3: *Pleurotus ostreatus* lectin 3 (PleosPC9_64199); PO_L4: *Pleurotus ostreatus* lectin 4 (PleosPC15_1065820); DS_L1: *Dicomitus squalis* lectin 1 (Dicsq1); AO_L1: *Arthrobotrys oligospora* lectin 1 (s00075g2); LB_L1: Laccaria bicolor lectin 1 (Lbic_330799); LB_L2: *Laccaria bicolor lectin* 2 (Lbic_327918); MOA: *Marasmius oreades* agglutinin; SNA-II: *Sambucus nigra* agglutinin/ribosome inactivating protein type II.

	CCL2_A	CCL2_O	CCL1_A	CCL1_O	PP_L1	PP_L2	SL_L1	CP_L1	PO_L1	PO_L2	PO_L3	PO_L4	DS_L1	AO_L2	LB_L1	LB_L2	MOA	SNA-II
CCL2_A	—	93%	54%	52%	44%	44%	39%	37%	42%	42%	40%	34%	33%	30%	34%	24%	14%	13%
CCL2_O		—	54%	52%	42%	42%	40%	39%	44%	44%	40%	35%	33%	30%	34%	25%	14%	12%
CCL1_A			—	97%	42%	42%	48%	44%	40%	40%	34%	34%	36%	32%	32%	20%	14%	11%
CCL1_O				—	42%	42%	48%	43%	39%	38%	34%	34%	35%	32%	32%	20%	13%	10%
PP_L1					—	95%	56%	54%	43%	44%	42%	42%	39%	34%	39%	29%	14%	15%
PP_L2						—	55%	54%	43%	44%	42%	43%	39%	36%	40%	28%	14%	15%
SL_L1							—	54%	47%	48%	43%	40%	45%	38%	37%	27%	16%	14%
CP_L1								—	46%	45%	40%	38%	40%	33%	37%	26%	14%	12%
PO_L1									—	88%	66%	50%	38%	37%	34%	24%	11%	12%
PO_L2										—	62%	47%	37%	35%	37%	26%	11%	12%
PO_L3											—	45%	34%	34%	36%	22%	13%	16%
PO_L4												—	31%	34%	29%	23%	13%	9%
DS_L1													—	31%	29%	24%	14%	12%
AO_L2														—	28%	20%	12%	10%
LB_L1															—	26%	15%	12%
LB_L2																—	15%	13%
MOA																	—	9%
SNA-II																		—
