# Supplementary material for: Plasticity of the β-Trefoil Protein Fold in the Recognition and Control of Invertebrate Predators and Parasites by a Fungal Defence System
Source: PLoS Pathog. 2012 May 17;8(5):e1002706. doi: 10.1371/journal.ppat.1002706 (PMC3355094; doi:10.1371/journal.ppat.1002706)
Supplement: Table S2 — Raw data of glycan array analysis performed with CCL2. RFU = Relative Fluorescence Units; SD = Standard deviation. (PDF) [file ppat.1002706.s015.pdf]

Table S2. Raw data of glycan array analysis performed with CCL2. RFU = Relative Fluorescence Units; SD = Standard deviation

No.	Glycan structure – spacer*	RFU	SD
1	Neu5Acα2-8Neu5Acβ-Sp17	155.0	88.2
2	Neu5Acα2-8Neu5Acα2-8Neu5AcβSp8	66.6	38.8
3	Neu5Gcβ2-6Galβ1-4GlcNAc-Sp8	182.6	74.3
4	Galβ1-3GlcNAcβ1-2Manα1-3(Galβ1-3GlcNAcβ1-2Manα1-6)Manβ1-4GlcNAcβ1-4GlcNAcβ-Sp19	453.7	150.8
5	Neu5Acα2-6Galβ1-4GlcNAcβ1-2Manα1-3(Neu5Acα2-6Galβ1-4GlcNAcβ1-2Manα1-6)Manβ1-4GlcNAcβ1-4GlcNAcβ-Sp12	183.9	43.5
6	α-D-Gal–Sp8	280.7	94.2
7	α-D-Glc–Sp8	594.5	365.1
8	α-D-Man–Sp8	152.9	88.5
9	α-GalNAc–Sp8	151.8	60.8
10	α-L-Fuc–Sp8	416.6	386.4
11	α-L-Fuc–Sp9	136.8	10.1
12	α-L-Rha-Sp8	112.5	57.6
13	α-Neu5Ac–Sp8	247.4	195.8
14	α-Neu5Ac–Sp11	119.3	140.3
15	β-Neu5Ac-Sp8	122.4	61.6
16	β-D-Gal–Sp8	194.7	83.6
17	β-D-Glc–Sp8	113.2	9.4
18	β-D-Man–Sp8	130.5	60.3
19	β-GalNAc–Sp8	273.6	160.5
20	β-GlcNAc–Sp0	172.7	36.2
21	β-GlcNAc–Sp8	160.7	63.6
22	β-GlcN(Gc)-Sp8	136.2	139.4
23	(Galβ1-4GlcNAcβ)2-3,6-GalNAcα-Sp8	397.4	201.5
24	GlcNAcβ1-3(GlcNAcβ1-4)(GlcNAcβ1-6)GlcNAc-Sp8	103.1	41.2
25	[3OSO3][6OSO3]Galβ1-4[6OSO3]GlcNAcβ-Sp0	367.7	238.4
26	[3OSO3][6OSO3]Galβ1-4GlcNAcβ-Sp0	193.4	34.3
27	[3OSO3]Galβ1-4Glcβ-Sp8	152.3	96.0
28	[3OSO3]Galβ1-4(6OSO3)Glcβ–Sp0	151.3	50.4
29	[3OSO3]Galβ1-4(6OSO3)Glcβ–Sp8	1039.1	720.1
30	[3OSO3]Galβ1-3(Fucα1-4)GlcNAcβ–Sp8	628.7	695.7
31	[3OSO3]Galβ1-3GalNAcα-Sp8	216.8	113.9
32	[3OSO3]Galβ1-3GlcNAcβ–Sp8	403.9	425.3
33	[3OSO3]Galβ1-4(Fucα1-3)GlcNAcβ–Sp8	265.1	74.5
34	[3OSO3]Galβ1-4[6OSO3]GlcNAcβ-Sp8	101.0	90.0
35	[3OSO3]Galβ1-4GlcNAcβ–Sp0	115.6	12.2
36	[3OSO3]Galβ1-4GlcNAcβ-Sp8	110.8	72.3
37	[3OSO3]Galβ–Sp8	75.5	25.5
38	[4OSO3][6OSO3]Galβ1-4GlcNAcβ-Sp0	222.7	221.8
39	[4OSO3]Galβ1-4GlcNAcβ-Sp8	350.4	63.8
40	6-H2PO3Manα-Sp8	118.9	53.1
41	[6OSO3]Galβ1-4Glcβ–Sp0	307.4	46.8
42	[6OSO3]Galβ1-4Glcβ–Sp8	269.4	180.9
43	[6OSO3]Galβ1-4GlcNAcβ–Sp8	133.9	115.9
44	[6OSO3]Galβ1-4[6OSO3]Glcβ-Sp8	298.1	154.7
45	NeuAcα2-3[6OSO3]Galβ1-4GlcNAcβ–Sp8	40316.6	1380.1
46	[6OSO3]GlcNAcβ–Sp8	141.7	114.3
47	9NAcNeu5Acα-Sp8	8595.4	918.0
48	9NAcNeu5Acα2-6Galβ1-4GlcNAcβ-Sp8	239.4	174.8
49	Manα1-3(Manα1-6)Manβ1-4GlcNAcβ1-4GlcNAcβ-Sp13	525.3	166.5
50	GlcNAcβ1-2Manα1-3(GlcNAcβ1-2Manα1-6)Manβ1-4GlcNAcβ1-4GlcNAcβ-Sp13	674.3	270.7
51	Galβ1-4GlcNAcβ1-2Manα1-3(Galβ1-4GlcNAcβ1-2Manα1-6)Manβ1-4GlcNAcβ1-	575.8	464.2

	4GlcNAcβ-Sp13		
52	Neu5Acα2-6Galβ1-4GlcNAcβ1-2Manα1-3(Neu5Acα2-6Galβ1-4GlcNAcβ1-2Manα1-6)Manβ1-4GlcNAcβ1-4GlcNAcβ-Sp13	336.0	84.3
53	Neu5Acα2-6Galβ1-4GlcNAcβ1-2Manα1-3(Neu5Acα2-6Galβ1-4GlcNAcβ1-2Manα1-6)Manβ1-4GlcNAcβ1-4GlcNAcβ-Sp8	535.8	285.5
54	Fucα1-2Galβ1-3GalNAcβ1-3Gala-Sp9	854.8	233.8
55	Fucα1-2Galβ1-3GalNAcβ1-3Galα1-4Galβ1-4Glcβ-Sp9	146.7	31.0
56	Fucα1-2Galβ1-3(Fucα1-4)GlcNAcβ–Sp8	101.3	43.1
57	Fucα1-2Galβ1-3GalNAcα-Sp8	83.3	18.4
58	Fucα1-2Galβ1-3GalNAcβ1-4(Neu5Acα2-3)Galβ1-4Glcβ-Sp0	108.9	42.3
59	Fucα1-2Galβ1-3GalNAcβ1-4(Neu5Acα2-3)Galβ1-4Glcβ-Sp9	190.1	16.2
60	Fucα1-2Galβ1-3GlcNAcβ1-3Galβ1-4Glcβ–Sp10	99.7	48.5
61	Fucα1-2Galβ1-3GlcNAcβ1-3Galβ1-4Glcβ–Sp8	261.3	84.7
62	Fucα1-2Galβ1-3GlcNAcβ–Sp0	154.5	95.8
63	Fucα1-2Galβ1-3GlcNAcβ–Sp8	197.8	44.3
64	Fucα1-2Galβ1-4(Fucα1-3)GlcNAcβ1-3Galβ1-4(Fucα1-3)GlcNAcβ-Sp0	495.0	369.3
65	Fucα1-2Galβ1-4(Fucα1-3)GlcNAcβ1-3Galβ1-4(Fucα1-3)GlcNAcβ1-3Galβ1-4(Fucα1-3)GlcNAcβ-Sp0	1323.8	228.2
66	Fucα1-2Galβ1-4(Fucα1-3)GlcNAcβ–Sp0	80.7	29.0
67	Fucα1-2Galβ1-4(Fucα1-3)GlcNAcβ–Sp8	3466.0	907.7
68	Fucα1-2Galβ1-4GlcNAcβ1-3Galβ1-4GlcNAc–Sp0	57.3	54.0
69	Fucα1-2Galβ1-4GlcNAcβ1-3Galβ1-4GlcNAcβ1-3Galβ1-4GlcNAcβ-Sp0	282.4	119.0
70	Fucα1-2Galβ1-4GlcNAcβ–Sp0	246.8	94.3
71	Fucα1-2Galβ1-4GlcNAcβ–Sp8	288.4	84.2
72	Fucα1-2Galβ1-4Glcβ–Sp0	250.1	152.4
73	Fucα1-2Galβ–Sp8	200.0	100.4
74	Fucα1-3GlcNAcβ-Sp8	211.4	160.5
75	Fucα1-4GlcNAcβ–Sp8	183.1	9.3
76	Fucβ1-3GlcNAcβ-Sp8	297.6	398.7
77	GalNAcα1-3(Fucα1-2)Galβ1-3GlcNAcβ-Sp0	107.7	46.0
78	GalNAcα1-3(Fucα1-2)Galβ1-4(Fucα1-3)GlcNAcβ-Sp0	192.0	73.9
79	GalNAcα1-3(Fucα1-2)Galβ1-4GlcNAcβ-Sp0	193.0	98.0
80	GalNAcα1-3(Fucα1-2)Galβ1-4GlcNAcβ–Sp8	104.3	38.4
81	GalNAcα1-3(Fucα1-2)Galβ1-4Glcβ-Sp0	94.5	79.5
82	GalNAcα1-3(Fucα1-2)Galβ–Sp8	90.7	10.7
83	GalNAcα1-3GalNAcβ–Sp8	162.6	41.6
84	GalNAcα1-3Galβ–Sp8	176.6	38.9
85	GalNAcα1-4(Fucα1-2)Galβ1-4GlcNAcβ-Sp8	248.7	68.9
86	GalNAcβ1-3GalNAcα-Sp8	123.7	70.5
87	GalNAcβ1-3(Fucα1-2)Galβ-Sp8	304.1	176.9
88	GalNAcβ1-3Galα1-4Galβ1-4GlcNAcβ-Sp0	204.6	208.7
89	GalNAcβ1-4(Fucα1-3)GlcNAcβ-Sp0	40042.3	1963.4
90	GalNAcβ1-4GlcNAcβ–Sp0	285.8	388.3
91	GalNAcβ1-4GlcNAcβ–Sp8	117.5	83.8
92	Galα1-2Galβ–Sp8	119.5	55.8
93	Galα1-3(Fucα1-2)Galβ1-3GlcNAcβ-Sp0	91.2	21.8
94	Galα1-3(Fucα1-2)Galβ1-4(Fucα1-3)GlcNAcβ-Sp0	236.8	52.3
95	Galα1-3(Fucα1-2)Galβ1-4GlcNAc-Sp0	113.8	39.2
96	Galα1-3(Fucα1-2)Galβ1-4Glcβ-Sp0	64.7	27.1
97	Galα1-3(Fucα1-2)Galβ–Sp8	300.8	78.4
98	Galα1-3(Galα1-4)Galβ1-4GlcNAcβ-Sp8	298.6	276.1
99	Galα1-3GalNAcα-Sp8	445.6	175.2
100	Galα1-3GalNAcβ–Sp8	856.8	310.3
101	Galα1-3Galβ1-4(Fucα1-3)GlcNAcβ–Sp8	10567.5	1155.2
102	Galα1-3Galβ1-3GlcNAcβ-Sp0	95.8	24.3
103	Galα1-3Galβ1-4GlcNAcβ–Sp8	165.9	121.6
104	Galα1-3Galβ1-4Glcβ–Sp0	52.1	21.0
105	Galα1-3Galβ–Sp8	31.4	22.3

106	Galα1-4(Fucα1-2)Galβ1-4GlcNAcβ-Sp8	133.8	72.7
107	Galα1-4Galβ1-4GlcNAcβ–Sp0	86.7	20.3
108	Galα1-4Galβ1-4GlcNAcβ–Sp8	257.8	45.7
109	Galα1-4Galβ1-4Glcβ–Sp0	123.3	43.5
110	Galα1-4GlcNAcβ–Sp8	610.1	876.5
111	Galα1-6Glcβ-Sp8	236.9	167.1
112	Galβ1-2Galβ–Sp8	109.0	41.8
113	Galβ1-3(Fucα1-4)GlcNAcβ1-3Galβ1-4(Fucα1-3)GlcNAcβ-Sp0	457.2	336.6
114	Galβ1-3(Fucα1-4)GlcNAcβ1-3Galβ1-4GlcNAcβ-Sp0	24347.1	2789.4
115	Galβ1-3(Fucα1-4)GlcNAc–Sp0	115.3	16.2
116	Galβ1-3(Fucα1-4)GlcNAc–Sp8	97.0	69.6
117	Galβ1-3(Fucα1-4)GlcNAcβ–Sp8	98.4	53.2
118	Galβ1-3(Galβ1-4GlcNAcβ1-6)GalNAcα-Sp8	105.8	31.3
119	Galβ1-3(GlcNAcβ1-6)GalNAcα-Sp8	103.1	40.4
120	Galβ1-3(Neu5Acα2-6)GalNAcα-Sp8	183.1	117.7
121	Galβ1-3(Neu5Acβ2-6)GalNAcα-Sp8	154.0	33.0
122	Galβ1-3(Neu5Acα2-6)GlcNAcβ1-4Galβ1-4Glcβ-Sp10	154.4	92.3
123	Galβ1-3GalNAcα-Sp8	244.6	147.8
124	Galβ1-3GalNAcβ–Sp8	159.6	127.1
125	Galβ1-3GalNAcβ1-3Galα1-4Galβ1-4Glcβ-Sp0	154.2	53.9
126	Galβ1-3GalNAcβ1-4(Neu5Acα2-3)Galβ1-4Glcβ-Sp0	64.6	19.7
127	Galβ1-3GalNAcβ1-4Galβ1-4Glcβ–Sp8	95.5	93.4
128	Galβ1-3Galβ–Sp8	106.4	21.5
129	Galβ1-3GlcNAcβ1-3Galβ1-4GlcNAcβ-Sp0	119.3	29.1
130	Galβ1-3GlcNAcβ1-3Galβ1-4Glcβ–Sp10	188.3	32.2
131	Galβ1-3GlcNAcβ–Sp0	117.3	33.5
132	Galβ1-3GlcNAcβ–Sp8	138.7	45.6
133	Galβ1-4(Fucα1-3)GlcNAcβ–Sp0	7830.5	6655.6
134	Galβ1-4(Fucα1-3)GlcNAcβ–Sp8	8611.1	3385.6
135	Galβ1-4(Fucα1-3)GlcNAcβ1-4Galβ1-4(Fucα1-3)GlcNAcβ-Sp0	35683.2	863.8
136	Galβ1-4(Fucα1-3)GlcNAcβ1-4Galβ1-4(Fucα1-3)GlcNAcβ1-4Galβ1-4(Fucα1-3)GlcNAcβ–Sp0	30025.6	536.4
137	Galβ1-4[6OSO3]Glcβ–Sp0	160.0	132.5
138	Galβ1-4[6OSO3]Glcβ–Sp8	96.1	25.5
139	Galβ1-4GalNAcα1-3(Fucα1-2)Galβ1-4GlcNAcβ-Sp8	235.3	81.3
140	Galβ1-4GalNAcβ1-3(Fucα1-2)Galβ1-4GlcNAcβ-Sp8	319.4	225.3
141	Neu5Acα2-3Galβ1-4GlcNAcβ1-2Manα1-3(Neu5Acα2-3Galβ1-4GlcNAcβ1-2Manα1-6)Manβ1-4GlcNAcβ1β1-4GlcNAcβ-Sp12	316.8	240.3
142	Galβ1β1-4GlcNAcβ1β1-3GalNAcα-Sp8	188.0	82.4
143	Galβ1β1-4GlcNAcβ1-3Galβ1-4(Fucα1-3)GlcNAcβ1-3Galβ1-4(Fucα1-3)GlcNAcβ-Sp0	33357.1	1008.9
144	Galβ1-4GlcNAcβ1-3Galβ1-4GlcNAcβ1-3Galβ1-4GlcNAcβ–Sp0	145.9	37.9
145	Galβ1-4GlcNAcβ1-3Galβ1-4GlcNAcβ–Sp0	87.6	118.0
146	Galβ1-4GlcNAcβ1-3Galβ1-4Glcβ–Sp0	263.9	75.0
147	Galβ1-4GlcNAcβ1-3Galβ1-4Glcβ–Sp8	37.1	85.8
148	Galβ1-4GlcNAcβ1-6(Galβ1-3)GalNAcα-Sp8	137.2	71.5
149	Galβ1-4GlcNAcβ1-6GalNAcα-Sp8	139.4	7.6
150	Galβ1-4GlcNAcβ–Sp0	165.4	72.6
151	Galβ1-4GlcNAcβ–Sp8	84.7	33.1
152	Galβ1-4Glcβ–Sp0	76.3	70.4
153	Galβ1-4Glcβ–Sp8	119.7	27.0
154	GlcNAcα1-3Galβ1-4GlcNAcβ-Sp8	113.3	43.4
155	GlcNAcα1-6Galβ1-4GlcNAcβ-Sp8	158.8	60.0
156	GlcNAcβ1-2Galβ1-3GalNAcα-Sp8	219.7	122.7
157	GlcNAcβ1-3(GlcNAcβ1-6)GalNAcα-Sp8	219.8	150.8
158	GlcNAcβ1-3(GlcNAcβ1-6)Galβ1-4GlcNAcb–Sp8	74.0	19.0
159	GlcNAcβ1-3GalNAcα-Sp8	45.0	56.9
160	GlcNAcβ1-3Galβ-Sp8	70.4	30.7
161	GlcNAcβ1-3Galβ1-3GalNAcα-Sp8	325.8	92.3

162	GlcNAcβ1-3Galβ1-4GlcNAcβ–Sp0	62.3	43.3
163	GlcNAcβ1-3Galβ1-4GlcNAcβ-Sp8	161.9	75.3
164	GlcNAcβ1-3Galβ1-4GlcNAcβ1-3Galβ1-4GlcNAcβ-Sp0	165.9	83.8
165	GlcNAcβ1-3Galβ1-4Glcβ–Sp0	91.8	39.9
166	GlcNAcβ1-4MDPLys	182.7	81.5
167	GlcNAcβ1-4(GlcNAcβ1-6)GalNAcα-Sp8	159.7	28.4
168	GlcNAcβ1-4Galβ1-4GlcNAcβ-Sp8	59.3	54.8
169	(GlcNAcβ1-4)6β-Sp8	465.2	132.9
170	(GlcNAcβ1-4)5β-Sp8	388.9	141.3
171	GlcNAcβ1-4GlcNAcβ1-4GlcNAcb–Sp8	457.8	146.9
172	GlcNAcβ1-6(Galβ1-3)GalNAcα-Sp8	190.8	64.7
173	GlcNAcβ1-6GalNAcα-Sp8	35.9	67.7
174	GlcNAcβ1-6Galβ1-4GlcNAcβ-Sp8	157.0	51.4
175	Glcα1-4Glcβ–Sp8	44.7	81.9
176	Glcα1-4Glcα-Sp8	59.7	14.7
177	Glcα1-6Glcα1-6Glcβ-Sp8	42.3	50.3
178	Glcβ1-4Glcβ-Sp8	38.6	13.0
179	Glcβ1-6Glcβ-Sp8	101.4	32.3
180	G-ol-Sp8	103.1	14.8
181	GlcAα-Sp8	348.3	225.8
182	GlcAβ-Sp8	285.1	226.5
183	GlcAβ1-3Galβ-Sp8	246.0	98.7
184	GlcAβ1-6Galβ-Sp8	160.2	71.1
185	KDNa2-3Galβ1-3GlcNAcβ–Sp0	375.8	360.0
186	KDNa2-3Galβ1-4GlcNAcβ–Sp0	147.2	45.4
187	Manα1-2Manα1-2Manα1-3Manα-Sp9	1134.1	254.5
188	Manα1-2Manα1-3(Manα1-2Manα1-6)Manα-Sp9	208.0	130.8
189	Manα1-2Manα1-3Manα-Sp9	142.4	69.9
190	Manα1-6(Manα1-2Manα1-3)Manα1-6(Manα2Manα1-3)Manβ1-4GlcNAcβ1-4GlcNAcβ-Sp12	130.1	26.3
191	Manα1-2Manα1-6(Manα1-3)Manα1-6(Manα2Mana2Manα1-3)Manβ1-4GlcNAcβ1-4GlcNAcβ-Sp12	62.3	25.9
192	Manα1-2Manα1-2Manα1-3(Manα1-2Manα1-3(Manα1-2Manα1-6)Manα1-6)Manβ1-4GlcNAcβ1-4GlcNAcβ-Sp12	104.4	82.1
193	Manα1-3(Manα1-6)Maαa-Sp9	312.6	113.9
194	Manα1-3(Manα1-2Manα1-2Manα1-6)Manα-Sp9	365.1	219.3
195	Manα1-6(Manα1-3)Manα1-6(Mana2Manα1-3)Manβ1-4GlcNAcβ1-4GlcNAcβ-Sp12	219.0	43.3
196	Manα1-6(Manα1-3)Manα1-6(Manα1-3)Manβ1-4GlcNAcβ1-4 GlcNAcβ-Sp12	573.5	117.5
197	Neu5Acα2-6Galβ1-4GlcNAcβ1-2Manα1-3(Neu5Acα2-3Galβ1-4GlcNAcβ1-2Manα1-6)Manβ1-4GlcNAcβ1-4GlcNAcβ-Sp12	302.8	115.7
198	Manβ1-4GlcNAcβ-Sp0	36.3	20.1
199	Fucα1-3(Galβ1-4)GlcNAcβ1-2Manα1-3(Fucα1-3(Galβ1-4)GlcNAcβ1-2Manα1-6)Manβ1-4GlcNAcβ1-4GlcNAcβ-Sp20	29817.8	1325.1
200	Neu5Acα2-3Galβ1-3GalNAcα-Sp8	276.0	115.2
201	NeuAcα2-8NeuAcα2-8NeuAcα2-8NeuAcα2-3(GalNAcβ1-4)Galβ1-4Glcβ-Sp0	145.1	100.3
202	Neu5Acα2-8Neu5Acα2-8Neu5Acα2-3(GalNAcβ1-4)Galβ1-4Glcβ-Sp0	413.8	307.5
203	Neu5Acα2-8Neu5Acα2-8Neu5Acα2-3Galβ1-4Glcβ–Sp0	211.6	53.8
204	Neu5Acα2-8Neu5Acα2-3(GalNAcβ1-4)Galβ1-4Glcβ–Sp0	271.9	51.3
205	Neu5Acα2-8Neu5Acα2-8Neu5Acα-Sp8	389.8	131.9
206	Neu5Acα2-3(6-O-Su)Galβ1-4(Fucα1-3)GlcNAcβ–Sp8	34863.4	1455.7
207	Neu5Acα2-3(GalNAcβ1-4)Galβ1-4GlcNAcβ-Sp0	301.6	116.7
208	Neu5Acα2-3(GalNAcβ1-4)Galβ1-4GlcNAcβ-Sp8	117.1	61.9
209	Neu5Acα2-3(GalNAcβ1-4)Galβ1-4Glcβ–Sp0	276.8	138.6
210	NeuAcα2-3(NeuAcα2-3Galβ1-3GalNAcβ1-4)Galβ1-4Glcβ-β-Sp0	434.6	50.8
211	Neu5Acα2-3(Neu5Acα2-6)GalNAcα-Sp8	326.1	208.1
212	Neu5Acα2-3GalNAcα-Sp8	114.4	22.2
213	Neu5Acα2-3GalNAcβ1-4GlcNAcβ-Sp0	82.6	67.6
214	Neu5Acα2-3Galβ1-3(6OSO3)GlcNAc-Sp8	177.0	114.8
215	Neu5Acα2-3Galβ1-3(Fucα1-4)GlcNAcβ–Sp8	171.7	45.0

216	NeuAcα2-3Galβ1-3(Fucα1-4)GlcNAcβ1-3Galβ1-4(Fucα1-3)GlcNAcβ Sp0	1565.8	249.1
217	Neu5Acα2-3Galβ1-3(Neu5Acα2-3Galβ1-4)GlcNAcβ-Sp8	357.4	127.0
218	Neu5Acα2-3Galβ1-3[6OSO3]GalNAcα-Sp8	560.4	101.5
219	Neu5Acα2-3Galβ1-3(Neu5Acα2-6)GalNAcα-Sp8	285.6	74.0
220	Neu5Acα2-3Galβ-Sp8	629.4	483.7
221	NeuAcα2-3Galβ1-3GalNAcβ1-3Galα1-4Galβ1-4Glcβ-β-Sp0	207.0	41.4
222	NeuAcα2-3Galβ1-3GlcNAcβ1-3Galβ1-4GlcNAcβ-Sp0	70.9	35.5
223	Neu5Acα2-3Galβ1-3GlcNAcβ–Sp0	197.3	108.0
224	Neu5Acα2-3Galβ1-3GlcNAcβ–Sp8	154.9	86.6
225	Neu5Acα2-3Galβ1-4[6OSO3]GlcNAcβ-Sp8	309.3	158.8
226	Neu5Acα2-3Galβ1-4(Fucα1-3)(6OSO3)GlcNAcb–Sp8	36947.0	2195.1
227	Neu5Acα2-3Galβ1-4(Fucα1-3)GlcNAcβ1-3Galβ1-4(Fucα1-3)GlcNAcβ1-3Galβ1-4(Fucα1-3)GlcNAcb–Sp0	34978.0	2173.3
228	Neu5Acα2-3Galβ1-4(Fucα1-3)GlcNAcβ–Sp0	30564.6	2432.0
229	Neu5Acα2-3Galβ1-4(Fucα1-3)GlcNAcβ–Sp8	32433.2	1831.5
230	Neu5Acα2-3Galβ1-4(Fucα1-3)GlcNAcβ1-3Galβ-Sp8	44306.4	3354.3
231	Neu5Acα2-3Galβ1-4(Fucα1-3)GlcNAcβ1-3Galβ1-4GlcNAcβ-Sp8	40692.5	2620.9
232	Neu5Acα2-3Galβ1-4GlcNAcβ1-3Galβ1-4(Fucα1-3)GlcNAc-Sp0	258.4	57.3
233	Neu5Acα2-3Galβ1-4GlcNAcβ1-3Galβ1-4GlcNAcβ1-3Galβ1-4GlcNAcβ–Sp0	283.7	128.1
234	Neu5Acα2-3Galβ1-4GlcNAcβ–Sp0	28.4	106.5
235	Neu5Acα2-3Galβ1-4GlcNAcβ–Sp8	383.4	521.5
236	Neu5Acα2-3Galβ1-4GlcNAcβ1-3Galβ1-4GlcNAcβ-Sp0	163.9	62.0
237	Neu5Acα2-3Galβ1-4Glcβ–Sp0	161.7	34.0
238	Neu5Acα2-3Galβ1-4Glcβ–Sp8	232.4	241.6
239	Galβ1-4GlcNAcβ1-2Manα1-3(Fucα1-3(Galβ1-4)GlcNAcβ1-2Manα1-6)Manβ1-4GlcNAcβ1-4GlcNAcβ-Sp20	45.2	24.4
240	Neu5Acα2-6GalNAcα-Sp8	220.3	68.6
241	Neu5Acα2-6GalNAcβ1-4GlcNAcβ-Sp0	218.5	106.9
242	Neu5Acα2-6Galβ1-4[6OSO3]GlcNAcβ-Sp8	775.4	202.0
243	Neu5Acα2-6Galβ1-4GlcNAcβ–Sp0	200.0	117.0
244	Neu5Acα2-6Galβ1-4GlcNAcβ–Sp8	127.0	38.3
245	Neu5Acα2-6Galβ1-4GlcNAcβ1-3Galβ1-4(Fucα1-3)GlcNAcβ1-3Galβ1-4(Fucα1-3)GlcNAcβ-Sp0	17423.1	1436.2
246	Neu5Acα2-6Galβ1-4GlcNAcβ1-3Galβ1-4GlcNAcβ-Sp0	82.4	44.5
247	Neu5Acα2-6Galβ1-4Glcβ–Sp0	101.5	51.7
248	Neu5Acα2-6Galβ1-4Glcβ–Sp8	132.3	60.9
249	Neu5cα2-Acα2-6Galβ–Sp8	121.8	14.0
250	Neu5Acα2-8Neu5Acα-Sp8	134.5	55.2
251	Neu5Acα2-8Neu5cα2-Acα2-3Galβ1-4Glcβ–Sp0	236.7	115.3
252	Neu5Acβ2-6GalNAcα-Sp8	129.7	83.9
253	Neu5Acβ2-6Galβ1-4GlcNAcβ-Sp8	558.5	305.7
254	Galβ1-4GlcNAcβ1-2Manα1-3(Neu5cα2-Acα2-6Galβ1-4GlcNAcβ1-2Manα1-6)Manβ1-4GlcNAcβ1-4GlcNAcβ-Sp21	174.0	40.1
255	Neu5Gcα2-3Galβ1-3(Fucα1-4)GlcNAcβ-Sp0	206.0	31.5
256	Neu5Gcα2-3Galβ1-3GlcNAcβ-Sp0	175.6	32.7
257	Neu5Gcα2-3Galβ1-4(Fucα1-3)GlcNAcβ-Sp0	23519.8	570.8
258	Neu5Gcα2-3Galβ1-4GlcNAcβ–Sp0	51.7	24.7
259	Neu5Gcα2-3Galβ1-4Glcβ–Sp0	154.7	38.3
260	Neu5Gcα2-6GalNAcα-Sp0	178.0	35.7
261	Neu5Gcα2-6Galβ1-4GlcNAcβ–Sp0	204.2	260.3
262	Neu5Gcα-Sp8	269.8	99.7
263	[3OSO3]Galβ1-4(Fucα1-3)(6OSO3)Glc-Sp0	959.8	115.2
264	[3OSO3]Galβ1-4(Fucα1-3)Glc-Sp0	291.6	142.0
265	[3OSO3]Galβ1-4[Fucα1-3][6OSO3]GlcNAc-Sp8	613.8	110.9
266	[3OSO3]Galβ1-4[Fucα1-3]GlcNAc-Sp0	145.7	84.6
267	Fucα1-2[6OSO3]Galβ1-4GlcNAc-Sp0	126.3	81.1
268	Fucα1-2Galβ1-4[6OSO3]GlcNAc-Sp8	178.0	113.8
269	Fucα1-2[6OSO3]Galβ1-4[6OSO3]Glc-Sp0	135.7	49.3
270	Fucα1-2-(6OSO3)-Galβ1-4Glc-Sp0	192.5	138.5

271	Fucα1-2-Galβ1-4[6OSO3]Glc-Sp0	117.4	41.4
272	Galβ1-3(Fucα1-4)GlcNAcβ1-3Galβ1-3(Fucα1-4)GlcNAcβ-Sp0	319.9	113.3
273	Galβ1-3-(Galβ1-4GlcNAcβ1-6)GalNAc-Sp14	76.5	16.5
274	Galβ1-3(GlcNacβ1-6)GalNAc-Sp14	274.1	85.9
275	Galβ1-3-(Neu5Aα2-3Galβ1-4GlcNacβ1-6)GalNAc-Sp14	190.1	10.6
276	Galβ1-3GalNAc-Sp14	159.7	97.2
277	Galβ1-3GlcNAcβ1-3Galβ1-3GlcNAcβ-Sp0	173.3	82.0
278	Galβ1-4[Fucα1-3][6OSO3]GlcNAc-Sp0	22650.6	1308.3
279	Galβ1-4[Fucα1-3][6OSO3]Glc-Sp0	115.8	34.4
280	Galβ1-4(Fucα1-3)GlcNAcβ1-3Galβ1-3(Fucα1-4)GlcNAcβ-Sp0	18517.7	2530.1
281	Galβ1-4GlcNAcβ1-3Galβ1-3GlcNAcβ-Sp0	298.9	191.5
282	Neu5Acα2-3Galβ1-3GlcNAcβ1-3Galβ1-3GlcNAcβ-Sp0	808.2	772.3
283	Neu5Acα2-3Galβ1-4GlcNAcβ1-3Galβ1-3GlcNAcβ-Sp0	382.7	193.3
284	[3OSO3]Galβ1-4[6OSO3]GlcNAcβ-Sp0	345.4	367.1
285	[3OSO3][4OSO3]Galβ1-4GlcNAcβ-SpSp0	309.4	125.7
286	[6OSO3]Galβ1-4[6OSO3]GlcNAcβ-Sp0	814.5	432.7
287	6-H2PO3Glcβ-Sp10	122.8	15.9
288	Galα1-3(Fucα1-2)Galα–Sp18	144.9	55.3
289	Galα1-3GalNAcα-Sp16	186.0	98.6
290	Galβ1-3GalNAcα-Sp16	80.4	51.8
291	Galβ1-3(Neu5Acα2-3Galβ1-4(Fucα1-3)GlcNAcβ1-6)GalNAc–Sp14	33773.1	1891.6
292	Galβ1-3Galβ1-4GlcNAcβ-Sp8	118.8	47.1
293	Galβ1-4GlcNAcβ1-2Manα1-3(Neu5Acα2-6Galβ1-4GlcNAcβ1-2Manα1-6)Manβ1-4GlcNAcβ1-4GlcNAcβ-Sp12	244.1	114.7
294	Galβ1-4GlcNAcβ1-3(Galβ1-4GlcNAcβ1-6)Galβ1-4GlcNAc-Sp0	337.4	323.3
295	Galβ1-4GlcNAcβ1-3(GlcNAcβ1-6)Galβ1-4GlcNAc-Sp0	95.9	57.6
296	Galβ1-4GlcNAcα1-6Galβ1-4GlcNAcβ-Sp0	304.3	96.3
297	Galβ1-4GlcNAcβ1-6Galβ1-4GlcNAcβ-Sp0	134.4	56.2
298	GalNAcα-Sp15	377.3	413.0
299	GalNAcα1-3(Fucα1-2)Galβ–Sp18	126.3	54.6
300	GalNAcβ1-3Galβ-Sp8	288.1	123.9
301	GlcAβ1-3GlcNAcβ-Sp8	150.6	84.2
302	GlcNAcβ1-2Manα1-3(Neu5Acα2-6Galβ1-4GlcNAcβ1-2Manα1-6)Manβ1-4GlcNAcβ1-4GlcNAcβ-Sp12	217.3	114.6
303	GlcNAcβ1-2Manα1-3(GlcNAcβ1-2Manα1-6)Manβ1-4GlcNAcβ1-4GlcNAcβ-Sp12	78.9	47.4
304	GlcNAcβ1-3Man-Sp10	22.9	32.2
305	GlcNAcβ1-4GlcNAcβ-Sp10	50.8	29.5
306	GlcNAcβ1-4GlcNAcβ-Sp12	84.1	14.3
307	HOOC(CH3)CH-3-O-GlcNAcβ1-4GlcNAcβ-Sp10	252.8	143.8
308	Manα1-3(Manα1-6)Manβ1-4GlcNAcβ1-4GlcNAcβ-Sp12	494.0	213.4
309	Manα1-6Manβ-Sp10	66.6	59.5
310	Manα1-6(Manα1-3)Manα1-6(Manα1-3)Manβ-Sp10	467.6	210.1
311	Manα1-2Manα1-2Manα1-3(Manα1-2Manα1-6(Manα1-3)Manα1-6)Mana-Sp9	236.2	69.0
312	Manα1-2Manα1-2Manα1-3(Manα1-2Manα1-6(Manα1-2Manα1-3)Manα1-6)Manα-Sp9	186.4	79.4
313	Neu5Acα2-3Galβ1-3(Neu5Acα2-3Galβ1-4GlcNAcβ1-6)GalNAc–Sp14	270.5	79.9
314	Neu5Acα2-3Galβ1-3(Neu5Acα2-6)GalNAc-Sp14	17670.9	3980.3
315	Neu5Acα2-3Galβ1-3GalNAc–Sp14	1893.9	192.9
316	Neu5Acα2-3Galβ1-4GlcNAcβ1-2Manα1-3(Neu5Acα2-6Galβ1-4GlcNAcβ1-2Manα1-6)Manβ1-4GlcNAcβ1-4GlcNAcβ-Sp12	189.8	63.7
317	Neu5Acα2-6Galβ1-4GlcNAcβ1-2Manα1-3(Galβ1-4GlcNAcβ1-2Manα1-6)Manβ1-4GlcNAcβ1-4GlcNAcβ-Sp12	227.7	110.5
318	Neu5Acα2-6Galβ1-4GlcNAcβ1-2Manα1-3(GlcNAcβ1-2Manα1-6)Manβ1-4GlcNAcβ1-4GlcNAcβ-Sp12	202.1	79.0
319	Neu5Acα2-6Galβ1-4GlcNAcβ1-2Manα1-3(Neu5Acα2-6Galβ1-4GlcNAcβ1-2Manα1-6)Manβ1-4GlcNAcβ1-4GlcNAcβ-N(LT)AVL	720.7	102.5
320	Fucα1-2Galβ1-3GalNAcα-Sp14	169.4	35.8
321	Galβ1-3(Neu5Acα2-6)GalNAcα-Sp14	1132.5	238.3
322	Galβ1-4GlcNAcβ1-3GalNAc-Sp14	95.5	27.3
323	NeuAc(9Ac) α2-3Galβ1-4GlcNAcβ-Sp0	66.2	41.8
324	NeuAc(9Ac) α2-3Galβ1-3GlcNAcβ-Sp0	87.7	31.3

325	NeuAcα2-6Galβ1-4GlcNAcβ1-3Galβ1-3GlcNAcβ-Sp0	174.3	83.6
326	NeuAcα2-3Galβ1-3(Fucα1-4)GlcNAcβ1-3Galβ1-3(Fucα1-4)GlcNAcβ-Sp0	2901.9	454.3
327	NeuAcα2-6Galβ1-4GlcNAcβ1-3Galβ1-4GlcNAcβ1-3Galβ1-4GlcNAcβ-Sp0	334.1	115.7
328	Galα1-4Galβ1-4GlcNAcβ1-3Galβ1-4Glcβ-Sp0	262.5	233.3
329	GalNAcβ1-3Galα1-4Galβ1-4GlcNAcβ1-3Galβ1-4Glcβ-Sp0	37.4	36.2
330	GalNAcα1-3(Fucα1-2)Galβ1-4GlcNAcβ1-3Galβ1-4GlcNAcβ-Sp0	249.0	40.8
331	GalNAcα1-3(Fucα1-2)Galβ1-4GlcNAcβ1-3Galβ1-4GlcNAcβ1-3Galβ1-4GlcNAcβ-Sp0	579.5	199.8
332	(Neu5Acα2-3-Galβ1-3)(((Neu5Acα2-3-Galβ1-4(Fucα1-3))GlcNAcβ1-6)GalNAc–Sp14	34815.0	2170.5
333	GlcNAcα1-4Galβ1-4GlcNAcβ1-3Galβ1-4GlcNAcβ1-3Galβ1-4GlcNAcβ-Sp0	255.5	90.3
334	GlcNAcα1-4Galβ1-4GlcNAcβ-Sp0	184.3	75.1
335	GlcNAcα1-4Galβ1-3GlcNAcβ-Sp0	321.8	51.0
336	GlcNAcα1-4Galβ1-4GlcNAcβ1-3Galβ1-4Glcβ-Sp0	265.3	145.1
337	GlcNAcα1-4Galβ1-4GlcNAcβ1-3Galβ1-4(Fucα1-3)GlcNAcβ1-3Galβ1-4(Fucα1-3)GlcNAcβ-Sp0	20406.5	1020.1
338	GlcNAcα1-4Galβ1-4GlcNAcβ1-3Galβ1-4GlcNAcβ-Sp0	1060.7	214.8
339	GlcNAcα1-4Galβ1-3GalNAc-Sp14	286.7	136.6
340	Manα1-3(Neu5Acα2-6Galβ1-4GlcNAcβ1-2Manα1-6)Manβ1-4GlcNAcβ1-4GlcNAc-Sp12	87.1	37.1
341	Neu5Acα2-6Galβ1-4GlcNAcβ1-2Manα1-3(Manα1-6)Manβ1-4GlcNAcβ1-4GlcNAc-Sp12	131.2	57.2
342	Neu5Acα2-6Galβ1-4GlcNAcβ1-2Manα1-6Manβ1-4GlcNAcβ1-4GlcNAc-Sp12	92.0	58.1
343	Neu5Acα2-6Galβ1-4GlcNAcβ1-2Manα1-3Manβ1-4GlcNAcβ1-4GlcNAc-Sp12	244.8	166.6
344	Galβ1-4GlcNAcβ1-2Manα1-3Manβ1-4GlcNAcβ1-4GlcNAc-Sp12	268.8	101.8
345	Galβ1-4GlcNAcβ1-2Manα1-6Manβ1-4GlcNAcβ1-4GlcNAc-Sp12	212.6	66.7
346	Galβ1-4GlcNAcβ1-2Manα1-3(Manα1-6)Manβ1-4GlcNAcβ1-4GlcNAcβ-Sp12	127.1	46.6
347	GlcNAcβ1-2Manα1-3(GlcNAcβ1-2Manα1-6)Manβ1-4GlcNAcβ1-4(Fucα1-6)GlcNAcβ-Sp22	353.0	68.3
348	Galβ1-4GlcNAcβ1-2Manα1-3(Galβ1-4GlcNAcβ1-2Manα1-6)Manβ1-4GlcNAcβ1-4(Fucα1-6)GlcNAcβ-Sp22	264.4	94.3
349	Galβ1-3GlcNAcβ1-2Manα1-3(Galβ1-3GlcNAcβ1-2Manα1-6)Manβ1-4GlcNAcβ1-4(Fucα1-6)GlcNacβ-Sp22	170.4	104.0
350	Galβ1-3(Fucα1-4)GlcNAcβ1-2Manα1-3[Galβ1-3(Fucα1-4)GlcNAcβ1-2Manα1-6]Manβ1-4GlcNAcβ1-4GlcNAcβ-Sp19	3266.8	264.2
351	(6SO3)GlcNAcβ1-3Gal β1-4GlcNAc-β-Sp0	144.4	155.9
352	KDNa2-3Galβ1-4(Fucα1-3)GlcNAc-Sp0	455.8	164.3
353	KDNa2-6Galβ1-4GlcNAc-Sp0	172.2	109.6
354	KDNa2-3Galβ1-4Glc-Sp0	155.2	161.9
355	KDNa2-3Galβ1-3GalNAcα-Sp14	1067.3	198.9
356	Fucα1-2Galβ1-3GlcNAcβ1-2Manα1-3(Fucα1-2Galβ1-3GlcNAcβ1-2Manα1-6)Manβ1-4GlcNAcβ1-4GlcNAcβ-Sp20	356.3	167.9
357	Fucα1-2Galβ1-4GlcNAcβ1-2Manα1-3(Fucα1-2Galβ1-4GlcNAcβ1-2Manα1-6)Manβ1-4GlcNAcβ1-4GlcNAcβ-Sp20	56.3	92.5
358	Fucα1-2Galβ1-4(Fucα1-3)GlcNAcβ1-2Manα1-3[Fucα1-2Galβ1-4(Fucα1-3)GlcNAcβ1-2Manα1-6]Manβ1-4GlcNAcβ1-4GlcNAβ-Sp20	2082.7	93.4
359	Galα1-3Galβ1-4GlcNAcβ1-2Manα1-3(Galα1-3Galβ1-4GlcNAcβ1-2Manα1-6)Manβ1-4GlcNAcβ1-4GlcNAcβ-Sp20	207.4	25.6
360	Manα1-3(Galβ1-4GlcNAcβ1-2Manα1-6)Manβ1-4GlcNAcβ1-4GlcNAcβ-Sp12	94.4	84.2
361	Galβ1-3(Fucα1-4)GlcNAcβ1-2Manα1-3[Galβ1-3(Fucα1-4)GlcNAcβ1-2Man α1-6]Manβ1-4GlcNAcβ1-4(Fucα1-6)GlcNAcβ-Sp22	893.6	341.1
362	Neu5Acα2-6GlcNAcβ1-4GlcNAc-Sp21	131.6	61.5
363	Neu5Acα2-6GlcNAcβ1-4GlcNAcβ1-4GlcNAc-Sp21	94.7	33.5
364	Fucα1-2Galβ1-3GlcNAcβ1-3[Galβ1-4(Fucα1-3)GlcNAcβ1-6]Galβ1-4Glc-Sp21	10810.2	673.9
365	Galβ1-4GlcNAcβ1-2(Galβ1-4GlcNAcβ1-4)Manα1-3[Galβ1-4GlcNAcβ1-2Manα1-6]Manβ1-4GlcNAcβ1-4GlcNAc-Sp21	288.6	118.4
366	GalNAcα1-3(Fucα1-2)Galβ1-4GlcNAcβ1-2Manα1-3[GalNAcα1-3(Fucα1-2)Galβ1-4GlcNAcβ1-2Manα1-6]Manβ1-4GlcNAcβ1-4GlcNAcβ-Sp20	280.8	59.3
367	Galα1-3(Fucα1-2)Galβ1-4GlcNAcβ1-2Manα1-3[Galα1-3(Fucα1-2)Galβ1-4GlcNAcβ1-2Manα1-6]Manβ1-4GlcNAcβ1-4GlcNAcβ-Sp20	291.4	147.3
368	Galα1-3Galβ1-4(Fucα1-3)GlcNAcβ1-2Manα1-3[Galα1-3Galβ1-4(Fucα1-3)GlcNAcβ1-2Manα1-6]Manβ1-4GlcNAcβ1-4GlcNAcβ-Sp20	36390.2	567.2
369	GalNAcα1-3(Fucα1-2)Galβ1-3GlcNAcβ1-2Manα1-3[GalNAcα1-3(Fucα1-2)Galβ1-3GlcNAcβ1-2Manα1-6]Manβ1-4GlcNAcβ1-4GlcNAcβ-Sp20	421.1	129.6
370	Galα1-3(Fucα1-2)Galβ1-3GlcNAcβ1-2Manα1-3[Galα1-3(Fucα1-2)Galβ1-3GlcNAcβ1-2Manα1-6]Manβ1-4GlcNAcβ1-4GlcNAcβ-Sp20	423.2	87.9
371	Fucα1-2Galβ1-3(Fucα1-4)GlcNAcβ1-2Manα1-3[Fucα1-2Galβ1-3(Fucα1-4)GlcNAcβ1-2Manα1-6]Manβ1-4GlcNAcβ1-4GlcNAcβ-Sp19	1853.6	480.9
372	NeuAcα2-3Galβ1-4GlcNAcβ1-3GalNAc-Sp14	604.0	284.8
373	NeuAcα2-6Galβ1-4GlcNAcβ1-3GalNAc-Sp14	5153.4	733.3

374	Fucα1-3[NeuAcα2-3Galβ1-4]GlcNAcβ1-3GalNAc-Sp14	2469.4	472.5
375	GalNAcβ1-4GlcNAcβ1-2Manα1-6(GalNAcβ1-4GlcNAcβ1-2Manα1-6)Manβ1-4GlcNAcβ1-4GlcNAc-Sp12	221.0	87.4
376	Galβ1-3GalNAcα1-3(Fucα1-2)Galβ1-4Glc-Sp14	328.6	244.0
377	Galβ1-3GalNAcα1-3(Fucα1-2)Galβ1-4GlcNAc-Sp14	346.2	66.3

* Spacers**:**

Sp0	$CH_2CH_2NH_2$
Sp8	$CH_2CH_2CH_2NH_2$
Sp9	$CH_2CH_2CH_2CH_2CH_2NH_2$
Sp10	$NHCOCH_2NH$
Sp11	$OCH_2C_6H_4$-p-$NHCOCH_2NH$
Sp12	Asparagine (N)
Sp13	Glycine (G)
Sp14	Threonine (T)
Sp15	Serine (S)
Sp16	PNP ($OC_6H_4NH_2$)
Sp17	$OCH_2C_6H_4NH_2$
Sp18	$O(CH_2)_3NHCO(CH_2)_5NH_2$
Sp19	GluAsn (EN) or AsnLys (NK)
Sp20	GlyGluAsnTrp (GENR)
Sp21	$N(CH_3)$-O-$(CH_2)_2$-NH_2
Sp22	AsnSerThr (NST)
Sp23	$(OCH_2CH_2)_6NH_2$
MDPLys	Mur-L-Ala-D-iGlnβ-$(CH_2)_4NH_2$
