# Supplementary material for: Plasticity of the β-Trefoil Protein Fold in the Recognition and Control of Invertebrate Predators and Parasites by a Fungal Defence System
Source: PLoS Pathog. 2012 May 17;8(5):e1002706. doi: 10.1371/journal.ppat.1002706 (PMC3355094; doi:10.1371/journal.ppat.1002706)
Supplement: Table S3 — Raw data of glycan array analysis performed with CCL1. RFU = Relative Fluorescence Units; SD = Standard deviation. (PDF) [file ppat.1002706.s016.pdf]

Table S3. Raw data of glycan array analysis performed with CCL1. RFU = Relative Fluorescence Units; SD = Standard deviation

No.	Glycan structure – spacer*	RFU	SD
1	Galα-Sp8	99	19
2	Glcα-Sp8	138	49
3	Manα-Sp8	136	68
4	GalNAcα-Sp8	41	11
5	GalNAcα-Sp15	148	57
6	Fucα-Sp8	26	22
7	Fucα-Sp9	-10	20
8	Rhaα-Sp8	46	35
9	Neu5Acα-Sp8	113	60
10	Neu5Acα-Sp11	247	80
11	Neu5Acβ-Sp8	98	35
12	Galβ-Sp8	68	20
13	Glcβ-Sp8	25	8
14	Manβ-Sp8	7	6
15	GalNAcβ-Sp8	15	17
16	GlcNAcβ-Sp0	6	5
17	GlcNAcβ-Sp8	4	14
18	GlcN(Gc)β-Sp8	32	10
19	Galβ1-4GlcNAcβ1-3(Galβ1-4GlcNAcβ1-6)GalNAcα-Sp8	24	17
20	GlcNAcβ1-3(GlcNAcβ1-4)(GlcNAcβ1-6)GlcNAc-Sp8	264	91
21	[3OSO3][6OSO3]Galβ1-4[6OSO3]GlcNAcβ-Sp0	3	7
22	[3OSO3][6OSO3]Galβ1-4GlcNAcβ-Sp0	25	2
23	[3OSO3]Galβ1-4(Fucα1-3)[6OSO3]Glc-Sp0	250	14
24	[3OSO3]Galβ1-4Glcβ-Sp8	29	10
25	[3OSO3]Galβ1-4[6OSO3]Glcβ-Sp0	329	38
26	[3OSO3]Galβ1-4[6OSO3]Glcβ-Sp8	228	73
27	[3OSO3]Galβ1-3(Fucα1-4)GlcNAcβ-Sp8	89	14
28	[3OSO3]Galβ1-3GalNAcα-Sp8	118	11
29	[3OSO3]Galβ1-3GlcNAcβ-Sp0	93	12
30	[3OSO3]Galβ1-3GlcNAcβ-Sp8	173	83
31	[3OSO3]Galβ1-4(Fucα1-3)GlcNAc-Sp0	383	119
32	[3OSO3]Galβ1-4(Fucα1-3)GlcNAcβ-Sp8	407	76
33	[3OSO3]Galβ1-4[6OSO3]GlcNAcβ-Sp0	293	85
34	[3OSO3]Galβ1-4[6OSO3]GlcNAcβ-Sp8	333	120
35	[3OSO3]Galβ1-4GlcNAcβ-Sp0	141	21
36	[3OSO3]Galβ1-4GlcNAcβ-Sp8	110	21
37	[3OSO3]Galβ-Sp8	42	10
38	[4OSO3][6OSO3]Galβ1-4GlcNAcβ-Sp0	5	4
39	[4OSO3]Galβ1-4GlcNAcβ-Sp8	17	9
40	6-H2PO3Manα-Sp8	3	2
41	[6OSO3]Galβ1-4Glcβ-Sp0	23	6
42	[6OSO3]Galβ1-4Glcβ-Sp8	9	3
43	[6OSO3]Galβ1-4GlcNAcβ-Sp8	27	8
44	[6OSO3]Galβ1-4[6OSO3]Glcβ-Sp8	20	8
45	Neu5Acα2-3[6OSO3]Galβ1-4GlcNAcβ-Sp8	3479	693
46	[6OSO3]GlcNAcβ-Sp8	14	10
47	[9NAc]Neu5Acα-Sp8	27	4
48	[9NAc]Neu5Acα2-6Galβ1-4GlcNAcβ-Sp8	6	2
49	Manα1-3(Manα1-6)Manβ1-4GlcNAcβ1-4GlcNAcβ-Sp12	84	18
50	Manα1-3(Manα1-6)Manβ1-4GlcNAcβ1-4GlcNAcβ-Sp13	116	57
51	GlcNAcβ1-2Manα1-3(GlcNAcβ1-2Manα1-6)Manβ1-4GlcNAcβ1-4GlcNAcβ-Sp12	90	9

52	GlcNAcβ1-2Manα1-3(GlcNAcβ1-2Manα1-6)Manβ1-4GlcNAcβ1-4GlcNAcβ-Sp13	68	14
53	Galβ1-4GlcNAcβ1-2Manα1-3(Galβ1-4GlcNAcβ1-2Manα1-6)Manβ1-4GlcNAcβ1-4GlcNAcβ-Sp12	121	19
54	Neu5Acα2-6Galβ1-4GlcNAcβ1-2Manα1-3(Neu5Acα2-6Galβ1-4GlcNAcβ1-2Manα1-6)Manβ1-4GlcNAcβ1-4GlcNAcβ-N(LT)AVL	78	21
55	Neu5Acα2-6Galβ1-4GlcNAcβ1-2Manα1-3(Neu5Acα2-6Galβ1-4GlcNAcβ1-2Manα1-6)Manβ1-4GlcNAcβ1-4GlcNAcβ-Sp12	122	23
56	Neu5Acα2-6Galβ1-4GlcNAcβ1-2Manα1-3(Neu5Acα2-6Galβ1-4GlcNAcβ1-2Manα1-6)Manβ1-4GlcNAcβ1-4GlcNAcβ-Sp13	86	19
57	Fucα1-2Galβ1-3GalNAcβ1-3Galα-Sp9	129	29
58	Fucα1-2Galβ1-3GalNAcβ1-3Galα1-4Galβ1-4Glcβ-Sp9	76	16
59	Fucα1-2Galβ1-3(Fucα1-4)GlcNAcβ-Sp8	80	32
60	Fucα1-2Galβ1-3GalNAcα-Sp8	70	35
61	Fucα1-2Galβ1-3GalNAcα-Sp14	25	10
62	Fucα1-2Galβ1-3GalNAcβ1-4(Neu5Acα2-3)Galβ1-4Glcβ-Sp0	5	4
63	Fucα1-2Galβ1-3GalNAcβ1-4(Neu5Acα2-3)Galβ1-4Glcβ-Sp9	20	10
64	Fucα1-2Galβ1-3GlcNAcβ1-3Galβ1-4Glcβ-Sp8	1	5
65	Fucα1-2Galβ1-3GlcNAcβ1-3Galβ1-4Glcβ-Sp10	20	6
66	Fucα1-2Galβ1-3GlcNAcβ-Sp0	3	1
67	Fucα1-2Galβ1-3GlcNAcβ-Sp8	18	5
68	Fucα1-2Galβ1-4(Fucα1-3)GlcNAcβ1-3Galβ1-4(Fucα1-3)GlcNAcβ-Sp0	54	21
69	Fucα1-2Galβ1-4(Fucα1-3)GlcNAcβ1-3Galβ1-4(Fucα1-3)GlcNAcβ1-3Galβ1-4(Fucα1-3)GlcNAcβ-Sp0	1835	352
70	Fucα1-2Galβ1-4(Fucα1-3)GlcNAcβ-Sp0	10	6
71	Fucα1-2Galβ1-4(Fucα1-3)GlcNAcβ-Sp8	29	16
72	Fucα1-2Galβ1-4GlcNAcβ1-3Galβ1-4GlcNAcβ-Sp0	15	13
73	Fucα1-2Galβ1-4GlcNAcβ1-3Galβ1-4GlcNAcβ1-3Galβ1-4GlcNAcβ-Sp0	61	16
74	Fucα1-2Galβ1-4GlcNAcβ-Sp0	52	8
75	Fucα1-2Galβ1-4GlcNAcβ-Sp8	45	16
76	Fucα1-2Galβ1-4Glcβ-Sp0	47	21
77	Fucα1-2Galβ-Sp8	12	37
78	Fucα1-3GlcNAcβ-Sp8	30	13
79	Fucα1-4GlcNAcβ-Sp8	62	19
80	Fucβ1-3GlcNAcβ-Sp8	54	17
81	GalNAcα1-3(Fucα1-2)Galβ1-3GlcNAcβ-Sp0	100	28
82	GalNAcα1-3(Fucα1-2)Galβ1-4(Fucα1-3)GlcNAcβ-Sp0	75	16
83	[3OSO3]Galβ1-4(Fucα1-3)Glc-Sp0	140	99
84	GalNAcα1-3(Fucα1-2)Galβ1-4GlcNAcβ-Sp0	82	17
85	GalNAcα1-3(Fucα1-2)Galβ1-4GlcNAcβ-Sp8	25	8
86	GalNAcα1-3(Fucα1-2)Galβ1-4Glcβ-Sp0	2	3
87	GlcNAcβ1-3Galβ1-3GalNAcα-Sp8	11	10
88	GalNAcα1-3(Fucα1-2)Galβ-Sp8	7	6
89	GalNAcα1-3(Fucα1-2)Galβ-Sp18	19	9
90	GalNAcα1-3GalNAcβ-Sp8	7	7
91	GalNAcα1-3Galβ-Sp8	8	5
92	GalNAcα1-4(Fucα1-2)Galβ1-4GlcNAcβ-Sp8	13	5
93	GalNAcβ1-3GalNAcα-Sp8	14	10
94	GalNAcβ1-3(Fucα1-2)Galβ-Sp8	8	7
95	GalNAcβ1-3Galα1-4Galβ1-4GlcNAcβ-Sp0	18	8
96	GalNAcβ1-4(Fucα1-3)GlcNAcβ-Sp0	7887	646
97	GalNAcβ1-4GlcNAcβ-Sp0	-11	43
98	GalNAcβ1-4GlcNAcβ-Sp8	3	27
99	Galα1-2Galβ-Sp8	71	24
100	Galα1-3(Fucα1-2)Galβ1-3GlcNAcβ-Sp0	61	20
101	Galα1-3(Fucα1-2)Galβ1-3GlcNAcβ-Sp8	31	14
102	Galα1-3(Fucα1-2)Galβ1-4(Fucα1-3)GlcNAcβ-Sp0	46	11
103	Galα1-3(Fucα1-2)Galβ1-4(Fucα1-3)GlcNAcβ-Sp8	35	22
104	Galα1-3(Fucα1-2)Galβ1-4GlcNAc-Sp0	41	13

105	Galα1-3(Fucα1-2)Galβ1-4Glcβ-Sp0	79	22
106	Galα1-3(Fucα1-2)Galβ-Sp8	63	9
107	Galα1-3(Fucα1-2)Galβ-Sp18	40	46
108	Galα1-3(Galα1-4)Galβ1-4GlcNAcβ-Sp8	57	17
109	Galα1-3GalNAcα-Sp8	-7	14
110	Galα1-3GalNAcα-Sp16	9	6
111	Galα1-3GalNAcβ-Sp8	55	17
112	Galα1-3Galβ1-4(Fucα1-3)GlcNAcβ-Sp8	4988	428
113	Galα1-3Galβ1-3GlcNAcβ-Sp0	63	15
114	Galα1-3Galβ1-4GlcNAcβ-Sp8	8	2
115	Galα1-3Galβ1-4Glcβ-Sp0	42	17
116	Galα1-3Galβ-Sp8	2	2
117	Galα1-4(Fucα1-2)Galβ1-4GlcNAcβ-Sp8	4	6
118	Galα1-4Galβ1-4GlcNAcβ-Sp0	5	7
119	Galα1-4Galβ1-4GlcNAcβ-Sp8	1	5
120	Galα1-4Galβ1-4Glcβ-Sp0	15	4
121	Galα1-4GlcNAcβ-Sp8	15	10
122	Galα1-6Glcβ-Sp8	104	34
123	Galβ1-2Galβ-Sp8	117	54
124	Galβ1-3(Fucα1-4)GlcNAcβ1-3Galβ1-4(Fucα1-3)GlcNAcβ-Sp0	562	385
125	Galβ1-3(Fucα1-4)GlcNAcβ1-3Galβ1-4GlcNAcβ-Sp0	6589	666
126	Galβ1-3(Fucα1-4)GlcNAcβ-Sp0	311	95
127	Galβ1-3(Fucα1-4)GlcNAcβ-Sp8	-2	6
128	Galβ1-3(Fucα1-4)GlcNAc-Sp8	8	2
129	Galβ1-4GlcNAcβ1-6GalNAcα-Sp8	20	7
130	Galβ1-3(GlcNAcβ1-6)GalNAcα-Sp8	31	11
131	Galβ1-3(GlcNAcβ1-6)GalNAc-Sp14	87	15
132	Galβ1-3(Neu5Acα2-6)GalNAcα-Sp8	55	11
133	Galβ1-3(Neu5Acα2-6)GalNAcα-Sp14	81	21
134	Galβ1-3(Neu5Acβ2-6)GalNAcα-Sp8	11	3
135	Galβ1-3(Neu5Acα2-6)GlcNAcβ1-4Galβ1-4Glcβ-Sp10	43	14
136	Galβ1-3GalNAcα-Sp8	28	18
137	Galβ1-3GalNAcα-Sp14	17	13
138	Galβ1-3GalNAcα-Sp16	3	3
139	Galβ1-3GalNAcβ-Sp8	6	3
140	Galβ1-3GalNAcβ1-3Galα1-4Galβ1-4Glcβ-Sp0	6	5
141	Galβ1-3GalNAcβ1-4(Neu5Acα2-3)Galβ1-4Glcβ-Sp0	26	3
142	Galβ1-3GalNAcβ1-4Galβ1-4Glcβ-Sp8	6	9
143	Galβ1-3Galβ-Sp8	22	26
144	Galβ1-3GlcNAcβ1-3Galβ1-4GlcNAcβ-Sp0	6	5
145	Galβ1-3GlcNAcβ1-3Galβ1-4Glcβ-Sp10	171	75
146	Galβ1-3GlcNAcβ-Sp0	58	18
147	Galβ1-3GlcNAcβ-Sp8	46	54
148	Galβ1-4(Fucα1-3)GlcNAcβ-Sp0	3376	175
149	Galβ1-4(Fucα1-3)GlcNAcβ-Sp8	4569	467
150	Galβ1-4(Fucα1-3)GlcNAcβ1-4Galβ1-4(Fucα1-3)GlcNAcβ-Sp0	4957	140
151	Galβ1-4(Fucα1-3)GlcNAcβ1-4Galβ1-4(Fucα1-3)GlcNAcβ1-4Galβ1-4(Fucα1-3)GlcNAcβ-Sp0	7933	1082
152	Galβ1-4[6OSO3]Glcβ-Sp0	81	6
153	Galβ1-4[6OSO3]Glcβ-Sp8	90	28
154	Galβ1-4GalNAcα1-3(Fucα1-2)Galβ1-4GlcNAcβ-Sp8	51	23
155	Galβ1-4GalNAcβ1-3(Fucα1-2)Galβ1-4GlcNAcβ-Sp8	39	3
156	Galβ1-4GlcNAcβ1-3GalNAcα-Sp8	89	46
157	Galβ1-4GlcNAcβ1-3GalNAcα-Sp14	22	11
158	Galβ1-4GlcNAcβ1-3Galβ1-4(Fucα1-3)GlcNAcβ1-3Galβ1-4(Fucα1-3)GlcNAcβ-Sp0	4838	221
159	Galβ1-4GlcNAcβ1-3Galβ1-4GlcNAcβ1-3Galβ1-4GlcNAcβ-Sp0	21	10

160	Galβ1-4GlcNAcβ1-3Galβ1-4GlcNAcβ-Sp0	9	7
161	Galβ1-4GlcNAcβ1-3Galβ1-4Glcβ-Sp0	33	22
162	Galβ1-4GlcNAcβ1-3Galβ1-4Glcβ-Sp8	1	2
163	Galβ1-4GlcNAcβ1-6(Galβ1-3)GalNAcα-Sp8	19	6
164	Galβ1-3(Galβ1-4GlcNAcβ1-6)GalNAcα-Sp8	18	12
165	Galβ1-3(Galβ1-4GlcNAcβ1-6)GalNAc-Sp14	26	10
166	Galβ1-4GlcNAcβ-Sp0	5	5
167	Galβ1-4GlcNAcβ-Sp8	7	3
168	Galβ1-4Glcβ-Sp0	2	2
169	Galβ1-4Glcβ-Sp8	101	18
170	GlcNAcα1-3Galβ1-4GlcNAcβ-Sp8	55	37
171	GlcNAcα1-6Galβ1-4GlcNAcβ-Sp8	58	24
172	GlcNAcβ1-2Galβ1-3GalNAcα-Sp8	77	34
173	GlcNAcβ1-3(GlcNAcβ1-6)GalNAcα-Sp8	30	13
174	GlcNAcβ1-3(GlcNAcβ1-6)Galβ1-4GlcNAcβ-Sp8	17	4
175	GlcNAcβ1-3GalNAcα-Sp8	7	7
176	GlcNAcβ1-3GalNAcα-Sp14	31	16
177	GlcNAcβ1-3Galβ-Sp8	25	14
178	GlcNAcβ1-3Galβ1-4GlcNAcβ-Sp0	57	20
179	GlcNAcβ1-3Galβ1-4GlcNAcβ-Sp8	47	17
180	GlcNAcβ1-3Galβ1-4GlcNAcβ1-3Galβ1-4GlcNAcβ-Sp0	55	30
181	GlcNAcβ1-3Galβ1-4Glcβ-Sp0	6	5
182	GlcNAcβ1-4-MDPLys	18	23
183	GlcNAcβ1-4(GlcNAcβ1-6)GalNAcα-Sp8	13	9
184	GlcNAcβ1-4Galβ1-4GlcNAcβ-Sp8	15	4
185	GlcNAcβ1-4GlcNAcβ1-4GlcNAcβ1-4GlcNAcβ1-4GlcNAcβ1-4GlcNAcβ1-4β-Sp8	17	12
186	GlcNAcβ1-4GlcNAcβ1-4GlcNAcβ1-4GlcNAcβ1-4GlcNAcβ-Sp8	8	5
187	GlcNAcβ1-4GlcNAcβ1-4GlcNAcβ-Sp8	25	25
188	GlcNAcβ1-6(Galβ1-3)GalNAcα-Sp8	8	6
189	GlcNAcβ1-6GalNAcα-Sp8	7	4
190	GlcNAcβ1-6GalNAcα-Sp14	12	10
191	GlcNAcβ1-6Galβ1-4GlcNAcβ-Sp8	23	11
192	Glcα1-4Glcβ-Sp8	15	5
193	Glcα1-4Glcα-Sp8	111	18
194	Glcα1-6Glcα1-6Glcβ-Sp8	113	32
195	Glcβ1-4Glcβ-Sp8	145	48
196	Glcβ1-6Glcβ-Sp8	89	52
197	G-ol-Sp8	209	43
198	GlcAα-Sp8	101	15
199	GlcAβ-Sp8	317	62
200	GlcAβ1-3Galβ-Sp8	105	23
201	GlcAβ1-6Galβ-Sp8	104	28
202	KDNα2-3Galβ1-3GlcNAcβ-Sp0	53	21
203	KDNα2-3Galβ1-4GlcNAcβ-Sp0	69	32
204	Manα1-2Manα1-2Manα1-3Manα-Sp9	42	9
205	Manα1-2Manα1-3(Manα1-2Manα1-6)Manα-Sp9	36	6
206	Manα1-2Manα1-3Manα-Sp9	18	3
207	Manα1-6(Manα1-2Manα1-3)Manα1-6(Manα1-2Manα1-3)Manβ1-4GlcNAcβ1-4GlcNAcβ-Sp12	64	7
208	Manα1-2Manα1-6(Manα1-3)Manα1-6(Manα1-2Manα1-2Manα1-3)Manβ1-4GlcNAcβ1-4GlcNAcβ-Sp12	23	10
209	Manα1-2Manα1-2Manα1-3(Manα1-2Manα1-3(Manα1-2Manα1-6)Manα1-6)Manβ1-4GlcNAcβ1-4GlcNAcβ-Sp12	41	12
210	Manα1-3(Manα1-6)Manα-Sp9	8	1
211	Manα1-3(Manα1-2Manα1-2Manα1-6)Manα-Sp9	39	15
212	Manα1-6(Manα1-3)Manα1-6(Manα1-2Manα1-3)Manβ1-4GlcNAcβ1-4GlcNAcβ-Sp12	18	8

213	Manα1-6(Manα1-3)Manα1-6(Manα1-3)Manβ1-4GlcNAcβ1-4GlcNAcβ-Sp12	35	14
214	Manβ1-4GlcNAcβ-Sp0	3	1
215	Neu5Acα2-3Galβ1-4GlcNAcβ1-3Galβ1-4(Fucα1-3)GlcNAc-Sp0	60	17
216	[3OSO3]Galβ1-4(Fucα1-3)[6OSO3]GlcNAc-Sp8	209	55
217	Fucα1-2[6OSO3]Galβ1-4GlcNAc-Sp0	93	35
218	Fucα1-2Galβ1-4[6OSO3]GlcNAc-Sp8	106	22
219	Fucα1-2[6OSO3]Galβ1-4[6OSO3]Glc-Sp0	371	26
220	Neu5Acα2-3Galβ1-3GalNAcα-Sp8	48	3
221	Neu5Acα2-3Galβ1-3GalNAcα-Sp14	155	22
222	Neu5Acα2-8Neu5Acα2-8Neu5Acα2-8Neu5Acα2-3(GalNAcβ1-4)Galβ1-4Glcβ-Sp0	116	7
223	Neu5Acα2-8Neu5Acα2-8Neu5Acα2-3(GalNAcβ1-4)Galβ1-4Glcβ-Sp0	141	31
224	Neu5Acα2-8Neu5Acα2-8Neu5Acα2-3Galβ1-4Glcβ-Sp0	60	19
225	Neu5Acα2-8Neu5Acα2-3(GalNAcβ1-4)Galβ1-4Glcβ-Sp0	94	11
226	Neu5Acα2-8Neu5Acα2-8Neu5Acα-Sp8	80	17
227	Neu5Acα2-3(6-O-Su)Galβ1-4(Fucα1-3)GlcNAcβ-Sp8	7065	1203
228	Neu5Acα2-3(GalNAcβ1-4)Galβ1-4GlcNAcβ-Sp0	52	7
229	Neu5Acα2-3(GalNAcβ1-4)Galβ1-4GlcNAcβ-Sp8	35	7
230	Neu5Acα2-3(GalNAcβ1-4)Galβ1-4Glcβ-Sp0	10	2
231	Neu5Acα2-3(Neu5Acα2-3Galβ1-3GalNAcβ1-4)Galβ1-4Glcβ-Sp0	43	10
232	Neu5Acα2-3(Neu5Acα2-6)GalNAcα-Sp8	13	6
233	Neu5Acα2-3GalNAcα-Sp8	15	21
234	Neu5Acα2-3GalNAcβ1-4GlcNAcβ-Sp0	11	11
235	Neu5Acα2-3Galβ1-3[6OSO3]GlcNAc-Sp8	62	18
236	Neu5Acα2-3Galβ1-3(Fucα1-4)GlcNAcβ-Sp8	14	4
237	Neu5Acα2-3Galβ1-3(Fucα1-4)GlcNAcβ1-3Galβ1-4(Fucα1-3)GlcNAcβ-Sp0	321	259
238	Neu5Acα2-3Galβ1-3(Neu5Acα2-3Galβ1-4)GlcNAcβ-Sp8	16	9
239	Neu5Acα2-3Galβ1-3[6OSO3]GalNAcα-Sp8	60	12
240	Neu5Acα2-3Galβ1-3(Neu5Acα2-6)GalNAcα-Sp8	3	2
241	Neu5Acα2-3Galβ1-3(Neu5Acα2-6)GalNAcα-Sp14	1025	90
242	Neu5Acα2-3Galβ-Sp8	30	10
243	Neu5Acα2-3Galβ1-3GalNAcβ1-3Galα1-4Galβ1-4Glcβ-Sp0	56	23
244	Neu5Acα2-3Galβ1-3GlcNAcβ1-3Galβ1-4GlcNAcβ-Sp0	75	22
245	Fucα1-2[6OSO3]Galβ1-4Glc-Sp0	105	15
246	Neu5Acα2-3Galβ1-3GlcNAcβ-Sp0	79	17
247	Neu5Acα2-3Galβ1-3GlcNAcβ-Sp8	104	56
248	Neu5Acα2-3Galβ1-4[6OSO3]GlcNAcβ-Sp8	176	64
249	Neu5Acα2-3Galβ1-4(Fucα1-3)[6OSO3]GlcNAcβ-Sp8	5708	520
250	Neu5Acα2-3Galβ1-4(Fucα1-3)GlcNAcβ1-3Galβ1-4(Fucα1-3)GlcNAcβ1-3Galβ1-4(Fucα1-3)GlcNAcβ-Sp0	6928	521
251	Neu5Acα2-3Galβ1-4(Fucα1-3)GlcNAcβ-Sp0	5563	253
252	Neu5Acα2-3Galβ1-4(Fucα1-3)GlcNAcβ-Sp8	5530	426
253	Neu5Acα2-3Galβ1-4(Fucα1-3)GlcNAcβ1-3Galβ-Sp8	11685	250
254	Neu5Acα2-3Galβ1-4(Fucα1-3)GlcNAcβ1-3Galβ1-4GlcNAcβ-Sp8	4596	943
255	Neu5Acα2-3Galβ1-4GlcNAcβ1-3Galβ1-4GlcNAcβ1-3Galβ1-4GlcNAcβ-Sp0	23	8
256	Neu5Acα2-3Galβ1-4GlcNAcβ-Sp0	9	5
257	Neu5Acα2-3Galβ1-4GlcNAcβ-Sp8	17	12
258	Neu5Acα2-3Galβ1-4GlcNAcβ1-3Galβ1-4GlcNAcβ-Sp0	8	6
259	Fucα1-2Galβ1-4[6OSO3]Glc-Sp0	34	20
260	Neu5Acα2-3Galβ1-4Glcβ-Sp0	8	8
261	Neu5Acα2-3Galβ1-4Glcβ-Sp8	50	22
262	Neu5Acα2-6GalNAcα-Sp8	-1	5
263	Neu5Acα2-6GalNAcβ1-4GlcNAcβ-Sp0	17	8
264	Neu5Acα2-6Galβ1-4[6OSO3]GlcNAcβ-Sp8	2	5
265	Neu5Acα2-6Galβ1-4GlcNAcβ-Sp0	65	11
266	Neu5Acα2-6Galβ1-4GlcNAcβ-Sp8	89	9

267	Neu5Acα2-6Galβ1-4GlcNAcβ1-3Galβ1-4(Fucα1-3)GlcNAcβ1-3Galβ1-4(Fucα1-3)GlcNAcβ-Sp0	1606	409
268	Neu5Acα2-6Galβ1-4GlcNAcβ1-3Galβ1-4GlcNAcβ-Sp0	50	20
269	Neu5Acα2-6Galβ1-4Glcβ-Sp0	61	15
270	Neu5Acα2-6Galβ1-4Glcβ-Sp8	111	23
271	Neu5Acα2-6Galβ-Sp8	73	8
272	Neu5Acα2-8Neu5Acα-Sp8	86	5
273	Neu5Acα2-8Neu5Acα2-3Galβ1-4Glcβ-Sp0	65	11
274	Galβ1-3(Fucα1-4)GlcNAcβ1-3Galβ1-3(Fucα1-4)GlcNAcβ-Sp0	71	18
275	Neu5Acβ2-6GalNAcα-Sp8	57	12
276	Neu5Acβ2-6Galβ1-4GlcNAcβ-Sp8	55	9
277	Neu5Gcα2-3Galβ1-3(Fucα1-4)GlcNAcβ-Sp0	30	6
278	Neu5Gcα2-3Galβ1-3GlcNAcβ-Sp0	6	5
279	Neu5Gcα2-3Galβ1-4(Fucα1-3)GlcNAcβ-Sp0	3557	364
280	Neu5Gcα2-3Galβ1-4GlcNAcβ-Sp0	12	12
281	Neu5Gcα2-3Galβ1-4Glcβ-Sp0	26	3
282	Neu5Gcα2-6GalNAcα-Sp0	2	6
283	Neu5Gcα2-6Galβ1-4GlcNAcβ-Sp0	30	18
284	Neu5Gcα-Sp8	15	22
285	Galβ1-3(Neu5Acα2-3Galβ1-4GlcNAcβ1-6)GalNAcα-Sp14	25	7
286	Galβ1-3GlcNAcβ1-3Galβ1-3GlcNAcβ-Sp0	-1	2
287	Galβ1-4(Fucα1-3)[6OSO3]GlcNAc-Sp0	2665	956
288	Galβ1-4(Fucα1-3)[6OSO3]Glc-Sp0	20	13
289	Galβ1-4(Fucα1-3)GlcNAcβ1-3Galβ1-3(Fucα1-4)GlcNAcβ-Sp0	8120	1426
290	Galβ1-4GlcNAcβ1-3Galβ1-3GlcNAcβ-Sp0	83	19
291	Neu5Acα2-3Galβ1-3GlcNAcβ1-3Galβ1-3GlcNAcβ-Sp0	104	31
292	Neu5Acα2-3Galβ1-4GlcNAcβ1-3Galβ1-3GlcNAcβ-Sp0	114	12
293	[3OSO3][4OSO3]Galβ1-4GlcNAcβ-Sp0	469	83
294	[6OSO3]Galβ1-4[6OSO3]GlcNAcβ-Sp0	381	90
295	6-H2PO3Glcβ-Sp10	244	110
296	Galβ1-3(Neu5Acα2-3Galβ1-4(Fucα1-3)GlcNAcβ1-6)GalNAcα-Sp14	994	119
297	Galβ1-3Galβ1-4GlcNAcβ-Sp8	37	17
298	Galβ1-4GlcNAcβ1-2Manα1-3(Neu5Acα2-6Galβ1-4GlcNAcβ1-2Manα1-6)Manβ1-4GlcNAcβ1-4GlcNAcβ-Sp12	131	36
299	Galβ1-4GlcNAcβ1-3(Galβ1-4GlcNAcβ1-6)Galβ1-4GlcNAc-Sp0	5	29
300	Galβ1-4GlcNAcβ1-3(GlcNAcβ1-6)Galβ1-4GlcNAc-Sp0	13	24
301	Galβ1-4GlcNAcα1-6Galβ1-4GlcNAcβ-Sp0	9	13
302	Galβ1-4GlcNAcβ1-6Galβ1-4GlcNAcβ-Sp0	11	7
303	GalNAcβ1-3Galβ-Sp8	8	9
304	GlcAβ1-3GlcNAcβ-Sp8	16	7
305	GlcNAcβ1-2Manα1-3(Neu5Acα2-6Galβ1-4GlcNAcβ1-2Manα1-6)Manβ1-4GlcNAcβ1-4GlcNAcβ-Sp12	42	10
306	GlcNAcβ1-3Man-Sp10	22	3
307	GlcNAcβ1-4GlcNAcβ-Sp10	29	28
308	GlcNAcβ1-4GlcNAcβ-Sp12	15	12
309	HOOC(CH3)CH-3-O-GlcNAcβ1-4GlcNAcβ-Sp10	42	10
310	Manα1-6Manβ-Sp10	21	4
311	Manα1-6(Manα1-3)Manα1-6(Manα1-3)Manβ-Sp10	62	22
312	Manα1-2Manα1-2Manα1-3(Manα1-2Manα1-6(Manα1-3)Manα1-6)Manα-Sp9	3	4
313	Manα1-2Manα1-2Manα1-3(Manα1-2Manα1-6(Manα1-2Manα1-3)Manα1-6)Manα-Sp9	66	11
314	Neu5Acα2-3Galβ1-3(Neu5Acα2-3Galβ1-4GlcNAcβ1-6)GalNAcα-Sp14	137	27
315	Neu5Acα2-3Galβ1-4GlcNAcβ1-2Manα1-3(Neu5Acα2-6Galβ1-4GlcNAcβ1-2Manα1-6)Manβ1-4GlcNAcβ1-4GlcNAcβ-Sp12	115	17
316	Neu5Acα2-6Galβ1-4GlcNAcβ1-2Manα1-3(Galβ1-4GlcNAcβ1-2Manα1-6)Manβ1-4GlcNAcβ1-4GlcNAcβ-Sp12	80	20
317	Neu5Acα2-6Galβ1-4GlcNAcβ1-2Manα1-3(GlcNAcβ1-2Manα1-6)Manβ1-4GlcNAcβ1-4GlcNAcβ-Sp12	131	33
318	Neu5Acα2-8Neu5Acβ-Sp17	96	18

319	Neu5Acα2-8Neu5Acα2-8Neu5Acβ-Sp8	141	28
320	Neu5Gcβ2-6Galβ1-4GlcNAc-Sp8	31	12
321	Galβ1-3GlcNAcβ1-2Manα1-3(Galβ1-3GlcNAcβ1-2Manα1-6)Manβ1-4GlcNAcβ1-4GlcNAcβ-Sp19	98	34
322	Neu5Acα2-3Galβ1-4GlcNAcβ1-2Manα1-3(Neu5Acα2-3Galβ1-4GlcNAcβ1-2Manα1-6)Manβ1-4GlcNAcβ1-4GlcNAcβ-Sp12	82	6
323	Neu5Acα2-6Galβ1-4GlcNAcβ1-2Manα1-3(Neu5Acα2-3Galβ1-4GlcNAcβ1-2Manα1-6)Manβ1-4GlcNAcβ1-4GlcNAcβ-Sp12	81	11
324	Fucα1-3(Galβ1-4)GlcNAcβ1-2Manα1-3(Fucα1-3(Galβ1-4)GlcNAcβ1-2Manα1-6)Manβ1-4GlcNAcβ1-4GlcNAcβ-Sp20	1733	246
325	Neu5Ac(9Ac)α2-3Galβ1-4GlcNAcβ-Sp0	19	10
326	Neu5Ac(9Ac)α2-3Galβ1-3GlcNAcβ-Sp0	8	4
327	Neu5Acα2-6Galβ1-4GlcNAcβ1-3Galβ1-3GlcNAcβ-Sp0	9	11
328	Neu5Acα2-3Galβ1-3(Fucα1-4)GlcNAcβ1-3Galβ1-3(Fucα1-4)GlcNAcβ-Sp0	94	18
329	Neu5Acα2-6Galβ1-4GlcNAcβ1-3Galβ1-4GlcNAcβ1-3Galβ1-4GlcNAcβ-Sp0	38	9
330	Galα1-4Galβ1-4GlcNAcβ1-3Galβ1-4Glcβ-Sp0	6	7
331	GalNAcβ1-3Galα1-4Galβ1-4GlcNAcβ1-3Galβ1-4Glcβ-Sp0	21	10
332	GalNAcα1-3(Fucα1-2)Galβ1-4GlcNAcβ1-3Galβ1-4GlcNAcβ-Sp0	6	4
333	GalNAcα1-3(Fucα1-2)Galβ1-4GlcNAcβ1-3Galβ1-4GlcNAcβ1-3Galβ1-4GlcNAcβ-Sp0	200	48
334	Neu5Acα2-3-Galβ1-3(Galβ1-4(Fucα1-3)GlcNAcβ1-6)GalNAc-Sp14	1957	473
335	GlcNAcα1-4Galβ1-4GlcNAcβ1-3Galβ1-4GlcNAcβ1-3Galβ1-4GlcNAcβ-Sp0	12	4
336	GlcNAcα1-4Galβ1-4GlcNAcβ-Sp0	45	20
337	GlcNAcα1-4Galβ1-3GlcNAcβ-Sp0	39	15
338	GlcNAcα1-4Galβ1-4GlcNAcβ1-3Galβ1-4Glcβ-Sp0	67	16
339	GlcNAcα1-4Galβ1-4GlcNAcβ1-3Galβ1-4(Fucα1-3)GlcNAcβ1-3Galβ1-4(Fucα1-3)GlcNAcβ-Sp0	1655	140
340	GlcNAcα1-4Galβ1-4GlcNAcβ1-3Galβ1-4GlcNAcβ-Sp0	96	28
341	GlcNAcα1-4Galβ1-3GalNAc-Sp14	432	124
342	Manα1-3(Neu5Acα2-6Galβ1-4GlcNAcβ1-2Manα1-6)Manβ1-4GlcNAcβ1-4GlcNAc-Sp12	93	13
343	Neu5Acα2-6Galβ1-4GlcNAcβ1-2Manα1-3(Manα1-6)Manβ1-4GlcNAcβ1-4GlcNAc-Sp12	106	48
344	Neu5Acα2-6Galβ1-4GlcNAcβ1-2Manα1-6Manβ1-4GlcNAcβ1-4GlcNAc-Sp12	78	6
345	Neu5Acα2-6Galβ1-4GlcNAcβ1-2Manα1-3Manβ1-4GlcNAcβ1-4GlcNAc-Sp12	105	18
346	Galβ1-4GlcNAcβ1-2Manα1-3Manβ1-4GlcNAcβ1-4GlcNAc-Sp12	59	9
347	Galβ1-4GlcNAcβ1-2Manα1-6Manβ1-4GlcNAcβ1-4GlcNAc-Sp12	90	20
348	Galβ1-4GlcNAcβ1-2Manα1-3(Manα1-6)Manβ1-4GlcNAcβ1-4GlcNAcβ-Sp12	135	40
349	GlcNAcβ1-2Manα1-3(GlcNAcβ1-2Manα1-6)Manβ1-4GlcNAcβ1-4(Fucα1-6)GlcNAcβ-Sp22	35	19
350	Galβ1-4GlcNAcβ1-2Manα1-3(Galβ1-4GlcNAcβ1-2Manα1-6)Manβ1-4GlcNAcβ1-4(Fucα1-6)GlcNAcβ-Sp22	8	3
351	Galβ1-3GlcNAcβ1-2Manα1-3(Galβ1-3GlcNAcβ1-2Manα1-6)Manβ1-4GlcNAcβ1-4(Fucα1-6)GlcNAcβ-Sp22	8	17
352	[6OSO3]GlcNAcβ1-3Galβ1-4GlcNAc-β-Sp0	0	5
353	KDNα2-3Galβ1-4(Fucα1-3)GlcNAc-Sp0	2132	316
354	KDNα2-6Galβ1-4GlcNAc-Sp0	7	4
355	KDNα2-3Galβ1-4Glc-Sp0	19	16
356	KDNα2-3Galβ1-3GalNAcα-Sp14	14	6
357	Fucα1-2Galβ1-3GlcNAcβ1-2Manα1-3(Fucα1-2Galβ1-3GlcNAcβ1-2Manα1-6)Manβ1-4GlcNAcβ1-4GlcNAcβ-Sp20	52	16
358	Fucα1-2Galβ1-4GlcNAcβ1-2Manα1-3(Fucα1-2Galβ1-4GlcNAcβ1-2Manα1-6)Manβ1-4GlcNAcβ1-4GlcNAcβ-Sp20	53	23
359	Fucα1-2Galβ1-4(Fucα1-3)GlcNAcβ1-2Manα1-3(Fucα1-2Galβ1-4(Fucα1-3)GlcNAcβ1-2Manα1-6)Manβ1-4GlcNAcβ1-4GlcNAβ-Sp20	175	68
360	Galα1-3Galβ1-4GlcNAcβ1-2Manα1-3(Galα1-3Galβ1-4GlcNAcβ1-2Manα1-6)Manβ1-4GlcNAcβ1-4GlcNAcβ-Sp20	51	29
361	Manα1-3(Galβ1-4GlcNAcβ1-2Manα1-6)Manβ1-4GlcNAcβ1-4GlcNAcβ-Sp12	72	6

362	Galβ1-3(Fucα1-4)GlcNAcβ1-2Manα1-3(Galβ1-3(Fucα1-4)GlcNAcβ1-2Manα1-6)Manβ1-4GlcNAcβ1-4(Fucα1-6)GlcNAcβ-Sp22	116	19
363	Neu5Acα2-6GlcNAcβ1-4GlcNAc-Sp21	42	22
364	Neu5Acα2-6GlcNAcβ1-4GlcNAcβ1-4GlcNAc-Sp21	43	13
365	Fucα1-2Galβ1-3GlcNAcβ1-3(Galβ1-4(Fucα1-3)GlcNAcβ1-6)Galβ1-4Glc-Sp21	1510	465
366	Galβ1-4GlcNAcβ1-2(Galβ1-4GlcNAcβ1-4)Manα1-3(Galβ1-4GlcNAcβ1-2Manα1-6)Manβ1-4GlcNAcβ1-4GlcNAc-Sp21	28	9
367	GalNAcα1-3(Fucα1-2)Galβ1-4GlcNAcβ1-2Manα1-3(GalNAcα1-3(Fucα1-2)Galβ1-4GlcNAcβ1-2Manα1-6)Manβ1-4GlcNAcβ1-4GlcNAcβ-Sp20	8	14
368	Galα1-3(Fucα1-2)Galβ1-4GlcNAcβ1-2Manα1-3(Galα1-3(Fucα1-2)Galβ1-4GlcNAcβ1-2Manα1-6)Manβ1-4GlcNAcβ1-4GlcNAcβ-Sp20	50	13
369	Galα1-3Galβ1-4(Fucα1-3)GlcNAcβ1-2Manα1-3(Galα1-3Galβ1-4(Fucα1-3)GlcNAcβ1-2Manα1-6)Manβ1-4GlcNAcβ1-4GlcNAcβ-Sp20	2769	438
370	GalNAcα1-3(Fucα1-2)Galβ1-3GlcNAcβ1-2Manα1-3(GalNAcα1-3(Fucα1-2)Galβ1-3GlcNAcβ1-2Manα1-6)Manβ1-4GlcNAcβ1-4GlcNAcβ-Sp20	60	25
371	Galα1-3(Fucα1-2)Galβ1-3GlcNAcβ1-2Manα1-3(Galα1-3(Fucα1-2)Galβ1-3GlcNAcβ1-2Manα1-6)Manβ1-4GlcNAcβ1-4GlcNAcβ-Sp20	14	11
372	Fucα1-2Galβ1-3(Fucα1-4)GlcNAcβ1-2Manα1-3(Fucα1-2Galβ1-3(Fucα1-4)GlcNAcβ1-2Manα1-6)Manβ1-4GlcNAcβ1-4GlcNAcβ-Sp19	482	30
373	Neu5Acα2-3Galβ1-4GlcNAcβ1-3GalNAc-Sp14	28	2
374	Neu5Acα2-6Galβ1-4GlcNAcβ1-3GalNAc-Sp14	27	14
375	Neu5Acα2-3Galβ1-4(Fucα1-3)GlcNAcβ1-3GalNAcα-Sp14	339	162
376	(GalNAcβ1-4GlcNAcβ1-2Manα1-6)GalNAcβ1-4GlcNAcβ1-2Manα1-3Manβ1-4GlcNAcβ1-4GlcNAc-Sp12	12	9
377	Galβ1-3GalNAcα1-3(Fucα1-2)Galβ1-4Glc-Sp0	21	6
378	Galβ1-3GalNAcα1-3(Fucα1-2)Galβ1-4GlcNAc-Sp0	4	10
379	Galβ1-3GlcNAcβ1-3(Galβ1-3GlcNAcβ1-3Galβ1-4GlcNAcβ1-6)Galβ1-4Glcβ-Sp0	7	7
380	Galβ1-3GlcNAcβ1-3(Galβ1-4(Fucα1-3)GlcNAcβ1-6)Galβ1-4Glc-Sp21	2968	817
381	Fucα1-2Galβ1-3(Fucα1-4)GlcNAcβ1-3(Galβ1-4GlcNAcβ1-6)Galβ1-4Glc-Sp21	30	8
382	Fucα1-2Galβ1-3(Fucα1-4)GlcNAcβ1-3(Galβ1-4(Fucα1-3)GlcNAcβ1-6)Galβ1-4Glc-Sp21	1012	333
383	Galβ1-3GlcNAcβ1-3(Galβ1-3GlcNAcβ1-3Galβ1-4(Fucα1-3)GlcNAcβ1-6)Galβ1-4Glc-Sp21	1750	276
384	Galβ1-4GlcNAcβ1-2(Galβ1-4GlcNAcβ1-4)Manα1-3(Galβ1-4GlcNAcβ1-2(Galβ1-4GlcNAcβ1-6)Manα1-6)Manβ1-4GlcNAcβ1-4GlcNAcβ-Sp21	11	11
385	GlcNAcβ1-2(GlcNAcβ1-4)Manα1-3(GlcNAcβ1-2Manα1-6)Manβ1-4GlcNAcβ1-4GlcNAc-Sp21	29	28
386	Fucα1-2Galβ1-3GalNAcα1-3(Fucα1-2)Galβ1-4Glcβ-Sp0	81	24
387	Fucα1-2Galβ1-3GalNAcα1-3(Fucα1-2)Galβ1-4GlcNAcβ-Sp0	88	30
388	Galβ1-3GlcNAcβ1-3GalNAcα-Sp14	75	11
389	Neu5Acα2-3(GalNAcβ1-4)Galβ1-4GlcNAcβ1-3GalNAcα-Sp14	196	65
390	GalNAcα1-3(Fucα1-2)Galβ1-3GalNAcα1-3(Fucα1-2)Galβ1-4GlcNAcβ-Sp0	81	24
391	Galα1-3Galβ1-3GlcNAcβ1-2Manα1-3(Galα1-3Galβ1-3GlcNAcβ1-2Manα1-6)Manβ1-4GlcNAcβ1-4GlcNAc-Sp19	115	17
392	Galα1-3Galβ1-3(Fucα1-4)GlcNAcβ1-2Manα1-3(Galα1-3Galβ1-3(Fucα1-4)GlcNAcβ1-2Manα1-6)Manβ1-4GlcNAcβ1-4GlcNAc-Sp19	145	14
393	Galβ1-4GlcNAcβ1-2Manα1-3(GlcNAcβ1-2Manα1-6)Manβ1-4GlcNAcβ1-4GlcNAc-Sp12	154	30
394	GlcNAcβ1-2Manα1-3(Galβ1-4GlcNAcβ1-2Manα1-6)Manβ1-4GlcNAcβ1-4GlcNAc-Sp12	106	39
395	Neu5Acα2-3Galβ1-3GlcNAcβ1-3GalNAcα-Sp14	165	58
396	Fucα1-2Galβ1-4GlcNAcβ1-3GalNAcα-Sp14	106	40
397	Galβ1-4(Fucα1-3)GlcNAcβ1-3GalNAcα-Sp14	58	8
398	GalNAcα1-3GalNAcβ1-3Galα1-4Galβ1-4GlcNAcβ-Sp0	2	3
399	Galα1-4Galβ1-3GlcNAcβ1-2Manα1-3(Galα1-4Galβ1-3GlcNAcβ1-2Manα1-6)Manβ1-4GlcNAcβ1-4GlcNAcβ-Sp19	41	27
400	Galα1-4Galβ1-4GlcNAcβ1-2Manα1-3(Galα1-4Galβ1-4GlcNAcβ1-2Manα1-6)Manβ1-4GlcNAcβ1-4GlcNAcβ-LVANKT	10	6
401	Galα1-3Galβ1-4GlcNAcβ1-3GalNAcα-Sp14	22	15
402	Galβ1-3GlcNAcβ1-6Galβ1-4GlcNAcβ-Sp0	8	7
403	Galβ1-3GlcNAcα1-6Galβ1-4GlcNAcβ-Sp0	22	10
404	GalNAcβ1-3Galα1-6Galβ1-4Glcβ-Sp8	12	10

405	GlcNAcβ1-6(GlcNAcβ1-3)GalNAcα-Sp14	21	18
406	Galα1-3(Fucα1-2)Galβ1-4(Fucα1-3)Glcβ-Sp21	10	4
407	Neu5Acα2-6Galβ1-3GlcNAcβ1-3(Galβ1-4GlcNAcβ1-6)Galβ1-4Glc-Sp21	34	12
408	Galβ1-3GalNAcβ1-4(Neu5Acα2-8Neu5Acα2-3)Galβ1-4Glcβ-Sp0	21	9
409	Neu5Acα2-3Galβ1-3GalNAcβ1-4(Neu5Acα2-8Neu5Acα2-3)Galβ1-4Glcβ-Sp0	108	18
410	Galα1-3(Fucα1-2)Galβ1-4GlcNAcβ1-3GalNAcα-Sp14	55	13
411	GalNAcα1-3(Fucα1-2)Galβ1-4GlcNAcβ1-3GalNAcα-Sp14	147	50
412	GalNAcα1-3GalNAcβ1-3Galα1-4Galβ1-4Glcβ-Sp0	43	6
413	Fucα1-2Galβ1-4(Fucα1-3)GlcNAcβ1-3GalNAcα-Sp14	543	194
414	Galα1-3(Fucα1-2)Galβ1-4(Fucα1-3)GlcNAcβ1-3GalNAc-Sp14	104	24
415	GalNAcα1-3(Fucα1-2)Galβ1-4(Fucα1-3)GlcNAcβ1-3GalNAc-Sp14	197	77
416	Galβ1-4(Fucα1-3)GlcNAcβ1-2Manα1-3(Galβ1-4(Fucα1-3)GlcNAcβ1-2Manα1-6)Manβ1-4GlcNAcβ1-4(Fucα1-6)GlcNAcβ-Sp22	80	25
417	Fucα1-2Galβ1-4GlcNAcβ1-2Manα1-3(Fucα1-2Galβ1-4GlcNAcβ1-2Manα1-6)Manβ1-4GlcNAcβ1-4(Fucα1-6)GlcNAcβ-Sp22	143	38
418	GlcNAcβ1-2Manα1-3(GlcNAcβ1-2(GlcNAcβ1-6)Manα1-6)Manβ1-4GlcNAcβ1-4GlcNAcβ-Sp19	67	26
419	Fucα1-2Galβ1-3GlcNAcβ1-3GalNAc-Sp14	111	19
420	Galα1-3(Fucα1-2)Galβ1-3GlcNAcβ1-3GalNAc-Sp14	77	10
421	GalNAcα1-3(Fucα1-2)Galβ1-3GlcNAcβ1-3GalNAc-Sp14	54	30
422	Galα1-3Galβ1-3GlcNAcβ1-3GalNAc-Sp14	10	4
423	Fucα1-2Galβ1-3GlcNAcβ1-2Manα1-3(Fucα1-2Galβ1-3GlcNAcβ1-2Manα1-6)Manβ1-4GlcNAcβ1-4(Fucα1-6)GlcNAcβ-Sp22	49	11
424	Galα1-3(Fucα1-2)Galβ1-4GlcNAcβ1-2Manα1-3(Galα1-3(Fucα1-2)Galβ1-4GlcNAcβ1-2Manα1-6)Manβ1-4GlcNAcβ1-4(Fucα1-6)GlcNAcβ-Sp22	8	8
425	Galβ1-3GlcNAcβ1-2Manα1-3(Galβ1-3GlcNAcβ1-2(Galβ1-3GlcNAcβ1-6)Manα1-6)Manβ1-4GlcNAcβ1-4GlcNAcβ-Sp19	25	6
426	Fucα1-2Galβ1-3GlcNAcβ1-3(Galβ1-4GlcNAcβ1-6)Galβ1-4Glc-Sp21	7	2
427	Galβ1-4GlcNAcβ1-3Galβ1-4(Fucα1-3GlcNAcβ1-6)Galβ1-4Glc-Sp21	3417	656
428	GlcNAcβ1-2Manα1-3(GlcNAcβ1-4)(GlcNAcβ1-2Manα1-6)Manβ1-4GlcNacβ1-4GlcNAc-Sp21	8	9
429	GlcNAcβ1-4(GlcNAcβ1-2)Manα1-3(GlcNAcβ1-4)(GlcNAcβ1-2Manα1-6)Manβ1-4GlcNAcβ1-4GlcNAc-Sp21	20	12
430	GlcNAcβ1-2Manα1-3(GlcNAcβ1-4)(GlcNAcβ1-6(GlcNAcβ1-2)Manα1-6)Manβ1-4GlcNAcβ1-4GlcNAc-Sp21	15	5
431	GlcNAcβ1-4(GlcNAcβ1-2)Manα1-3(GlcNAcβ1-4)(GlcNAcβ1-6(GlcNAcβ1-2)Manα1-6)Manβ1-4GlcNAcβ1-4GlcNAc-Sp21	25	1
432	Galβ1-4GlcNAcβ1-2Manα1-3(GlcNAcβ1-4)(Galβ1-4GlcNAcβ1-2)Manβ1-4GlcNAcβ1-4GlcNAc-Sp21	22	9
433	Galβ1-4GlcNAcβ1-4(Galβ1-4GlcNAcβ1-2)Manα1-3(GlcNAcβ1-4)(Galβ1-4GlcNAcβ1-2Manα1-6)Manβ1-4GlcNAcβ1-4GlcNAc-Sp21	28	13
434	Galβ1-4GlcNAcβ1-2Manα1-3(GlcNAcβ1-4)(Galβ1-4GlcNAcβ1-6(Galβ1-4GlcNAcβ1-2)Manα1-6)Manβ1-4GlcNAcβ1-4GlcNAc-Sp21	61	22
435	Galβ1-4GlcNAcβ1-4(Galβ1-4GlcNAcβ1-2)Manα1-3(GlcNAcβ1-4)(Galβ1-4GlcNAcβ1-6(Galβ1-4-GlcNAcβ1-2)Manα1-6)Manβ1-4GlcNAcβ1-4GlcNAc-Sp21	63	5
436	Galα1-3Galβ1-4Glc-Sp10	52	15
437	Galβ1-4Galβ-Sp10	127	53
438	Galβ1-6Galβ-Sp10	117	41
439	Neu5Acα2-3Galβ1-4GlcNAcβ1-3Galβ-Sp8	71	19
440	GalNAcβ1-6GalNAcβ-Sp8	10	6
441	[6OSO3]Galβ1-3GlcNAcβ-Sp0	50	20
442	[6OSO3]Galβ1-3[6OSO3]GlcNAc-Sp0	482	51
443	Fucα1-2Galβ1-4GlcNAcβ1-2(Fucα1-2Galβ1-4GlcNAcβ1-4)Manα1-3(Fucα1-2Galβ1-4 GlcNAcβ1-2Manα1-6)Manβ1-4GlcNAcβ1-4GlcNAcβ-Sp12	18	32
444	Fucα1-2Galβ1-4(Fucα1-3)GlcNAcβ1-2(Fucα1-2Galβ1-4(Fucα1-3)GlcNAcβ1-4)Manα1-3(Fucα1-2Galβ1-4(Fucα1-3)GlcNAcβ1-2Manα1-6)Manβ1-4GlcNAcβ1-4GlcNAcβ-Sp12	106	31
445	Galβ1-4GlcNAcβ1-3(Galβ1-4GlcNAcβ1-6)GalNAc-Sp14	45	14
446	Galβ1-4GlcNAcβ1-6GalNAc-Sp14	30	9
447	Galβ1-4(Fucα1-3)GlcNAcβ1-6GalNAc-Sp14	992	482
448	Galβ1-4GlcNAcβ1-2Manα-Sp0	4	2

449	Fucα1-2Galβ1-4GlcNAcβ1-3(Fucα1-2Galβ1-4GlcNAcβ1-6)GalNAc-Sp14	71	28
450	Galα1-3Fucα1-2Galβ1-4GlcNAcβ1-3(Galα1-3Fucα1-2Galβ1-4GlcNAcβ1-6)GalNAc-Sp14	30	17
451	GalNAcα1-3Fucα1-2Galβ1-4GlcNAcβ1-3(GalNAcα1-3Fucα1-2Galβ1-4GlcNAcβ1-6)GalNAc-Sp14	37	25
452	Neu5Acα2-8Neu5Acα2-3Galβ1-3GalNAcβ1-4(Neu5Acα2-8Neu5Acα2-3)Galβ1-4Glcβ-Sp0	7	4
453	GalNAcβ1-4Galβ1-4Glcβ-Sp0	33	13
454	GalNAcα1-3(Fucα1-2)Galβ1-4GlcNAcβ1-2Manα1-6(GalNAcα1-3(Fucα1-2)Galβ1-4GlcNAcβ1-2Manα1-3)Manβ1-4GlcNAcβ1-4(Fucα1-6)GlcNAcβ-Sp22	2	4
455	Galα1-3(Fucα1-2)Galβ1-3GlcNAcβ1-2Manα1-6(Galα1-3(Fucα1-2)Galβ1-3GlcNAcβ1-2Manα1-3)Manβ1-4GlcNAcβ1-4(Fucα1-6)GlcNAcβ-Sp22	17	10
456	Neu5Acα2-6Galβ1-4GlcNAcβ1-6(Fucα1-2Galβ1-3GlcNAcβ1-3)Galβ-4Glc-Sp21	5	2
457	GalNAcα1-3(Fucα1-2)Galβ1-3GlcNAcβ1-2Manα1-6(GalNAcα1-3(Fucα1-2)Galβ1-3GlcNAcβ1-2Manα1-3)Manβ1-4GlcNAcβ1-4(Fucα1-6)GlcNAcβ-Sp22	80	13
458	Galβ1-4GlcNAcβ1-6(Galβ1-4GlcNAcβ1-2)Manα1-6(Galβ1-4GlcNAcβ1-2Manα1-3)Manβ1-4GlcNAcβ1-4GlcNAcβ-Sp19	87	16
459	Galβ1-4GlcNAcβ-(OCH2CH2)6NH2	7	5
460	Galα1-3(Fucα1-2)Galβ1-3GalNAcα-Sp8	21	15
461	Galα1-3(Fucα1-2)Galβ1-3GalNAcβ-Sp8	43	8
462	Glcα1-6Glcα1-6Glcα1-6Glcβ-Sp10	76	23
463	Glcα1-4Glcα1-4Glcα1-4Glcβ-Sp10	110	29
464	Neu5Acα2-3Galβ1-4GlcNAcβ1-6(Neu5Acα2-3Galβ1-4GlcNAcβ1-3)GalNAcα-Sp14	87	33
465	Fucα1-2Galβ1-4(Fucα1-3)GlcNAcβ1-2Manα1-6(Fucα1-2Galβ1-4(Fucα1-3)GlcNAcβ1-2Manα1-3)Manβ1-4GlcNAcβ1-4(Fucα1-6)GlcNAcβ-6AA	62	40
466	Fucα1-2Galβ1-3(Fucα1-4)GlcNAcβ1-2Manα1-6(Fucα1-2Galβ1-3(Fucα1-4)GlcNAcβ1-2Manα1-3)Manβ1-4GlcNAcβ1-4(Fucα1-6)GlcNAcβ-Sp19	61	27
467	Neu5Acα2-3Galβ1-3GlcNAcβ1-6(Neu5Acα2-3Galβ1-4GlcNAcβ1-2)Manα1-6(Neu5Acα2-3Galβ1-3GlcNAcβ1-2Manα1-3)Manβ1-4GlcNAcβ1-4GlcNAcβ-Sp19	74	15
468	GlcNAcβ1-6(GlcNAcβ1-2)Manα1-6(GlcNAcβ1-2Manα1-3)Manβ1-4GlcNAcβ1-4(Fucα1-6)GlcNAcβ-6AA	26	18
469	Galβ1-3GlcNAcβ1-2Manα1-6(GlcNAcβ1-4)(Galβ1-3GlcNAcβ1-2Manα1-3)Manβ1-4GlcNAcβ1-4GlcNAcβ-Sp21	53	23
470	Neu5Acα2-6Galβ1-4GlcNAcβ1-6(Galβ1-3GlcNAcβ1-3)Galβ1-4Glcβ-Sp21	6	7
471	Neu5Acα2-3Galβ1-4GlcNAcβ1-2Manα-Sp0	192	163
472	Neu5Acα2-3Galβ1-4GlcNAcβ1-6GalNAcα-Sp14	8	3
473	Neu5Acα2-6Galβ1-4GlcNAcβ1-6GalNAcα-Sp14	84	29
474	Neu5Acα2-6Galβ1-4 GlcNAcβ1-6(Neu5Acα2-6Galβ1-4GlcNAcβ1-3)GalNAcα-Sp14	15	6
475	Neu5Acα2-6Galβ1-4GlcNAcβ1-2Manα1-6(Neu5Acα2-6Galβ1-4GlcNAcβ1-2Manα1-3)Manβ1-4GlcNAcβ1-4(Fucα1-6)GlcNAcβ-6AA	39	11
476	Neu5Acα2-3Galβ1-4GlcNAcβ1-2Manα1-6(Neu5Acα2-3Galβ1-4GlcNAcβ1-2Manα1-3)Manβ1-4GlcNAcβ1-4(Fucα1-6)GlcNAcβ-6AA	36	10
477	Manα1-6(Manα1-3)Manβ1-4GlcNAcβ1-4(Fucα1-6)GlcNAcβ-Sp19	39	20
478	Galβ1-4GlcNAcβ1-6(Galβ1-4GlcNAcβ1-2)Manα1-6(Galβ1-4GlcNAcβ1-2Manα1-3)Manβ1-4GlcNAcβ1-4(Fucα1-6)GlcNAcβ-6AA	18	10
479	Neu5Acα2-3Galβ1-3GlcNAcβ1-2Manα1-6(GlcNAcβ1-4)(Neu5Acα2-3Galβ1-3GlcNAcβ1-2Manα1-3)Manβ1-4GlcNAcβ1-4GlcNAc-Sp21	21	20
480	Neu5Acα2-6Galβ1-4GlcNAcβ1-6(Fucα1-2Galβ1-4(Fucα1-3)GlcNAcβ1-3)Galβ1-4Glc-Sp21	6	11
481	Galβ1-3GlcNAcβ1-6GalNAcα-Sp14	113	11
482	Galα1-3Galβ1-3GlcNAcβ1-6GalNAcα-Sp14	86	18
483	Galβ1-3(Fucα1-4)GlcNAcβ1-6GalNacα-Sp14	195	79
484	Neu5Acα2-3Galβ1-3GlcNAcβ1-6GalNAcα-Sp14	105	29
485	[3OSO3]Galβ1-3(Fucα1-4) GlcNAcα-Sp0	125	72
486	Neu5Acα2-3Galβ1-3(Neu5Acα2-6)GlcNAcβ1-3(Fucα1-3(Galβ1-4)GlcNAcβ1-6)Galβ1-4Glc-Sp21	187	177
487	Fucα1-2Galβ1-4GlcNAcβ1-6GalNAcα-Sp14	239	94
488	Galα1-3Galβ1-4GlcNAcβ1-6GalNAcα-Sp14	8	6
489	Galβ1-4(Fucα1-3)GlcNAcβ1-2Manα-Sp0	1261	714
490	[6OSO3](Fucα1-2) Galβ1-3GlcNAcβ-Sp0	82	17

491	Galα1-3(Fucα1-2)Galβ1-4GlcNAcβ1-6GalNAcα-Sp14	300	110
492	Fucα1-2Galβ1-4GlcNAcβ1-2Manα-Sp0	111	42
493	(Fucα1-2)Galβ1-3[6OSO3]GlcNAcβ-Sp0	77	15
494	[6OSO3](Fucα1-2)Galβ1-3[6OSO3]GlcNAcβ-Sp0	148	60
495	Neu5Acα2-6GalNAcβ1-4[6OSO3]GlcNAcβ-Sp8	221	28
496	GalNAcβ1-4[6OSO3](Fucα1-3)GlcNAcβ-Sp8	15553	584
497	[3OSO3]GalNAcβ1-4(Fucα1-3)GlcNAcβ-Sp8	12725	438
498	(Fucα1-2)Galβ1-3GlcNAcβ1-6(Fucα1-2Galβ1-3GlcNAcβ1-3)GalNAcα-Sp14	99	41
499	GalNAcα1-3(Fucα1-2)Galβ1-3GlcNAcβ1-6GalNAcα-Sp14	93	37
500	GlcNAcβ1-2(GlcNAcβ1-4)Manα1-3(GlcNAcβ1-4)[GlcNAcβ1-2(GlcNAcβ1-6)Manα1-6]Manβ1-4GlcNAcβ1-4(Fucα1-6)GlcNAc-Sp21	15	14
501	Galβ1-4GlcNAcβ1-2(Gal β1-4GlcNAcβ1-4)Manα1-3(GlcNAcβ1-4)[Galβ1-4GlcNAcβ1-2(Galβ1-4GlcNAcβ1-6)Manα1-6]Manβ1-4GlcNAcβ1-4(Fucα1-6)GlcNAc-Sp21	10	6
502	Galβ1-3GlcNAcα1-3Galβ1-4GlcNAcβ-Sp8	8	2
503	Galβ1-3[6OSO3]GlcNAcβ-Sp8	45	13
504	[4OSO3][6OSO3]GalNAcβ1-4GlcNAc-Sp8	37	6
505	[6OSO3]GalNAcβ1-4GlcNAc-Sp8	78	41
506	[3OSO3]GalNAcβ1-4[3OSO3]GlcNAc-Sp8	234	128
507	GalNAcβ1-4[6OSO3]GlcNAc-Sp8	120	22
508	[3OSO3]GalNAcβ1-4GlcNAc-Sp8	65	13
509	[4OSO3]GalNacβ-Sp10	38	7
510	Galβ1-4[6PO3]GlcNAcβ-Sp0	54	27
511	[6PO3]Galβ1-4GlcNAcβ-Sp0	31	16

* Spacers:

Sp0	$CH_2CH_2NH_2$
Sp8	$CH_2CH_2CH_2NH_2$
Sp9	$CH_2CH_2CH_2CH_2CH_2NH_2$
Sp10	$NHCOCH_2NH$
Sp11	$OCH_2C_6H_4$-p-$NHCOCH_2NH$
Sp12	Asparagine (N)
Sp13	Glycine (G)
Sp14	Threonine (T)
Sp15	Serine (S)
Sp16	PNP ($OC_6H_4NH_2$)
Sp17	$OCH_2C_6H_4NH_2$
Sp18	$O(CH_2)_3NHCO(CH_2)_5NH_2$
Sp19	GluAsn (EN) or AsnLys (NK)
Sp20	GlyGluAsnTrp (GENR)
Sp21	$N(CH_3)$-O-$(CH_2)_2$-NH_2
Sp22	AsnSerThr (NST)
Sp23	$(OCH_2CH_2)_6NH_2$
MDPLys	Mur-L-Ala-D-iGlnβ-$(CH_2)_4NH_2$
