# Supplementary material for: Plasticity of the β-Trefoil Protein Fold in the Recognition and Control of Invertebrate Predators and Parasites by a Fungal Defence System
Source: PLoS Pathog. 2012 May 17;8(5):e1002706. doi: 10.1371/journal.ppat.1002706 (PMC3355094; doi:10.1371/journal.ppat.1002706)
Supplement: Table S4 — Most prominent proteins structurally closest to CCL2 (free) obtained by a DALI search [74] . (PDF) [file ppat.1002706.s017.pdf]

Table S4. Most prominent proteins structurally closest to CCL2 (free) obtained by a DALI search[1].

Protein	DALI score	Rmsd	Identity	Reference
Bacteria				
mosquitocidal toxin of *Bacillus sphaericus*	17.7	2.4 Å	16% (142 residues)	[2]
Fungi				
Marasmius oreades agglutinin (MOA)	15.5	1.9 Å	18% (128 residues)	[3]
Sclerotinia sclerotiorum agglutinin (SSA)	16.2	2.0 Å	14% (130 residues)	[4]
Plants				
Sambucus nigra agglutinin II (SNA-II)	14.4	3.8 Å	14% (122 residues)	[5]

References:

1. Holm L, Sander C (1995) Dali: a network tool for protein structure comparison. Trends Biochem Sci 20: 478-480.
2. Treiber N, Reinert DJ, Carpusca I, Aktories K, Schulz GE (2008) Structure and mode of action of a mosquitocidal holotoxin. J Mol Biol 381: 150-159.
3. Grahn EM, Winter HC, Tateno H, Goldstein IJ, Krengel U (2009) Structural characterization of a lectin from the mushroom Marasmius oreades in complex with the blood group B trisaccharide and calcium. J Mol Biol 390: 457-466.
4. Sulzenbacher G, Roig-Zamboni V, Peumans WJ, Rouge P, Van Damme EJ, et al. (2010) Crystal structure of the GalNAc/Gal-specific agglutinin from the phytopathogenic ascomycete Sclerotinia sclerotiorum reveals novel adaptation of a beta-trefoil domain. J Mol Biol 400: 715-723.
5. Maveyraud L, Niwa H, Guillet V, Svergun DI, Konarev PV, et al. (2009) Structural basis for sugar recognition, including the Tn carcinoma antigen, by the lectin SNA-II from Sambucus nigra. Proteins 75: 89-103.
