# Supplementary material for: Plasticity of the β-Trefoil Protein Fold in the Recognition and Control of Invertebrate Predators and Parasites by a Fungal Defence System
Source: PLoS Pathog. 2012 May 17;8(5):e1002706. doi: 10.1371/journal.ppat.1002706 (PMC3355094; doi:10.1371/journal.ppat.1002706)
Supplement: Table S5 — Carbohydrate 1H and 13C chemical shifts [ppm] at 293 K referenced to DSS according to [75] . Bound chemical shifts were assigned via exchange peaks between the free and the bound form and by NOEs. Bound signals were not visible in a natural abundance 13C HSQC. (PDF) [file ppat.1002706.s018.pdf]

Table S5. Carbohydrate ^{1}H and ^{13}C chemical shifts [ppm] at 293 K referenced to DSS according to [1]. Bound chemical shifts were assigned via exchange peaks between the free and the bound form and by NOEs. Bound signals were not visible in a natural abundance ^{13}C HSQC.

Chemical shifts [ppm]	GlcNAcβ1,4[Fucα1,3]GlcNAcβ1-sp free	GlcNAcβ1,4[Fucα1,3]GlcNAcβ1-sp bound
GlcNAc 1		
H1/C1	4.47 / 103.6	4.69
H2/C2	3.85 / 58.4	3.72
H3/C3	3.82 / 77.6	3.92
H4/C4	3.85 / 76.4	3.86
H5/C5	3.47 / 78.1	3.54
H6&H6′/C6	3.91 & 3.72 /62.7	3.92 & 3.71
HN2	8.25	8.54
Q2/C8	2.00 / 25.0	1.76
GlcNAc 2		
H1/C1	4.50 / 103.1	4.68
H2/C2	3.70 / 58.6	3.99
H3/C3	3.53 / 76.4	3.25
H4/C4	3.23 / 73.5	2.84
H5/C5	3.41 / 78.7	3.36
H6&H6′/C6	3.95 & 3.59 / 64.3	3.93 & 3.59
HN2	8.24	7.88
Q2/C8	2.02 / 24.9	1.78
Fuc 2′		
H1/C1	5.09 / 101.2	5.31
H2/C2	3.69 / 70.5	3.46
H3/C3	3.93 / 71.9	4.16
H4/C4	3.78 / 74.8	3.88
H5/C5	4.71 / 69.4	5.05
H6/C6	1.24 / 18.2	1.06

References:

1. Markley JL, Bax A, Arata Y, Hilbers CW, Kaptein R, et al. (1998) Recommendations for the presentation of NMR structures of proteins and nucleic acids. IUPAC-IUBMB-IUPAB Inter-Union Task Group on the Standardization of Data Bases of Protein and Nucleic Acid Structures Determined by NMR Spectroscopy. J Biomol NMR 12: 1-23.
