# Supplementary material for: Plasticity of the β-Trefoil Protein Fold in the Recognition and Control of Invertebrate Predators and Parasites by a Fungal Defence System
Source: PLoS Pathog. 2012 May 17;8(5):e1002706. doi: 10.1371/journal.ppat.1002706 (PMC3355094; doi:10.1371/journal.ppat.1002706)
Supplement: Table S6 — Thermodynamic data of selected lectin–carbohydrate interactions that are used in Fig. 7B . (PDF) [file ppat.1002706.s019.pdf]

Table S6. Thermodynamic data of selected lectin–carbohydrate interactions that are used in Figure 7B.

Lectin	ligand	K_D [μmol]	–ΔH [kJ/mol]	–TΔS [kJ/mol]	Source or reference
CCL2 WT	GlcNacβ1,4[Fucα1,3]GlcNAc-O-spacer	1.4	49.8	16.3	This study
CCL2 (N91A)	GlcNacβ1,4[Fucα1,3]GlcNAc-O-spacer	0.18	57.7	18.8	This study
Fab fragment of Anti-LeX Monoclonal antibody 291-2G3-A	Le^X-OMe	10.8	20.9	-7.1	[1]
Calreticulin	Glcα1,3Manα1,2Manα1,2Man	0.77	50.0	15.7	[2]
Calreticulin	Glcα1,3Manα1,2ManαOMe	1.8	29.9	-3.4	[3]
ConA	Manα1,6[Manα1,3]ManβOMe	2.0	60.2	27.6	[4]
Cholera toxin B subunit (CTB)	GM1os	0.043	72.4	30.8	[5]
Grifonia simplicifolia lectin 4	Fucα1,2Galβ1,3[Fucα1,4]GlcNAc-βOMe (Le^b)	17.0	49.8	22.6	[6]
Jacalin	GalαOMe	58.0	45.3	21.2	[7]
MOA	Galα1,3Galβ1,4GlcNAc	42.3	86.2	63.6	[8]
MOA	Galα1,3[Fucα1,2]Gal	36.0	86.2	61.1	[8]
PSL	Neu5Acα2,6Galβ1,4Glc	1.28	36.1	2.1	[9]
RSL	FucαOMe	0.73	42.1	7.1	[10]
RSL	2-Fucosyllactose	0.25	39.3	1.6	[10]
TeNT	GD1b	0.045	31.8	-10.0	[11]
TeNT	GT1b	0.055	52.7	11.3	[11]
WBA II	Fucα1,2Galβ1,4GlcNAcβOMe	3.2	57.3	27.7	[12]

References:

1. van Roon AM, Pannu NS, de Vrind JP, van der Marel GA, van Boom JH, et al. (2004) Structure of an anti-Lewis X Fab fragment in complex with its Lewis X antigen. Structure 12: 1227-1236.
2. Kozlov G, Pocanschi CL, Rosenauer A, Bastos-Aristizabal S, Gorelik A, et al. (2010) Structural basis of carbohydrate recognition by calreticulin. J Biol Chem 285: 38612-38620.
3. Gupta G, Gemma E, Oscarson S, Surolia A (2008) Defining substrate interactions with calreticulin: an isothermal titration calorimetric study. Glycoconj J 25: 797-802.
4. Mandal DK, Kishore N, Brewer CF (1994) Thermodynamics of lectin-carbohydrate interactions. Titration microcalorimetry measurements of the binding of N-linked carbohydrates and ovalbumin to concanavalin A. Biochemistry 33: 1149-1156.
5. Turnbull WB, Precious BL, Homans SW (2004) Dissecting the cholera toxin-ganglioside GM1 interaction by isothermal titration calorimetry. J Am Chem Soc 126: 1047-1054.
6. Lemieux RU (1996) How water provides the impetus for molecular recognition in aqueous solution. Accounts of Chemical Research 29: 373-380.
7. Arockia Jeyaprakash A, Jayashree G, Mahanta SK, Swaminathan CP, Sekar K, et al. (2005) Structural basis for the energetics of jacalin-sugar interactions: promiscuity versus specificity. J Mol Biol 347: 181-188.
8. Winter HC, Mostafapour K, Goldstein IJ (2002) The mushroom Marasmius oreades lectin is a blood group type B agglutinin that recognizes the Galalpha 1,3Gal and Galalpha 1,3Galbeta 1,4GlcNAc porcine xenotransplantation epitopes with high affinity. J Biol Chem 277: 14996-15001.
9. Tateno H, Winter HC, Goldstein IJ (2004) Cloning, expression in Escherichia coli and characterization of the recombinant Neu5Acalpha2,6Galbeta1,4GlcNAc-specific high-affinity lectin and its mutants from the mushroom Polyporus squamosus. Biochem J 382: 667-675.
10. Kostlanova N, Mitchell EP, Lortat-Jacob H, Oscarson S, Lahmann M, et al. (2005) The fucose-binding lectin from Ralstonia solanacearum. A new type of beta-propeller architecture formed by oligomerization and interacting with fucoside, fucosyllactose, and plant xyloglucan. J Biol Chem 280: 27839-27849.
11. Krell T, Greco F, Nicolai MC, Dubayle J, Renauld-Mongenie G, et al. (2003) The use of microcalorimetry to characterize tetanus neurotoxin, pertussis toxin and filamentous haemagglutinin. Biotechnol Appl Biochem 38: 241-251.
12. Srinivas VR, Bhanuprakash Reddy G, Surolia A (1999) A predominantly hydrophobic recognition of H-antigenic sugars by winged bean acidic lectin: a thermodynamic study. FEBS Lett 450: 181-185.
