# Supplementary material for: Plasticity of the β-Trefoil Protein Fold in the Recognition and Control of Invertebrate Predators and Parasites by a Fungal Defence System
Source: PLoS Pathog. 2012 May 17;8(5):e1002706. doi: 10.1371/journal.ppat.1002706 (PMC3355094; doi:10.1371/journal.ppat.1002706)
Supplement: Table S7 — Strains used in this study. (PDF) [file ppat.1002706.s020.pdf]

Table S7. Strains used in this study.

Strain	Relevant characteristics or genotype	Source or reference
E. coli		
BL21(DE3)	F^- ompT hsdSB(rB- mB-) gal dcm (λDE3)	Novagen
BL21(DE3)/pLysS	F^- ompT hsdSB(rB- mB-) gal dcm (λDE3), pLysS Cmr	Novagen
OP50	Ura^-	[1]
C. cinerea		
AmutBmut (*A43mut B43mut pab1.2*)	Mutations at the A and B mating type loci, mimics a dikaryon	[2]
C. elegans		
Bristol type (N2)	Wild type	CGC (U. of Minnesota, USA)
bre-1(ye4)	Mutant in GDP-mannose- 4,6-dehydratase	CGC (U. of Minnesota, USA)
fut-1(ok892)	Mutant in fucosyltransferase 1	CGC (U. of Minnesota, USA)
fut-6(ok475)	Mutant in fucosyltransferase 6	CGC (U. of Minnesota, USA)
fut-6(ok475)fut-1(ok892)	Double mutant in fucosyltransferases 1 and 6	This study
fut-2(gk360)	Mutant in alpha 1,2-fucosyltransferase	CGC (U. of Minnesota, USA)
fut-2(gk509)	Mutant in alpha 1,2-fucosyltransferase	CGC (U. of Minnesota, USA)
fut-4(gk111)	Mutant in fucosyltransferase 4	CGC (U. of Minnesota, USA)
gly-12(is47)	β 1,2 GnT I	CGC (U. of Minnesota, USA)
dpy-6(e14)gly-13(ok712)	β 1,2 GnT I (*dpy-6* for mapping purposes)	CGC (U. of Minnesota, USA)
gly-14(id48)	β 1,2 GnT I	CGC (U. of Minnesota, USA)
gly-12(is47)gly-13(ok712);gly-14(id48)	Triple mutant for all three β 1,2 GnT I genes	Prof. Harry Schachter (U. of Toronto, Canada)
gly-20(ok826)	β 1,2 GnT II	CGC (U. of Minnesota, USA)
D. melanogaster		
Canton-S	Wild type	Prof. E. Hafen (ETH Zürich)
A. aegypti		
Rockefeller	Laboratory strain, susceptible to insecticidals	Swiss Tropical and Public Health Institute (Basel, Switzerland)
A. castellanii		
ATCC 30234		Prof. H. Hilbi (Ludwig-Maximilians-Universität München, Germany)

References:

1. Brenner S (1974) The genetics of Caenorhabditis elegans. Genetics 77: 71-94.
2. Swamy SI, Uno I, Ishikawa T (1984) Morphogenetic effects of mutations at the A and B incompatibility factors of Coprinus cinereus. Journal of General Microbiology 130: 3219-3224.
