# Supplementary material for: Plasticity of the β-Trefoil Protein Fold in the Recognition and Control of Invertebrate Predators and Parasites by a Fungal Defence System
Source: PLoS Pathog. 2012 May 17;8(5):e1002706. doi: 10.1371/journal.ppat.1002706 (PMC3355094; doi:10.1371/journal.ppat.1002706)
Supplement: Table S8 — Oligonucleotides used in this study. Restriction sites in the oligonucleotides are underlined, and codon changes for site directed mutagenesis are in bold. (PDF) [file ppat.1002706.s021.pdf]

Table S8. Oligonucleotides used in this study. Restriction sites in the oligonucleotides are underlined, and codon changes for site directed mutagenesis are in bold.

Oligonucleotide	Sequence (5′ → 3′)	Source or reference
Cloning		
CCL2-seq fwd	AGGCCTCAGCACTCTCACTC	This study
CCL2-seq rev	GCTCTTCTGGGACTTGAGGA	This study
CCL2-fwd	GGGAATTCCATATGGACTCCCCAGCTGTGAC	This study
CCL2-rev	GGGGGGGGATCCCTAGACCTTCTCGATGACCCAG	This study
CCL2-NHis fwd	GGGAATTCCATATGGGCCATCATCATCATCATCATCATCACA<br>GCGGCGACTCCCCAGCTGTGACGCTC	This study
CCL1 fwd	CAAACCCAACTTTACTTCTTCACCC	This study
CCL1 rev	CGAGTTGTGAAAAGGTTTACGTCCA	This study
CCL1 fwd	CCCCCATATGGATACTCAGGCCAAACCCC	This study
CCL1 rev	GGGGGAATTCTCAGACCCTCTCAAAGATCCAG	This study
CCL1-NHis fwd	GGGCATATGGATCATCATCATCATCATCATCATCACACTCAG<br>GCCAAACCCCCCGCCGG	This study
dTomato fwd	GGGGGGGGATTAATGGTGAGCAAGGGCGAGG	This study
dTomato rev	GGGGGGGGATCCCTACATATGCTTGTACAGCTCGTCCATGC	This study
fut1 A	CTAAATTGGCATCCACAACCT	This study
fut1 B	GCCATTTATTAACAGTTCTCAT	This study
fut1 C	CCGGAGTAATTAGACCTGC	This study
fut6 A	GAATGCCACCATGCAACAT	This study
fut6 B	GAATTACCCATGATACTAGAT	This study
fut6 C	GCCCCAAATATCAATCTGC	This study
Site directed mutagenesis		
CCL2 Y57A	TGGATCTTAAGGAG**CGC**CGACTCGAACTCGAACACC	This study
CCL2 W78A	CAGCCAGATCGGG**GCG**GGCGCTGGTAAC	This study
CCL2 L87A	CGTCCCCGTCGTC **GCC**CCTCCCAACAAC	This study
CCL2 N90A	GTCGTCCTCCCTCCC**GCC**AACTACGTCTGGACT	This study
CCL2 N91A	GTCCTCCCTCCCAAC**GCC**TACGTCTGGACTCTG	This study
CCL2 Y92A	CTCCCTCCCAACAAC**GCC**GTCTGGACTCTGACT	This study
CCL2 V93A	CTCCCTCCCAACAACTAC**GCC**TGGACTCTGACTTTG	This study
CCL2 W94A	CCTCCCAACAACTACGTC**GCG**ACTCTGACTTTGACT	This study
CCL2 K109A	TACAACATTCAAGATGGC**GCG**AGGACCGTCTCTTGG	This study
CCL2 Y57A	TGGATCTTAAGGAGC**GCC**GACTCGAACTCGAACACC	This study
qRT-PCR		
Tubulin fwd	GTCATGTCCGGTATCACCAC	[1]
Tubulin rev	GGGAAAGGAACCATGTTGA	[1]
CCL2 fwd	CTGGTGGATACAACATTCAAGATGGC	This study
CCL2 rev	AGACCTTCTCGATGACCCAGC	This study
CCL1 fwd	TGGCGGCTATATCATCCAAG	This study
CCL1 rev	CAGACCCTCTCAAAGATCCAG	This study

References:

1. Wälti MA, Villalba C, Buser RM, Grunler A, Aebi M, et al. (2006) Targeted gene silencing in the model mushroom Coprinopsis cinerea (Coprinus cinereus) by expression of homologous hairpin RNAs. Eukaryot Cell 5: 732-744.
