# Supplementary material for: Plasticity of the β-Trefoil Protein Fold in the Recognition and Control of Invertebrate Predators and Parasites by a Fungal Defence System
Source: PLoS Pathog. 2012 May 17;8(5):e1002706. doi: 10.1371/journal.ppat.1002706 (PMC3355094; doi:10.1371/journal.ppat.1002706)
Supplement: Text S1 — Supplementary methods. (PDF) [file ppat.1002706.s022.pdf]

Supplementary Methods

Cultivation conditions. *E. coli* was cultivated on LB, M9 or NGM medium as described [1,2]. Cultivation of *C. elegans*, *A. aegypti* and *A. castellanii* for biotoxicity assays was done as described (Künzler et al., 2010). *Coprinopsis cinerea* was maintained on YMG solid medium (0.4% (w/v) yeast extract, 1% (w/v) malt extract, 0.4% (w/v) 1.5% agar) at 37°C. Cultivation conditions and techniques for harvesting *C. cinerea* vegetative mycelium and fruiting bodies have been described previously [3].

Isolation and purification of CCL2 from *C. cinerea*. Fruiting bodies or vegetative mycelium of *C. cinerea* were lyophilized and extracted as follows: the material was first homogenized using a mortar before the appropriate amount was weighed in, mixed with an equal volume of glass beads and ground using a FastPrep FP120 device (SAVANT). The powder was extracted by a second FastPrep round in the presence of extraction buffer (1 volume of phosphate buffered saline (PBS), 1 volume of water and 1 mM phenylmethane-sulfonylfluoride) at a ratio of 1 ml/100 mg dry fungal tissue. Insoluble material was spun down at high speed (16100 *g*) at 4°C for 15 minutes, and the supernatant containing the soluble proteins was used as input for affinity chromatography.

Horseradish peroxidase (Sigma) was coupled to CNBr-activated Sepharose 4B (GE Healthcare) according to the manufacturer's protocol. 150 µl of the HRP-sepharose beads were equilibrated with 1.5 ml PBS, and incubated with 500 µl soluble protein extract from fruiting bodies by rotation at 4°C. Flow through was collected by centrifugation, and beads were washed with 1.5 ml PBS, mixed with 100 µl SDS-PAGE sample buffer and boiled at 95°C for 10 minutes to release proteins bound to the matrix. Proteins from input, flow through and beads samples were separated by SDS-PAGE.

Identification of CCL2 by peptide mass fingerprinting. The excised gel piece was washed three times in 200mM ammonium bicarbonate pH 8.0 and dried in a Speed Vac. Protein contained in the sample was reduced with 10mM dithiothreitol in 100mM ammonium bicarbonate at 37 ºC for 1h. After removal of dithiothreitol, cysteines were alkylated with 25mM iodoacetamide in 100mM ammonium bicarbonate at room temperature in the dark for 1h. The iodoacetamide was removed and the gel piece washed five times in 50% acetonitrile. After drying in a Speed Vac, the protein was digested overnight at 37 ºC with 50 ng trypsin in a volume of 50mM ammonium bicarbonate enough to cover the gel piece. Supernatant was transferred to a fresh tube, dried in a Speed Vac and resuspended in 10µl 0.1% trifluoroacetic acid. Tryptic peptides were then desalted with a C18 ZipTip (Millipore, USA) and eluted in the MALDI-MS matrix solution (4 mg/ml α-cyano-4-hydroxy-cinnamic

acid in 70% acetonitrile, 0.1% trifluoroacetic acid). Peptide masses and MS/MS results were used in MASCOT [4] to search a *C. cinerea* genome database.

Quantification of *ccl1* and *ccl2* expression by qRT-PCR. RNA was extracted from 30-50mg of lyophilized mycelium and fruiting bodies from *C. cinerea* using the RNeasy Lipid Tissue Mini Kit (Qiagen). Isolated RNA was DNase-treated using the Qiagen RNase-Free DNase Set (Qiagen). For cDNA synthesis, 5 μg RNA was combined with 2 μl of oligo-dT primer (100 μM) in a volume of 30 μl, and incubated for 10 min at 70°C. The sample was cooled on ice for 10 min. Reverse transcription was carried out with M-MLV Reverse Transcriptase RNase H Minus (Promega), 1x reaction buffer provided with the enzyme, dNTPs (10mM each), 1 μl of Ribolock (Fermentas), in a total final volume of 60 μl. The reaction mixture was incubated at 25°C for 10 minutes, 40°C for 120 minutes and at 70°C for 10 minutes. RNA was hydrolyzed at 65°C for 15 min after the addition of 20 μl of 1 M NaOH. For neutralization, 20 μl of 1M HCl were added. cDNA was purified with the NucleoSpin Extract Kit II (Macherey-Nagel) following the manufacturer's recommendations. Real time PCR was performed in a thermocycler Rotorgene 3000 (Corbett Research) with SensiMix Plus SYBR Kit (Quantace) in a volume of 20 μl. β-tubulin was used as internal control template. Primers for amplification of CCL1, CCL2 and β-tubulin are given in Table S8. PCR conditions were: 95°C for 10 min, followed by 35 cycles of 95 °C for 15 s, 58°C for 30 s, and 72°C for 30 s. Amplicon size and specificity for each primer pair was verified by agarose gel electrophoresis and melting curve analysis. Relative expression ratio of each gene was calculated as described [5]. Standard errors of the mean are based on four technical replicates of each cDNA template and gene.

Cloning of CCL1- and CCL2-encoding genes. The CCL2 genomic locus of *C. cinerea* strain AmutBmut was PCR-amplified from chromosomal DNA using oligonucleotide primers CCL2-seq fwd and CCL2-seq rev (Table S8). CCL1- and CCL2-encoding cDNAs were synthesized and amplified using the OneStep RT-PCR Kit (Qiagen) using total RNA from lyophilized fruiting bodies and primers CCL1-seq fwd and CCL1-seq rev, and CCL2-seq fwd and CCL2-seq rev, respectively. RNA was isolated using the RNeasy Lipid Tissue Mini Kit (Qiagen) according to the manufacturer's protocol. PCR products were ligated into pGEM-T easy vector (Promega), amplified in *E.coli* and sequenced. Plasmids for expression of CCL1 and CCL2 in *E. coli* were constructed by PCR-amplifying the coding regions of the respective cDNA clones using primers carrying suitable restriction sites (CCL1 fwd and CCL1 rev, as well as CCL2 fwd and CCL2 rev; Table S8) and ligating the purified and cut PCR-fragments into accordingly opened pET22b or pET24b (Novagen). Plasmids driving the expression of N-terminally His8-tagged CCL1 and CCL2 were generated accordingly using the same reverse primers but the respective N-His containing forward primers (CCL1-NHis fwd and CCL2-NHis fwd; Table S8).

For the cloning of dTomato-CCL2 fusion, the dTomato coding region was amplified by PCR from plasmid pRO020 [6] using the primers dTomato-fwd and dTomato-rev (Table S8). The PCR product was subcloned into pGEM-T easy and amplified in *E. coli.* The insert was released with *Vsp*I and *BamH*I, and ligated into a pET24b backbone previously linearized with *Nde*I and *BamH*I, resulting in plasmid pET24-dTomato. Finally, the CCL2 coding region was released from above expression plasmids as *Nde*I-*BamH*I fragment and inserted into pET24-dTomato that had previously been opened with the same restriction enzymes.

Expression of CCL1 and CCL2 in *E. coli* and purification of the His8-tagged CCL2. For expression, the various plasmids coding for wild type or mutant forms of the lectins were transformed into *E. coli* strains BL21(DE3). Transformants were cultivated at 37°C in either LB or M9 minimal medium containing 1 g/l $^{15}NH_4Cl$ and 4 g/l glucose (for ^{15}N-labeled proteins) or 1 g/l $^{15}NH_4Cl$ and 2 g/l ^{13}C-glucose (for $^{13}C/^{15}N$ labeled proteins) and 100 mg/l ampicillin or 25 mg/l carbenicillin (for pET22-based plasmids), 50 mg/l kanamycin (for pET24-based plasmids) and 17 mg/l chloramphenicol (for pLysS). CCL2(N91A) had to be expressed in BL21(DE3)/pLysS cells since BL21(DE3)-transformants did not grow on LB nor minimal medium, possibly due to toxicity of this variant for bacterial cells. Expression of CCL1 and CCL2 was induced at OD_{600nm} ~ 0.6-1 by adding 1mM isopropyl β-D-thiogalactoside and further incubation either at 37°C for 4h or at 23°C for 16h. Expression and solubility check of the recombinant proteins was done as previously described (Kunzler et al. 2010). For purification of His8-tagged CCL1 and CCL2, induced cells were collected by centrifugation, washed with water and stored at -20°C. Cells were resuspended in 50mM Na phosphate pH 8.0, 300mM NaCl, 10mM imidazole and disrupted by French Press (SLM Aminco; SLM instruments, Inc. UK) or M-110L Pneumatic Microfluidizer (Microfluidics). The lysate was cleared by centrifugation (30min, 18000 rpm, 4°C) and the supernatant was applied to metal-affinity chromatography using Ni-NTA resins (Qiagen Inc.), following the manufacturer's instructions, except that an additional washing step with a high salt buffer (1 M NaCl, 50 mM Na_2HPO_4, pH 8.0, 10 mM imidazole) was added. After elution, the buffer of the protein was exchanged (by dialysis or desalting) to either NMR buffer (50 mM KH_2PO_4 pH 5.7, 150 mM NaCl) or TOX buffer (50 mM Na_2HPO_4 pH 6.0, 150mM NaCl). Identity and purity were verified by SDS-PAGE.

CCL2-GlcNAcβ1,4[Fucα1,3]GlcNAc complex formation. The complexes were prepared by titrating the concentrated carbohydrate solution of typically 10 mM into a ~1 mM solution of CCL2 in NMR buffer (50 mM KH_2PO_4 pH 5.7, 150 mM NaCl) until a 1:1 stoichiometry was reached. Subsequently, the pH was lowered to 4.7 using 10% deuterated acetic acid to avoid precipitation. Protein concentrations were determined by UV

spectroscopy (ε_{280} = 41940 M^{-1} cm^{-1}). For measurements in D_2O samples were lyophilized and dissolved in D_2O containing the same amount of 10% deuterated acetic acid (in D_2O) as the sample originally contained in H_2O.

NMR spectroscopy. NMR spectra were acquired on Avance III 500, 600, 700 and Avance 900 Bruker spectrometers equipped with inverse triple resonance cryogenetic probes and pulse field gradient accessory. In addition an Avance III 750MHz with an inverse triple resonance room temperature probe and pulse field gradient accessory was used. Unless indicated otherwise, data were measured at 310 K. NMR data were processed using Topspin 2.1 (Bruker) and analyzed with Sparky [7]. The ^{1}H,^{13}C,^{15}N chemical shifts of the protein, free and in complex, were assigned using 2D ^{1}H-^{15}N-HSQC, 2D ^{1}H-^{13}C-HSQC, 3D HNCA, 3D CBCACONH, 3D HNCACB, 3D HNCO, 3D (H)CCH-TOCSY, 3D ^{15}N-edited NOESY-HSQC, 3D ^{13}C-edited NOESY-HSQC, 2D NOESY and 2D TOCSY experiments [8]. Assignment of carbohydrate resonances of the complex was achieved using NOE correlations and exchange peaks with signals of the free carbohydrate since neither TOCSY based spectra nor a natural abundance ^{13}C-HSQC did show signals of the bound carbohydrate. The following spectra were used for this purpose 2D ^{1}H-^{1}H NOESY, 2D ^{13}C/^{15}N F1-filtered NOESY and 2D ^{13}C F1-filtered F2-filtered NOESY [9]. The assignments of intermolecular NOEs were derived from 3D ^{13}C F1-edited, F3-filtered NOESY-HSQC [10] spectra of the protein-carbohydrate complex. All NOESY spectra of the were recorded with a mixing time of either 100 ms (free protein) or 120 ms (complex). The 3D TOCSY spectrum was recorded with a mixing time of 23 ms and 2D TCOSY spectra with a mixing time of 15 or 60 ms.

Structure calculation and refinement. Initial CCL2 structures (free and bound) were generated using the AtnosCandid software package [11,12] using three 3D NOESY spectra ($^{13}C^{ali}$-edited, $^{13}C^{aro}$-edited and ^{15}N-edited) and a 2D NOESY spectrum. The automatically generated upper limit restraints file was used as a starting point for the first level of manually refining the protein structures by a simulated annealing protocol using the Cyana package [11]. Preliminary structures of the CCL2-carbohydrate complex were generated using the Cyana package with the above mentioned restraints and manually assigned intermolecular and intra-carbohydrate NOE distance constraints. To create the topology of the carbohydrate for the Cyana library file an initial model was generated by SWEET [13]. The carbohydrate spacer was truncated to a methyl group since we observed only few weak intermolecular NOEs between the spacer and N91 indicating that the spacer is projecting away from the protein and does not fold back. 300 structures were generated by CYANA starting from random carbohydrate and protein starting structures using 16,000 simulated annealing steps. Structures improved when backbone angle restraints derived form chemical shifts using TALOS+ [14] were subsequently added to both AtnosCandid and Cyana. At later stages of the refinement, hydrogen-bond restraints

(only within protein) were added. An ensemble of 30 structures, selected based on the lowest target function, served for the refinement in AMBER 9.0 [15]. Structures of CCL2 free and in complex were refined in implicit solvent using NOE-derived distances, torsion angles and hydrogen bond restraints as summarized in Table 2. In all AMBER calculations, the force-field 98 based on the Cornell et al. force-field [16] was used along with the generalized Born model [17] to mimic solvent. A 20-ps simulated annealing protocol consisting of 22,000 steps was used. NMR restraints were applied as square-well penalty functions with the force constants 50 kcal mol^{-1} $Å^{-2}$ and 200 kcal mol^{-1} rad^{-2} for distance constraints and torsion angles, respectively. Relative weights of the valence-angle energy, torsion energy and 'improper' torsional terms were gradually increased during the simulated annealing to maintain the planarity of aromatic rings and proper local geometries. After the simulated annealing protocol a short energy minimization of 400 cycles was followed (a combination of steepest-descents minimization followed by conjugate gradient technique). 20 conformers with the lowest restraint violations were selected to obtain the final ensemble of structures. The quality of the complex was analyzed by PROCHECK-NMR [18] and CARP [19]. Figures of the complex structure were prepared using MOLMOL [20].

Preparation of the *C. elegans fut-1 fut-6* double mutant (F1F6) and PCR screening. All parent strains were obtained from the *C. elegans* gene knock-out consortium via the Caenorhabditis Genetics Centre (CGC). The experiment was designed based on the genetic crossing principle. Three L4 hermaphrodites of VC 585 *fut-1 (ok892)* were transferred onto small NGM plates and mated with twelve N2 male adults; male adults of the F1 generation were mated with RB 706 *fut-6 (ok475)* L4 hermaphrodites as above, in order to gain the F2 generation. Per plate, a previously singled hermaphrodite was isolated after egg laying and transferred into 7 μl of lysis buffer [50mM KCl, 10mM Tris-HCl (pH 8.3), 2.5mM MgCl2, 0.45% NP40, 0.45% Tween 20, 60 μg/ml Proteinase K], then incubated at 60°C for 1 hour to extract genomic DNA. Proteinase K was heat inactivated at 95°C for 15 mins. Primer set fut1 (A,B,C) was designed for detecting *fut-1* genotypes; whereas Primer set fut6 (A,B,C) was designed for detecting *fut-6* genotypes. Multiplex PCR reactions were carried out in a 20 μl standard reaction system with 1 μl gDNA, 1.2 μl of a primer sets mixture (fut1 + fut6, 20μM of each primer), 7.8 μl of H2O, and 10 μl of GoTaq Green Master Mix (Promega). In the case of performing triplex PCRs to detect a single gene in one PCR reaction, 0.6 μl of a primer set was added, therefore H2O was increased to 8.4 μl. The PCR program was: 95°C for 3 mins, followed by 30 cycles at 95°C for 30 s, 52°C for 30 s, 72°C for 30 s (1000 base pairs per minute). PCR screening was performed as follows: firstly, for screening of individuals carrying *fut-1/+;fut-6/+* genotype, 30 hermaphrodites of the F2 generation were transferred singly to small NGM plates for laying eggs, prior to gDNA extraction and multiplex-PCR; secondly, 100 progeny from the fut-1/+;fut-6/+

positive plates were then singled, and screened by multiplex-PCR after eggs were laid, in order to seek the *fut-1/fut-1;fut-6*/+ and/or *fut-1*/+*;fut-6/fut-6* genotype; finally, 40 progeny from positive plates were singled; again, after eggs were laid, the genotypes were checked by triplex-PCR. The PCR products were loaded on to 3% agarose gels and analyzed by electrophoresis in TAE buffer at 100 volts for 75 mins. A product size of 270 nt indicated the wild type form of the *fut-1* gene, while 320 nt indicated the mutant form; a product size of 200 nt indicated the wild type form of the *fut-6* gene, while 158 nt indicated the mutant form.

Localization of CCL2-binding in *C. elegans*. A stationary culture of *E.coli* containing the dTomato-CCL2 expression plasmid was used to seed NGM plates containing 1mM IPTG and 50 mg/l kanamycin and incubated overnight at 23°C for protein expression before addition of nematodes. L4 staged *C. elegans* worms of indicated genotypes were transferred to the plates. After 36h the animals were transferred to standard *E. coli* OP50 to allow unbound lectin leave the intestinal tract. After 1 hr, 10 worms were placed on 3% agarose pads in M9, anaesthesized with 3–5 mM levamisole (Sigma) and mounted under a coverslip for observation using a Leica DM-RA or Zeiss Axiovert 200 microscope equipped with DIC (Nomarski) optics and standard epifluorescence with a DsRed filter set for detection of dTomato. Pictures were taken with a Hamamatsu ORCA-ER camera. Images were false-coloured using OpenLab software.

References

1. Sambrook J, Russell DW (2001) Molecular cloning: A laboratory manual. New York: Cold Spring Harbor. Laboratory Press.
2. Stiernagle T (2006) Maintenance of C. elegans. WormBook 11: 1-11.
3. Wälti MA, Villalba C, Buser RM, Grunler A, Aebi M, et al. (2006) Targeted gene silencing in the model mushroom Coprinopsis cinerea (Coprinus cinereus) by expression of homologous hairpin RNAs. Eukaryot Cell 5: 732-744.
4. Perkins DN, Pappin DJ, Creasy DM, Cottrell JS (1999) Probability-based protein identification by searching sequence databases using mass spectrometry data. Electrophoresis 20: 3551-3567.
5. Schefe JH, Lehmann KE, Buschmann IR, Unger T, Funke-Kaiser H (2006) Quantitative real-time RT-PCR data analysis: current concepts and the novel "gene expression's CT difference" formula. J Mol Med 84: 901-910.
6. Vinck A, de Bekker C, Ossin A, Ohm RA, de Vries RP, et al. (2011) Heterogenic expression of genes encoding secreted proteins at the periphery of Aspergillus niger colonies. Environ Microbiol 13: 216-225.
7. Goddard TD, Kneller DG SPARKY 3. University of California, San Francisco.
8. Sattler M, Schleucher J, Griesinger C (1999) Heteronuclear multidimensional NMR experiments for the structure determination of proteins in solution employing pulsed field gradients. Prog Nucl Magn Reson Spectrosc 34: 93-158.
9. Peterson RD, Theimer CA, Wu H, Feigon J (2004) New applications of 2D filtered/edited NOESY for assignment and structure elucidation of RNA and RNA-protein complexes. J Biomol NMR 28: 59-67.

10. Dominguez C, Schubert M, Duss O, Ravindranathan S, Allain FH (2011) Structure determination and dynamics of protein-RNA complexes by NMR spectroscopy. Prog Nucl Magn Reson Spectrosc 58: 1-61.
11. Herrmann T, Guntert P, Wuthrich K (2002) Protein NMR structure determination with automated NOE assignment using the new software CANDID and the torsion angle dynamics algorithm DYANA. J Mol Biol 319: 209-227.
12. Herrmann T, Guntert P, Wuthrich K (2002) Protein NMR structure determination with automated NOE-identification in the NOESY spectra using the new software ATNOS. J Biomol NMR 24: 171-189.
13. Bohne A, Lang E, von der Lieth CW (1999) SWEET - WWW-based rapid 3D construction of oligo- and polysaccharides. Bioinformatics 15: 767-768.
14. Shen Y, Delaglio F, Cornilescu G, Bax A (2009) TALOS+: a hybrid method for predicting protein backbone torsion angles from NMR chemical shifts. J Biomol NMR 44: 213-223.
15. Case DA, Cheatham TE, Darden T, Gohlke H, Luo R, et al. (2005) The Amber biomolecular simulation programs. J Comput Chem 26: 1668-1688.
16. Cornell WD, Cieplak P, Bayly CI, Gould IR, Merz KM, et al. (1995) A 2nd Generation Force-Field for the Simulation of Proteins, Nucleic-Acids, and Organic-Molecules. J Am Chem Soc 117: 5179-5197.
17. Bashford D, Case DA (2000) Generalized born models of macromolecular solvation effects. Annu Rev Phys Chem 51: 129-152.
18. Laskowski RA, Rullmann JAC, MacArthur MW, Kaptein R, Thornton JM (1996) AQUA and PROCHECK-NMR: Programs for checking the quality of protein structures solved by NMR. J Biomol NMR 8: 477-486.
19. Lütteke T, Frank M, von der Lieth CW (2005) Carbohydrate Structure Suite (CSS): analysis of carbohydrate 3D structures derived from the PDB. Nucleic Acids Res 33: D242-246.
20. Koradi R, Billeter M, Wüthrich K (1996) MOLMOL: a program for display and analysis of macromolecular structures. J Mol Graph 14: 51-55.
