# Supplementary material for: Correction: Plasticity of the β-Trefoil Protein Fold in the Recognition and Control of Invertebrate Predators and Parasites by a Fungal Defence System
Source: PLoS Pathog. 2012 Aug 15;8(8):10.1371/annotation/088ea07b-d578-4586-9707-160143d4f1be. doi: 10.1371/annotation/088ea07b-d578-4586-9707-160143d4f1be (PMC3439293; doi:10.1371/annotation/088ea07b-d578-4586-9707-160143d4f1be)

Synthesis and characterisation of GlcNAcβ1,4[Fucα1,3]GlcNAcβ1-O-(CH₂)₅-COOH

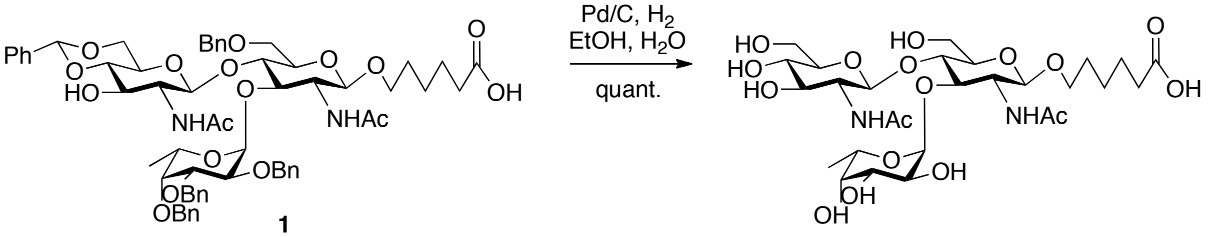

1[1] (28 mg, 24 µmol) was dissolved in a mixture of ethanol (2 mL) and water (1 mL). Pd/C 10% (12 mg) was added and the solution was stirred. Vacuum and H_2 were alternated and the mixture was allowed to stir under H_2 overnight. The mixture was filtered off through Celite and concentrated. The residue was dissolved in water and filtered through a 0.45 µm syringe filter and concentrated. The residue was purified on a G15 column to give, after lyophilisation, 14 mg of the desired product (99%) as a white solid. $[a]^{25}_{D}$ -80 (c 1.0, H_2O). MS FAB+-HRMS m/z $[M+C_2H_5]^+$ calcd for $C_{30}H_{53}O_{17}N_2$ 713.3344, found 713.3357.

[1] "Synthesis of cross-reactive carbohydrate determinants fragments as tools for in vitro allergy diagnosis", Collot, M.; Wilson I. B. H.; Bublin, M.; Hoffmann-Sommergruber, K.; Mallet, J.-M. *Bioorg. Med. Chem.* **2011**, *19*, 1306-1320.

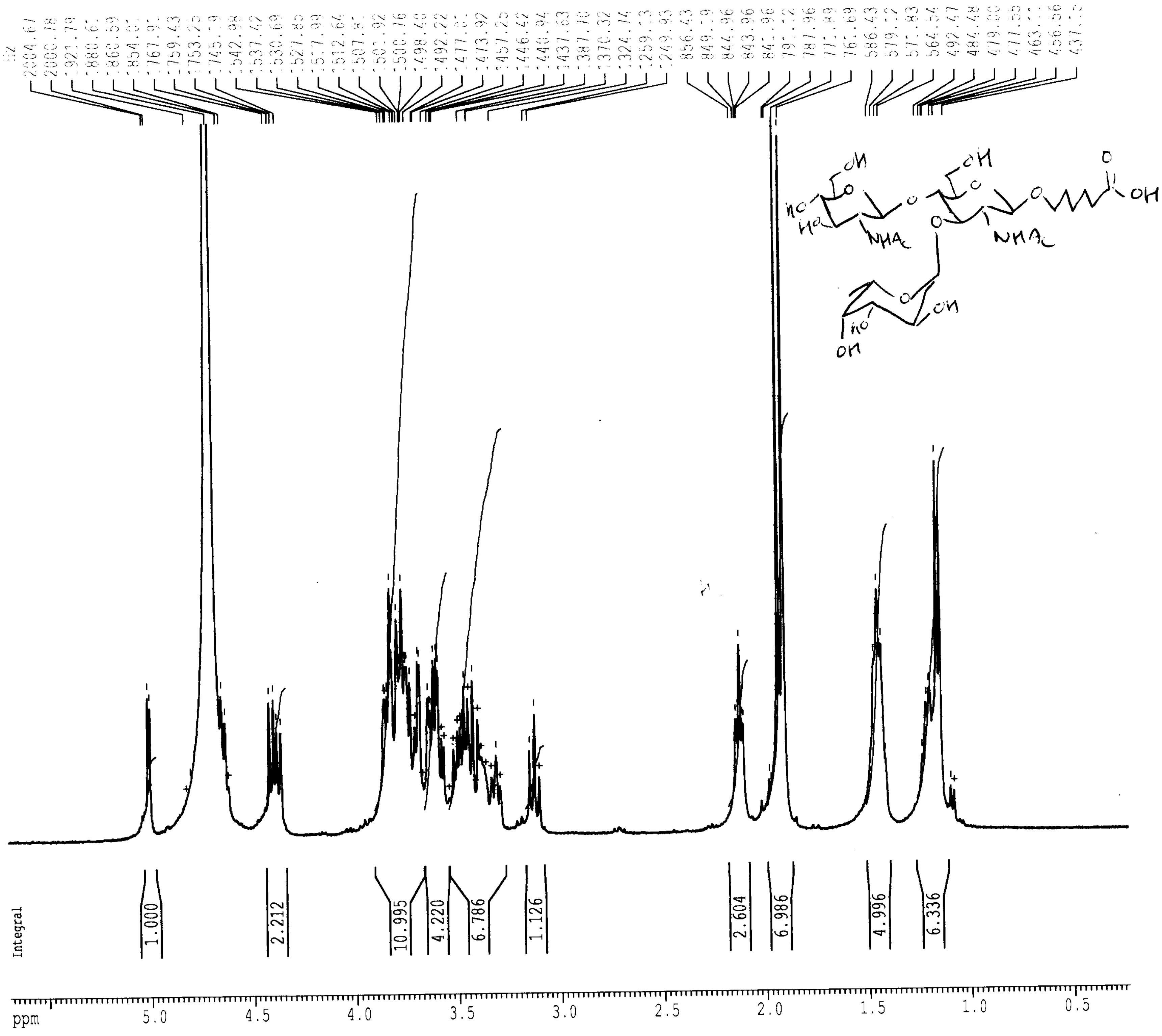

Current Data Parameters
NAME mayeulmulti
EXPNO 1
PROCNO 1

F2 - Acquisition Parameters
Date_ 500000
Time 21.30
INSTRUM spect
PROBHD 5 mm 1H/13C
PULPROG zg30
TD 16384
SOLVENT CDCl3
NS 32
DS 2
SWH 4006.410 Hz
FIDRES 0.244532 Hz
AQ 2.0447731 sec
RG 228.1
DW 124.800 usec
DE 4.50 usec
TE 300.0 K
D1 0.10000000 sec
P1 9.50 usec
DE 4.50 usec
SFO1 400.1319206 MHz
NUC1 1H
PL1 -3.00 dB

F2 - Processing parameters
SI 16384
SF 400.1300000 MHz
WDW EM
SSB 0
LB 0.00 Hz
GB 0
PC 0.10

1D NMR plot parameters
CX 22.00 cm
F1P 5.693 ppm
F1 2278.08 Hz
F2P 0.231 ppm
F2 92.28 Hz
PPMCM 0.24831 ppm/cm
HZCM 99.35441 Hz/cm

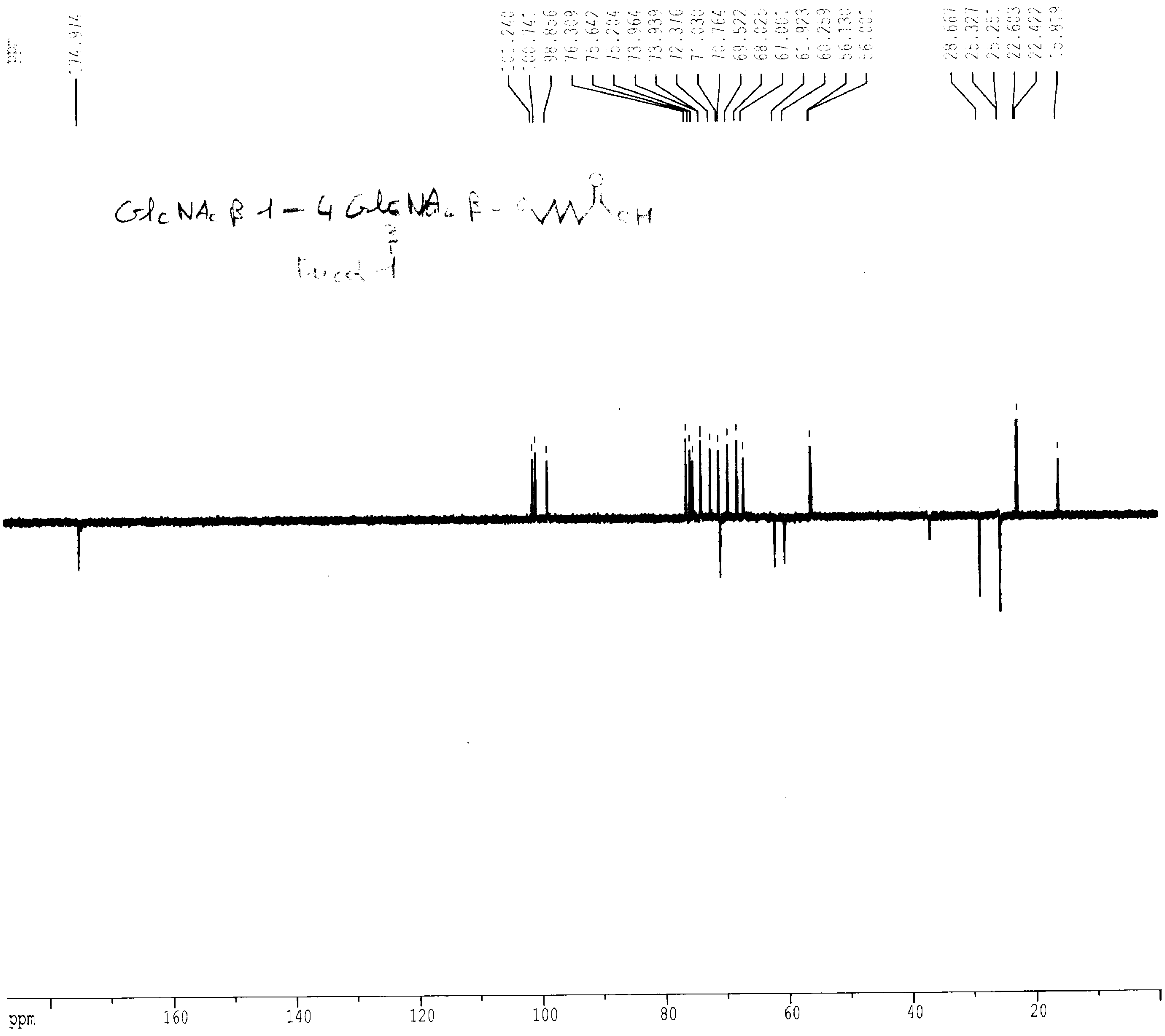

Current Data Parameters
NAME mayeulmulti
EXPNO 2
PROCNO 1

F2 - Acquisition Parameters

Date_	500000
Time	3.48
INSTRUM	spect
PROBHD	5 mm 1H/13C
PULPROG	jmod2.php
TD	65536
SOLVENT	CDCl3
NS	8192
DS	4
SWH	24154.590 Hz
FIDRES	0.368570 Hz
AQ	1.3566452 sec
RG	5792.6
DW	20.700 usec
DE	4.50 usec
TE	300.0 K
P1	26.50 usec
DELTA	0.0000169 sec
D20	0.00714000 sec
PL12	17.50 dB
D1	1.29999995 sec
CPDPRG2	waltz16
PCPD2	100.00 usec
SFO2	400.1317000 MHz
NUC2	1H
PL2	120.00 dB
D13	0.00000300 sec
SFO1	100.6237964 MHz
NUC1	13C
PL1	0.00 dB
P2	53.00 usec
DE	4.50 usec

F2 - Processing parameters

SI	65536
SF	100.6127290 MHz
WDW	EM
SSB	0
LB	0.30 Hz
GB	0
PC	0.30

1D NMR plot parameters

CX	22.00 cm
F1P	187.110 ppm
F1	18825.66 Hz
F2P	-0.233 ppm
F2	-23.45 Hz
PPMCM	8.51560 ppm/cm
HZCM	856.77777 Hz/cm

[Mass Spectrum]
Data : COLLOT-MC455-CI+-NH3-HR001 Date : 07-Mar-2007 17:28
Sample: -
Note : -
Inlet : Direct Ion Mode : DCI+
Spectrum Type : Normal Ion [EF-Linear]
RT : 9.16 min Scan# : (100,101)
BP : m/z 713.3357 Int. : 1.02
Output m/z range : 640.0000 to 740.0000 Cut Level : 0.00 %

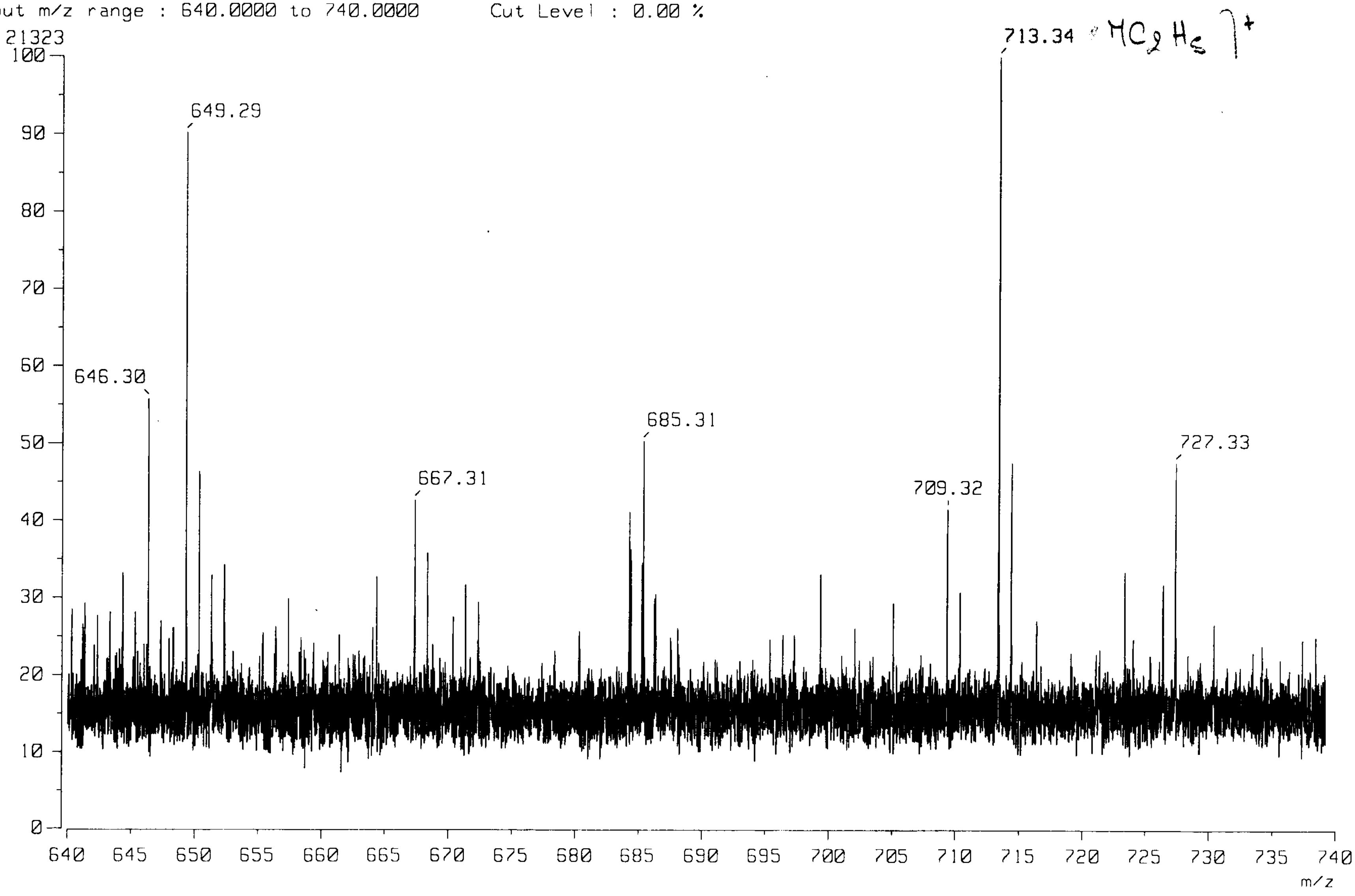

Supplement: Supplementary file 1 [file ppat.088ea07b-d578-4586-9707-160143d4f1be.s001.pdf]
